# Antagonist Targeting the Species‐Specific Fatty Acid Dehydrogenase/Isomerase FabX for Anti‐*H. pylori* Infection

**DOI:** 10.1002/advs.202414844

**Published:** 2025-03-16

**Authors:** Lin Zhang, Xiaoxue Ruan, Xudong Hang, Ding Heng, Chang Cai, Liping Zeng, Guoxin Zhang, Lu Zhou, Hongkai Bi, Liang Zhang

**Affiliations:** ^1^ Department of Pharmacology and Chemical Biology School of Medicine Shanghai Jiao Tong University Shanghai 200025 China; ^2^ Department of Medicinal Chemistry School of Pharmacy Fudan University Shanghai 201203 China; ^3^ Department of Pathogen Biology and Jiangsu Key Laboratory of Pathogen Biology Nanjing Medical University Nanjing Jiangsu 211166 China; ^4^ NHC Key Laboratory of Tropical Disease Control School of Tropical Medicine Hainan Medical University Haikou Hainan 571199 China; ^5^ Department of Gastroenterology The First Affiliated Hospital of Nanjing Medical University Nanjing Jiangsu 210009 China; ^6^ Quzhou Fudan Institute Quzhou 324002 China; ^7^ Department of Chemical Biology School of Chemistry and Chemical Engineering Shanghai Jiao Tong University Shanghai 200240 China

**Keywords:** dehydrogenase/isomerase, fatty acid biosynthesis, *H. pylori*, inhibitor, ROS

## Abstract

*Helicobacter pylori* (*H. pylori*) is a group‐1 definite pathogenic carcinogen that infects approximately half of the global population, yet no species‐specific chemotherapy has yet been developed. It is previously discovered that *H. pylori* encodes an atypical dehydrogenase/isomerase FabX in the Type‐II fatty acid biosynthesis pathway to produce unsaturated fatty acids (UFA) as well as superoxide (ROS). Here, it is demonstrated that FabX is essential for *H. pylori* growth and gastric colonization by retaining UFA synthesis and producing ROS, respectively, and is a species‐specific anti‐*H. pylori* drug target. The first small molecule inhibitor FBX‐1991 against FabX, which inhibits the enzymatic activity with an *IC*_50_ value of 0.158 × 10^−6^ m in vitro, is developed. FBX‐1991 binds inside the catalytic tunnel of FabX, disrupts the conformation of the key catalytic loop, and prevents the insertion of the acyl substrate for catalysis. Further in vivo studies suggest that FBX‐1991 inhibits the *H. pylori* growth by partially inhibiting UFA synthesis and ROS excretion through targeting FabX. This study identifies a species‐specific anti‐*H. pylori* drug target, FabX, and discovers the first highly potent and selective FabX inhibitor against *H. pylori* infection, which provides the molecular basis for developing species‐specific anti*‐H. pylori* chemotherapy.

## Graphical abstract

This study demonstrates that the atypical dehydrogenase/isomerase FabX in Type‐II fatty acid biosynthesis pathway is essential for *H. pylori* infection by retaining unsaturated fatty acid synthesis and producing superoxide (ROS). A small molecule FBX‐1991 that specifically targets FabX to inhibit *H. pylori* growth and gastric colonization by reducing unsaturated fatty acid synthesis as well as ROS generation and excretion is also discovered.

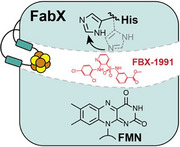

## 1 Introduction

The Gram‐negative microaerophilic bacterium *Helicobacter pylori* (*H. pylori*) is a human gastric pathogen that has been classified by the International Agency for Research on Cancer (IARC) as a group‐1 definite carcinogen and is the third most common cause of global cancer‐related mortality.^[^ ^1^, ^2^ ^]^ It infects approximately half of the global population and colonizes at human gastric mucosa, leading to lifelong, chronically progressive and severe gastroduodenal pathologies including gastritis, peptic ulcers, gastric atrophy, gastric intestinal metaplasia, and in particular, mucosa‐associated lymphoid tissue lymphoma and gastric adenocarcinoma.^[^ ^3^ ^]^ Current globally recognized clinical quadruple chemotherapy against *H. pylori* infection includes the combination of two broad‐spectrum antibiotics (i.e., amoxicillin and clarithromycin), a bismuth‐based drug (i.e., bismuth pectin), and a gastric acid proton pump inhibitor (i.e., H^+^/K^+^‐ATPase inhibitors, PPI).^[^ ^1^, ^4^, ^5^ ^]^ Although such therapy has shown favorable clinical outcomes, long‐term antibiotic treatment resulted in several intolerable side effects, including antibiotic resistance, allergic reactions, gastrointestinal symptoms, and dysbiosis.^[^ ^6^, ^7^ ^]^ The development of highly selective pharmaceuticals against *H. pylori* has gained worldwide attention.

Type‐II fatty acid biosynthesis pathway (FAS‐II) is a fundamental metabolic pathway in prokaryotes.^[^ ^8^ ^]^ The saturated (SFA) and unsaturated fatty acids (UFA) it produces are essential biomaterials for bacterial membrane assembly and energy reservoirs, making the enzymes involved in FAS‐II have been considered as well‐known antimicrobial drug targets.^[^ ^9^ ^]^ In FAS‐II, both SFA and UFA synthesis are initiated by the installation of a 4′‐phosphopantetheine (Ppant) arm on the conserved serine residue (Ser36 in *H. pylori*) of the acyl carrier protein (ACP) and a malonyl‐group at the terminal sulfhydryl of the Ppant arm, followed by a similar repeated process of acyl chain elongation via successive decarboxylative Claisen condensation (first step), ketoreduction (second step), dehydration (third step), and enoyl‐reduction (fourth step) catalysis (Figure S1, Supporting Information).^[^ ^8^, ^10^, ^11^, ^12^ ^]^ In addition, either a classical dehydratase/isomerase FabA (in most Gram‐negative bacteria), FabN (*Enterococcus faecalis* and *Tetracoccus salidis*), or FabQ (*Aerococcus viridans*),^[^ ^13^, ^14^, ^15^ ^]^ or a monofunctional isomerase FabM (*Streptococcus pneumoniae*) was utilized in the third dehydration step for the introduction of a *cis*‐3 double bond on the synthesized acyl chain of ACP for UFA synthesis.^[^ ^16^, ^17^ ^]^ Remarkably, the genome of *H. pylori* lacks these genes, and the mystery of how *H. pylori* produces UFA remains unknown.

**Figure 1:**
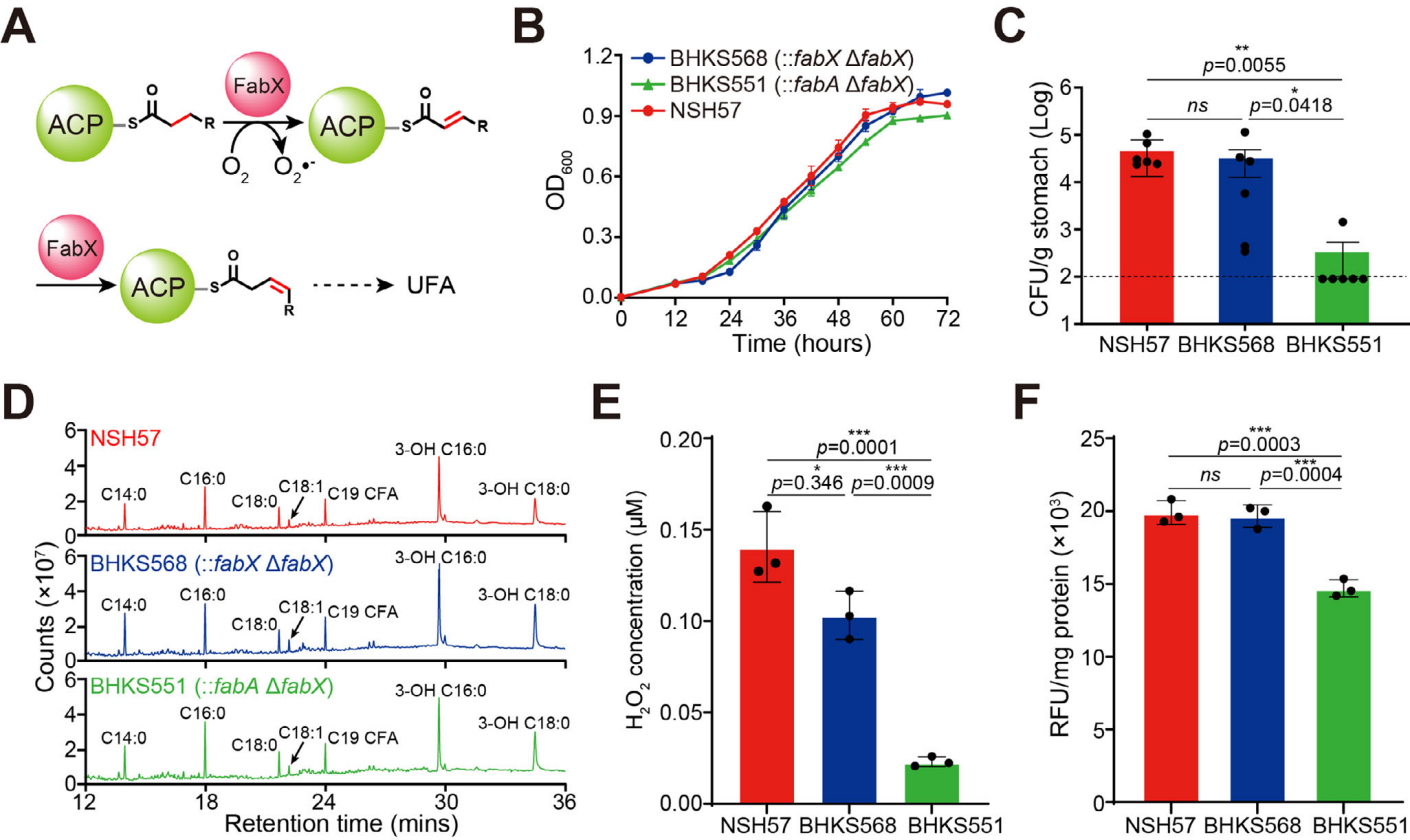
FabX is essential for *H. pylori* gastric colonization. A) Schematic diagram of FabX from *H. pylori* catalyzing saturated acyl‐ACP dehydrogenation and isomerization for UFA synthesis. B) Growth curves of *H. pylori* strains NSH57 (wild type, red curve), BHKS568 (NSH57 IR0203::*fabX* Δ*fabX*, blue curve), and BHKS551 (NSH57 IR0203::*fabA* Δ*fabX*, green curve) in BHI broth containing 10% FCS. Data represent the mean (±SD) of three independent experiments. C) Mouse stomach colonization levels of *H. pylori* NSH57, BHKS568, and BHKS551 strains. Mice were inoculated with *H. pylori* strains four times (2‐d intervals). After two weeks, the stomachs were harvested, homogenized, diluted, and plated, and the number of CFU was determined 3–5 d after harvest. Each point represents the CFU count from one mouse stomach, and the solid horizontal lines represent the geometric means of the colonization numbers for each group. Error bars represent the standard deviations derived from six mice per group, as analyzed by one‐way ANOVA with Tukey's multiple comparisons test. The black dashed line indicates the detection limit of the assay (100 CFU g^−1^ stomach). D) Gas chromatograms of fatty acid methyl esters from the whole cells of *H. pylori* strains. The fatty acids were derivatized to their methyl esters and then analyzed by GC‐MS. E) H_2_O_2_ released by *H. pylori* strains. The strains were cultured to an OD_600_ of 0.3, and the culture supernatants were collected and directly subjected to H_2_O_2_ quantification. F) ROS quantification in *H. pylori* strains. The strains were cultured to an OD_600_ of 0.3, and the cultures were directly subjected to ROS quantification. The data represent the mean (±SD) of three independent experiments with statistical analyses performed by one‐way ANOVA with Tukey's multiple comparisons test.

We later discovered that *H. pylori* encodes an atypical species‐specific bifunctional dehydrogenase/isomerase enzyme FabX, which introduces a *cis*‐double bond to the saturated acyl substrate of ACP via a backtracking mechanism that expectedly reverses the conventional fatty acid elongation direction (**Figures** **1**A and S1, Supporting Information).^[^ ^18^ ^]^ FabX utilizes a nitronate monooxygenase (NMO) flavoprotein family conserved flavin mononucleotide (FMN) cofactor and an active residue His182 inside the catalytic tunnel to extract electrons/protons from the acyl substrate for dehydrogenation and *cis*‐double bond formation, storing the captured electrons in FMN and [4Fe‐4S] cluster cofactors, respectively. Subsequently, FabX transfers the stored electrons back to the oxygen molecule located in the oxyanion hole between FMN and His182, generating superoxide (ROS), which is further excreted by *H. pylori* as a major source of peroxide, likely enabling its pathogenic function of gastric mucosa corrosion.^[^ ^19^ ^]^ We have also demonstrated that genes encoding FabX and its homologous enzymes rarely exist in the genomes of human associated bacteria such as *Escherichia coli *(*E. coli*), and knocking out the *fabX* gene is lethal to *H. pylori*, suggesting that FabX could be a potential drug target against *H. pylori*, as targeting FabX with inhibitors could be more specific to the pathogen without significantly disrupting the healthy gut microbiota.^[^ ^18^, ^19^ ^]^

In this study, we demonstrated that FabX not only synthesizes UFA for *H. pylori* growth, but also produces ROS by catalyzing the dehydrogenation of acyl substrate carried by ACP for *H. pylori* gastric colonization, and is a specific anti‐*H. pylori* drug target. We then developed the lead compound FBX‐1991, which specifically targets FabX to inhibit its enzymatic activity and partially inhibiting in vivo UFA synthesis and ROS production. These results facilitate better understanding of the pathogenic mechanisms of *H. pylori* in gastric infections, and provide the molecular basis for the development of species‐specific anti*‐H. pylori* chemotherapy.

## 2 Results

### 2.1 FabX Catalysis Produces ROS That Is Essential for *H. pylori* Gastric Colonization

To validate that FabX is an effective drug target against *H. pylori*, we used a mouse‐adapted *H. pylori* strain NSH57 and two strains derived from NSH57: BHKS551 (the *fabX* gene was replaced by the β‐hydroxyacyl‐ACP dehydratase/isomerase *fabA* gene from *E. coli*, NSH57 IR0203::*fabA* Δ*fabX*) and BHKS568 (NSH57 IR0203::*fabX* Δ*fabX*).^[^ ^19^ ^]^ The bacterial proliferation assay showed that these strains had similar growth phenotypes, indicating that the replacement did not impair the growth of *H. pylori* (Figure 1B). Unexpectedly, subsequent infection assays with these strains in C57BL/6 mice and quantitative analysis of gastric homogenate cultures showed that the viable cell count of strain BHKS551 was 187‐fold lower than that of strain BHKS568, whereas BHKS568 and NSH57 had a similar colonization phenotype (Figure 1C). These observations suggested that FabX, but not FabA, specifically contributes to gastric colonization of *H. pylori*.

To investigate the mechanism of FabX in gastric colonization, we determined the cellular fatty acid composition profiles of these strains. The results showed that the strains had no significant variations in fatty acid composition, indicating that these profiles do not affect the colonization phenotypes (Figure 1D). We then measured the levels of hydrogen peroxide and ROS levels excreted by these strains in the media, as we have demonstrated that FabX catalysis produces ROS in addition to UFA.^[^ ^18^, ^19^ ^]^ Interestingly, strain BHKS551, in which *fabX* was replaced by *fabA*, released ≈5.1‐fold less hydrogen peroxide to the media than BHKS568, and the ROS level of BHKS551 is remarkably reduced consistently (Figure 1E,F). These results indicate that the ROS generated by FabX catalysis contributes to *H. pylori* gastric colonization, and FabX is, therefore, a specific anti‐*H. pylori* drug target.

### 2.2 Discovery of Primary Inhibitor FBX‐1 against FabX

**Figure 2:**
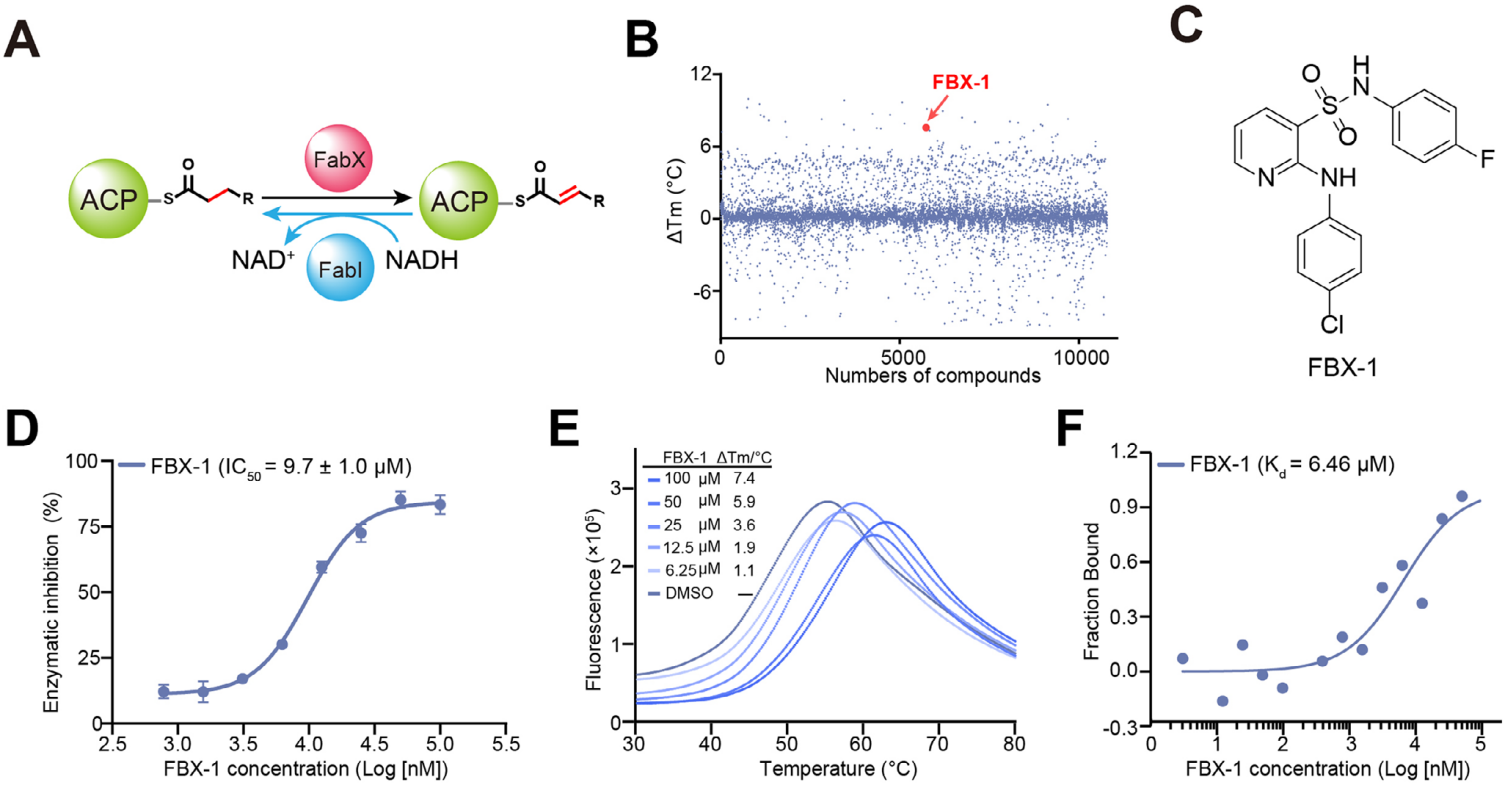
Identification of the hit compound FBX‐1 against FabX. A) The multienzymatic cascade reaction system for high‐throughput screening. B) The scatter diagram from the high‐throughput screening against FabX by using protein thermal shift assay (PTSA). C) The chemical structure of hit compound FBX‐1. D) Enzymatic inhibition evaluation of FBX‐1 against FabX. E) Dose‐dependent protein thermal shift assay of FBX‐1 against FabX. F) Binding affinity evaluation of FBX‐1 against FabX by using MST.

To discover small molecule inhibitors targeting FabX, we performed protein thermal shift assay (PTSA) based high‐throughput screening against an in‐house chemical library of 11000 small molecule compounds, and established a multienzymatic cascade reaction system by incorporating the enoyl acyl carrier protein reductase FabI from *H. pylori* into the hit validation system, where FabI catalyzes the reduction of FabX catalytic product enoyl‐ACP in the fourth step of the elongation cycle, accompanying the elimination of NADH (**Figure** **2**A,B). The results suggested that the compound FBX‐1 is a potential primary hit for FabX (Figure 2C). FBX‐1 exhibited inhibitory activity against FabX with a half‐maximal inhibitory concentration (*IC*_50_) of 9.7 ± 1.0 × 10^−6^ m (Figure 2D). It increases the stability of FabX protein in a dose‐dependent manner, enhancing it by ≈7.4 °C (ΔTm) at a concentration of 100 × 10^−6^ m (Figure 2E), and binds to FabX with a *K*_d_ value 6.46 × 10^−6^ m according to the results from Microscale Thermophoresis (MST) evaluation (Figure 2F). These results indicated that FBX‐1 is a FabX inhibitor that specifically binds to FabX and inhibits its enzymatic activity with moderate potency.

### 2.3 Chemical Optimization of FBX‐1 Leads to a Highly Potent FabX Inhibitor FBX‐1991

**Figure 3:**
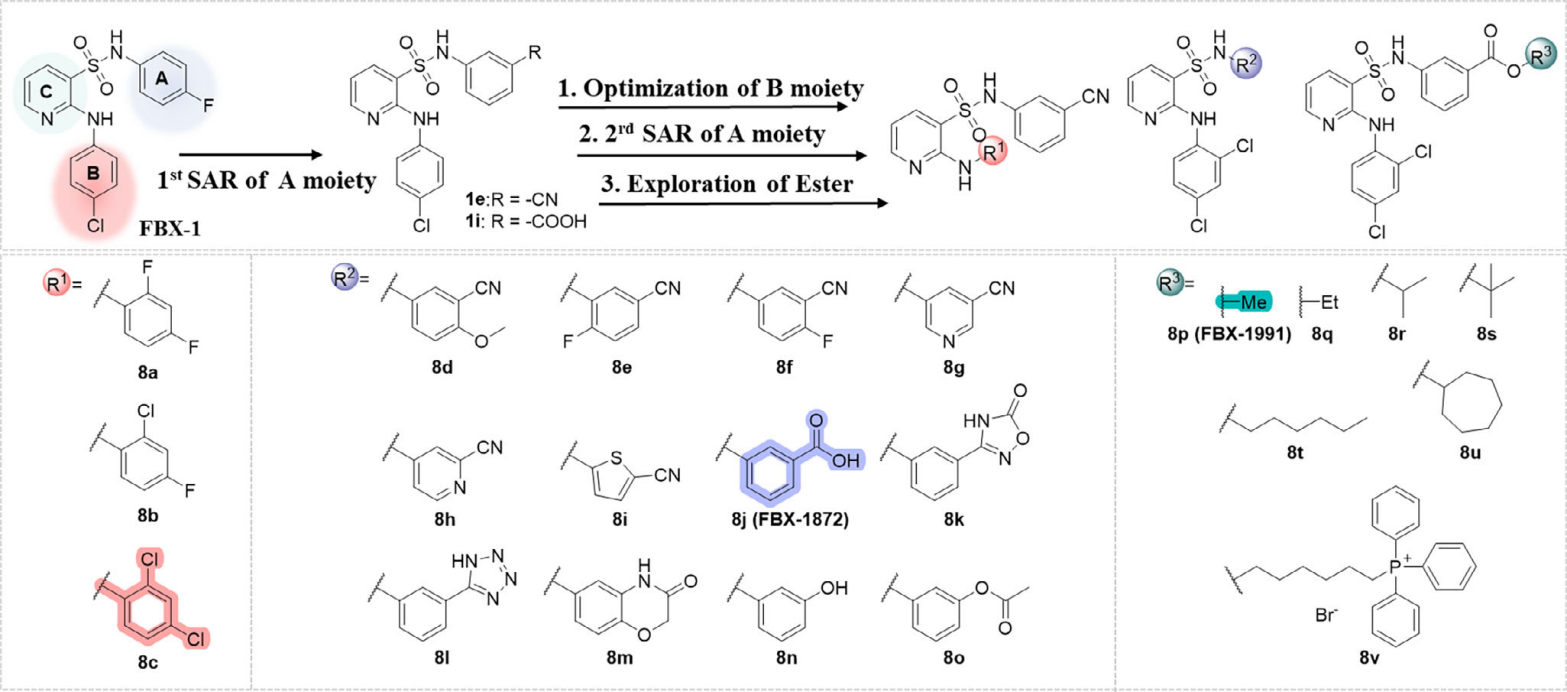
Design strategy and chemical structures of FBX‐1 derivatives.

**Scheme 1:**
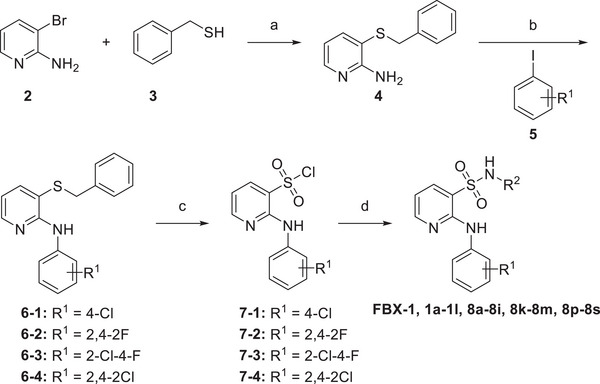
General synthetic route of compounds **FBX‐1, 1a‐1l, 8a‐8i, 8k‐8m, 8p‐8s** *^a^*. *^a^* Reagents and conditions: a) Pd_2_(dba)_3_, Xantphos, toluene, 80 °C, overnight; b) Pd_2_(dba)_3_, Xantphos, Cs_2_CO_3_, 1,4‐dioxane, 100 °C, overnight; c) NCS, AcOH/H_2_O (V/V = 3:1), r.t., 2 h; d) Py, r.t., 3–5 h.

To improve the potency and selectivity of FBX‐1, chemical optimization was conducted. We separated FBX‐1 structure into three molecular fragments: the phenyl ring attached to the sulfonamide nitrogen (A moiety), the phenyl ring ortho to the pyridine nitrogen (B moiety), and the central pyridine core (C moiety), and the optimization efforts primarily targeted the A and B moieties (**Figure** **3**). The commercially available 3‐bromo‐2‐pyridinamine was coupled with benzyl mercaptan via a metal‐catalyzed coupling reaction to generate the intermediate **4**. This compound was then further reacted with iodobenzenes bearing various substituents to generate the intermediates **6‐1** ∼ **6‐4** (**Scheme** **1**). These intermediates were subsequently oxidized and chlorinated in the presence of N‐chlorosuccinimide (NCS) to yield sulfonyl chlorides **7‐1** ∼ **7‐4**. Finally, the sulfonyl chlorides were condensed with various aromatic amines to obtain a series of derivatives.

Subsequently, the inhibitory activities of these derivatives at 10 × 10^−6^ and 1 × 10^−6^ m were evaluated, and the structure–activity relationship (SAR) was analyzed (Figure S2, Supporting Information). In the first round of SAR, we briefly examined the influence of the substituents’ positions (C3 and C4), the electronic effects, and the polarity in the A moiety. We synthesized compounds **1a‐1l** and evaluated their enzymatic activities (Table S1, Supporting Information). Compared to the electronic effects, the polarity and the positions of the substituents had a more significant impact on the activity. In particular, 3‐CN (**1e**) and 3‐COOH (**1i**) enhanced the inhibitory activity. We speculate that these two groups may act as hydrogen bond acceptors, forming hydrogen bonds with FabX and enhancing the interaction. We then retained the 3‐CN group in the A moiety and optimized the B moiety to investigate the 2,4‐disubstitution by synthesizing **8a‐8c** (Figure 3). Notably, compound **8c** with the 2,4‐2Cl group showed improved inhibitory activity with an *IC*_50_ of 0.625 ± 0.082 × 10^−6^ µm (**Table** **1**).

**Table 1:** Bioactivity of selected compounds in FabX‐FabI enzyme‐coupled assay.

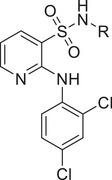					
Cmpd	R	*IC*_50_ [µm]	Cmpd	R	*IC*_50_ [µm]
**8c**	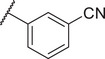	0.625 ± 0.082	**8m**	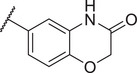	0.249 ± 0.038
**8j**	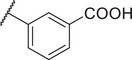	0.437 ± 0.042	**8n**	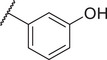	0.945 ± 0.111
**8k**	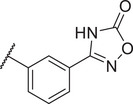	0.665 ± 0.085	**8p**	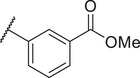	0.158 ± 0.030
**8l**	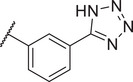	0.904 ± 0.177	**8q**	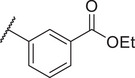	0.552 ± 0.118

Next, we retained the 2,4‐2Cl group in the B moiety and conducted a second round of the SAR study on the A moiety, including: (1) the introduction of multiple substituents (**8d‐8f**); (2) the replacement of the CN group with COOH (**8j**) or its bioisosteres (**8k, 8l**),^[^ ^20^ ^]^ or other hydrogen bond acceptor groups (**8n‐8o**); and (3) the replacement of the phenyl ring with other aromatic heterocyclic rings or benzofused rings (**8g‐8i, 8** **m**), leading to the synthesis of compounds **8d‐8o** (Schemes 1 **and** **2**). The intermediate **7‐4** was reacted with 3‐((tert‐butyldimethylsilyl)oxy)aniline to yield compound **10**, which was subsequently hydrolyzed to produce **8n**. Compound **8n** was then acetylated to form **8o**. We evaluated their FabX inhibitory activities and found that the introduction of 3‐COOH or its bioisosteres (**8j‐8l**) and 3‐OH (**8n**) maintained the inhibitory activity. Notably, replacing the phenyl ring with benzoxazinone (8m) enhanced activity (*IC*_50_ = 0.249 ± 0.038 × 10^−6^ m), while other modifications failed to improve the inhibitory activity (Table 1). We further conducted nitrogen atom positional scanning on the pyridine moiety (C moiety) or replaced it with benzene, and found that all were unfavorable for activity, except when the nitrogen was positioned para to the sulfonamide group (Table S2, Supporting Information). Moreover, inverting the sulfonamide linker or replacing it with an amide resulted in a complete loss of activity (data not shown).

**Scheme 2:**
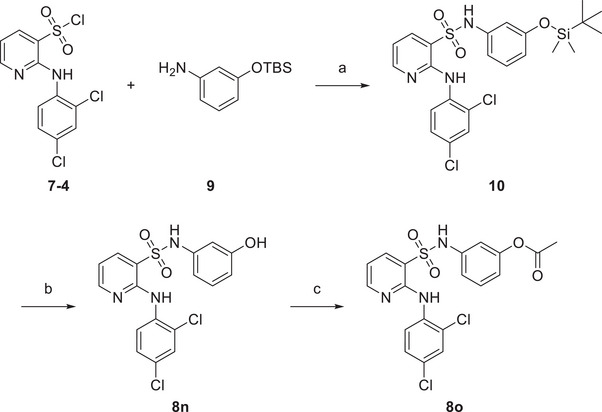
Synthetic route of compounds **8n, 8o** *^a^*. *^a^* Reagents and conditions: a) Py, r.t., 3–5 h; b) 4 m HCl in 1,4‐dioxane, r.t.; c) acetic anhydride, K_2_CO_3_, DMF, 0 °C to r.t.

**Scheme 3:**
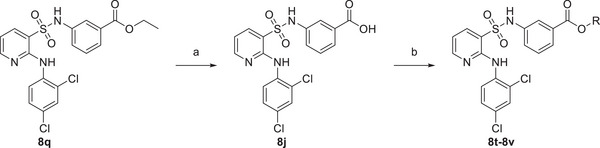
Synthetic route of compounds **8j, 8t‐8v** *^a^*. *^a^* Reagents and conditions: a) 5 m NaOH, EtOH, r.t.; b) ROH, DMAP, DCC, Et_3_N, DCM, 0 °C to r.t.

We evaluated the antibacterial activities of selected compounds (*IC*_50_ < 1 × 10^−6^ m) against several *H. pylori* strains (Table S3∖, Supporting Information), particularly against strains BHKS551 and BHKS211 (the FabX overexpressed strain, hereinafter referred to as FabX^OE^) to validate the on‐target effect in vivo. Note that FabX is overproduced 3.32‐fold in strain BHKS211 compared to the control strain BHKS210 carrying the empty vector (Figure S3, Supporting Information). However, none of these compounds demonstrated on‐target effect. Notably, compound **8j** (annotated as FBX‐1872, hereinafter) exhibited potent FabX inhibition but only low antibacterial activity with a minimum inhibitory concentration (MIC) value higher than 64 µg mL^−1^. This could be due to the fact that the carboxyl group of **8j** readily deprotonates and acquires negative charge at physiological pH, which reduces its lipophilicity and prevents it from permeating the bacterial cell membrane. To address this issue, we employed a prodrug strategy and synthesized esters from **8j** by attaching it to lipophilic alcohols (**8p‐8u**) or a lipophilic cation 6‐hydroxyhexyltriphenylphosphonium bromide (**8v**) (Figure 3). Similar strategies involving the use of prodrugs with cleavable ester linkages have been shown to facilitate drug delivery in bacteria.^[^ ^21^, ^22^ ^]^ Compounds **8p‐8s** were synthesized as shown in Scheme 1, while compounds **8t‐8v** were synthesized as described in **Scheme** **3**. **8q** was hydrolyzed with NaOH to yield **8j**, which was subsequently reacted with various alcohols by Steglich esterification to produce compounds **8t‐8v**.

**Figure 4:**
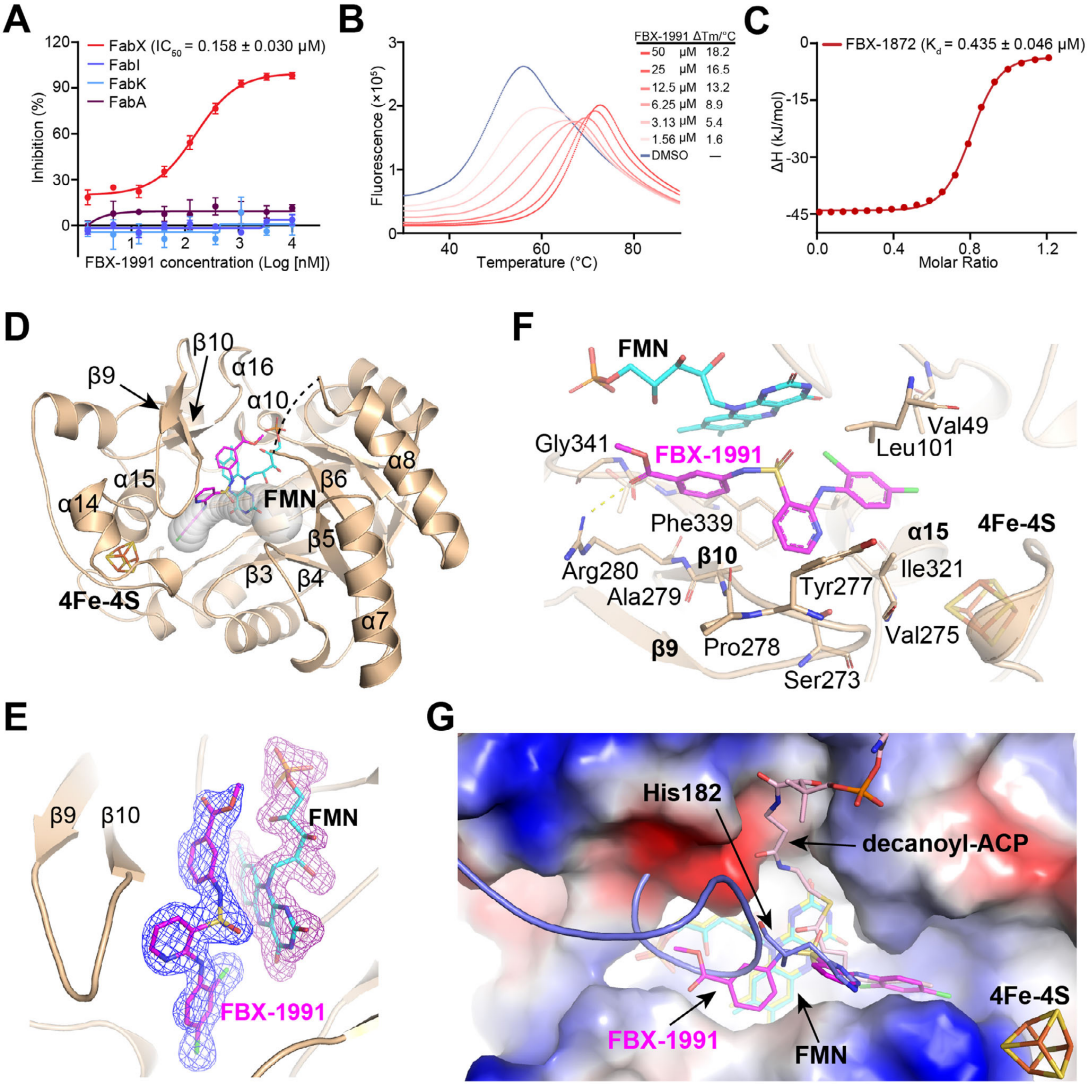
Identification of the highly potent FabX inhibitor FBX‐1991. A) Evaluation of the inhibitory activity of FBX‐1991 against FabX, FabI, FabK, and FabA. B) Dose‐dependent protein thermal shift assay of FBX‐1991 against FabX. C) Binding affinity evaluation of FBX‐1872 against FabX by using ITC. D) Overall crystal structure of FabX–FBX‐1991 complex. E) The fofc omit map contoured at 3.0 σ around FBX‐1991 and FMN. F) Interactions between FBX‐1991 and FabX residues. G) Superposition of the structures of FabX–FBX‐1991 complex with that of FabX–octanoyl‐ACP complex (colored in slate/yellow/light pink, PDB code: 7E1S).

Subsequently, we examined the enzymatic activities of these compounds and found that **8p** (annotated as FBX‐1991, hereinafter) exhibited an outstanding inhibitory activity against FabX with an *IC*_50_ value of 0.158 ± 0.030 × 10^−6^ m in vitro, and increased the stability of FabX by ≈18.2 °C (ΔTm) at a concentration of 50 × 10^−6^ m (**Figure** **4**A,B and Table 1). In contrast, it does not inhibit the enzymatic activities of the structurally conserved homolog FabK, the functionally conserved homolog FabI and the conventional UFA synthase FabA at compound concentrations of up to 10 × 10^−6^ m compound concentration, indicating a high selectivity of FBX‐1991 against FabX. Given that FBX‐1991 exhibits moderate solubility, we employed its highly similar analog, compound **8j** (FBX‐1872), for further evaluation of binding affinity evaluation against FabX. FBX‐1872 exhibits comparable inhibitory activity against FabX as FBX‐1991 (*IC*_50_: 0.437 ± 0.042 × 10^−6^ m) and increases the stability of FabX by ≈20.8 °C (ΔTm) at a concentration of 50 × 10^−6^ m (Figure S4A,B, Supporting Information). As expected, FBX‐1872 binds to FabX with a *K*_d_ value of 0.435 ± 0.046 × 10^−6^ m (Figure 4C and Figure S4C,D, Supporting Information). Further in vivo antibacterial assays suggested that the MICs of compounds **8p‐8s** were two‐ to eightfold higher for strain BHKS211 than for the control strain (**Table** **2**). Specifically, FBX‐1991 showed the best antibacterial activity among the compounds, and the MIC of FBX‐1991 against strain BHKS211 is 8‐fold higher than that of the control strain (128 vs 16 µg mL^−1^). In contrast, compounds **8t** and **8u** exhibited only low antibacterial activity, possibly due to their inability to be efficiently cleaved, preventing the release of the active compound **8j**. These results indicate that FBX‐1991 is a highly potent and selective FabX inhibitor in vitro and in vivo.

**Table 2:** Enzymatic and antimicrobial activities of ester derivatives.

Cmpd	FabX enzymatic activity			Antimicrobial activity/MIC [µg mL^−1^]					
	10 × 10^−6^ m [%]	1 × 10^−6^ m [%]	*IC*_50_ [× 10^−6^ m]	Hp129	G27	BHKS 568	BHKS 551	BHKS 210	BHKS 211
**8j**	100	65	0.437 ± 0.042	>64	>64	>64	>64	>128	>128
**8p**	98	83	0.158 ± 0.030	16	16	16	32	16	128
**8q**	100	60	0.552 ± 0.118	16	16	16	32	32	128
**8r**	37	N.A.	N.D.	32	64	32	64	32	64
**8s**	10	N.A.	N.D.	32	64	32	64	32	64
**8t**	25	N.A.	N.D.	>128	>128	>128	>128	>128	>128
**8u**	23	23	N.D.	>128	>128	>128	>128	>128	>128
**8v**	42	N.A.	N.D.	N.D.	32	16	16	16	32

Note: N.A. = No activity; N.D. = Not determined; *IC*_50_ = mean ± s.d., *n* = 3

### 2.4 Crystal Structure of FabX in Complex with FBX‐1991

To elucidate the inhibition mechanism of FBX‐1991 against FabX, we determined the X‐ray crystal structure of FabX in complex with FBX‐1991 in space group P2_1_ at a resolution of 2.00 Å resolution (Table S4, Supporting Information). Subsequent structural analysis indicated that FBX‐1991 binds in the region between the FMN cofactor and the catalytic tunnel of FabX surrounded by α10, α14‐α16, β3‐β6, β9, and β10, and inserts its 2,4‐dichlorophenyl group into the catalytic tunnel, pointing towards the bottom of the tunnel near the [4Fe‐4S] cluster (Figure 4D,E and Figure S5, Supporting Information). Superposition of FabX−FBX‐1991 complex structure with previously determined FabX (PDB code: 7E1Q) or FabX–octanoyl‐ACP (PDB code: 7E1S) structures suggested that the binding of FBX‐1991 to FabX does not alter the overall structure of FabX, according to the RMSD values of 0.267 and 0.339, respectively, with the exception that the key catalytic loop between β6 and α8, which contains the family conserved and essential catalytic residue His182, was disordered in the structure of FabX–FBX‐1991 complex (Figure S5, Supporting Information).^[^ ^19^ ^]^ In addition, we also determined the crystal structure of FabX in complex with FBX‐1872 in space group P2_1_ at a resolution of 1.60 Å (Table S4, Supporting Information). The structural comparison suggested that the structures of FabX–FBX‐1991 and FabX–FBX‐1872 complexes are highly similar with an RMSD value of 1.919 and both inhibitors adopt exactly the same conformations inside the catalytic tunnel of FabX, indicating similar inhibitory mechanisms of these inhibitors (Figure S6, Supporting Information).

In the structure of FabX–FBX‐1991 complex, FBX‐1991 is stabilized in the catalytic tunnel predominantly through hydrophobic interactions (Figure 4F and Figure S7, Supporting Information). The methyl benzoate of FBX‐1991 (A moiety) interacts with the isoalloxazine ring of FMN via π–π interactions. Meanwhile, the carboxyl group of the methyl benzoate group forms an H‐bond with the sidechain of Arg280 located at the N‐terminus of FabX β10 strand, which further stabilizes the methyl benzoate in the correct position. The superposition of the structure of FabX–FBX‐1991 complex with that of FabX–octanoyl‐ACP complex suggest that the methyl benzoate group of FBX‐1991 introduces direct stereospecific blockades with the key loop between β6 and α8, disrupting the regular conformation of the loop for catalysis (Figure 4G). In addition, the central pyridine core of FBX‐1991 predominantly interacts with the sidechains of hydrophobic residues located on the β9 and β10 strands including Ser273, Val275, Tyr277, Pro278, and Ala279, as well as the sidechains of Ile321 from α15 and Phe339 (Figure S8A, Supporting Information).

In addition to the interactions mentioned above, the 2,4‐dichlorophenyl ring ortho to the pyridine nitrogen of FBX‐1991 (B moiety) inserts into the bottom of the catalytic tunnel, pointing toward the [4Fe‐4S] cluster. The benzene ring of FBX‐1991 forms hydrophobic interactions with the sidechains of Val275 from the loop between β9 and β10, and Ile321 and Leu325 from α15 (Figure S8B, Supporting Information). Moreover, the chloro group at the C4 position in the B moiety of FBX‐1991 is stacked by hydrophobic interactions among the sidechains of Val304 and Cys305 from the [4Fe‐4S] cluster domain, Ala322 from α15, and Val49. The superposition of the structure of FabX–FBX‐1991 complex with that of *Porphyromonas gingivalis* FabK (PDB code: 4IQL) indicates that the [4Fe‐4S] cluster domain contributes substantially to the binding of FBX‐1991 to FabX. The absence of the [4Fe‐4S] cluster domain in FabK results in FBX‐1991 not binding to FabK (Figure S9, Supporting Information).^[^ ^23^ ^]^ Remarkably, the chloro group at the C2 position in the B moiety of FBX‐1991 is right stacked in a hydrophobic cavity formed by the atoms of the isoalloxazine ring of FMN, the backbone of Gly25, and the sidechains of Leu325 and Val49 (Figure S8B, Supporting Information). Further superposition of the structures of FabX–FBX‐1991 complex with that of FabX–octanoyl‐ACP complex indicates that the 2,4‐dichlorophenyl ring of FBX‐1991 blocks the insertion of the octanoyl substrates into the catalytic tunnel of FabX and abolishes the enzymatic activity of FabX in catalysis of dehydrogenation and isomerization of acyl substrate carried by ACP (Figure 4G). These structural observations and comparisons suggest that FBX‐1991 binds inside the catalytic tunnel of FabX, and inhibits the catalytic activity of FabX by disrupting the regular conformation of the key loop which tilts the essential catalytic residue His182 and prevents the insertion of the acyl substrate carried by ACP.

### 2.5 FBX‐1991 Inhibits *H. pylori* Growth and ROS Excretion by Specifically Targeting FabX

**Figure 5:**
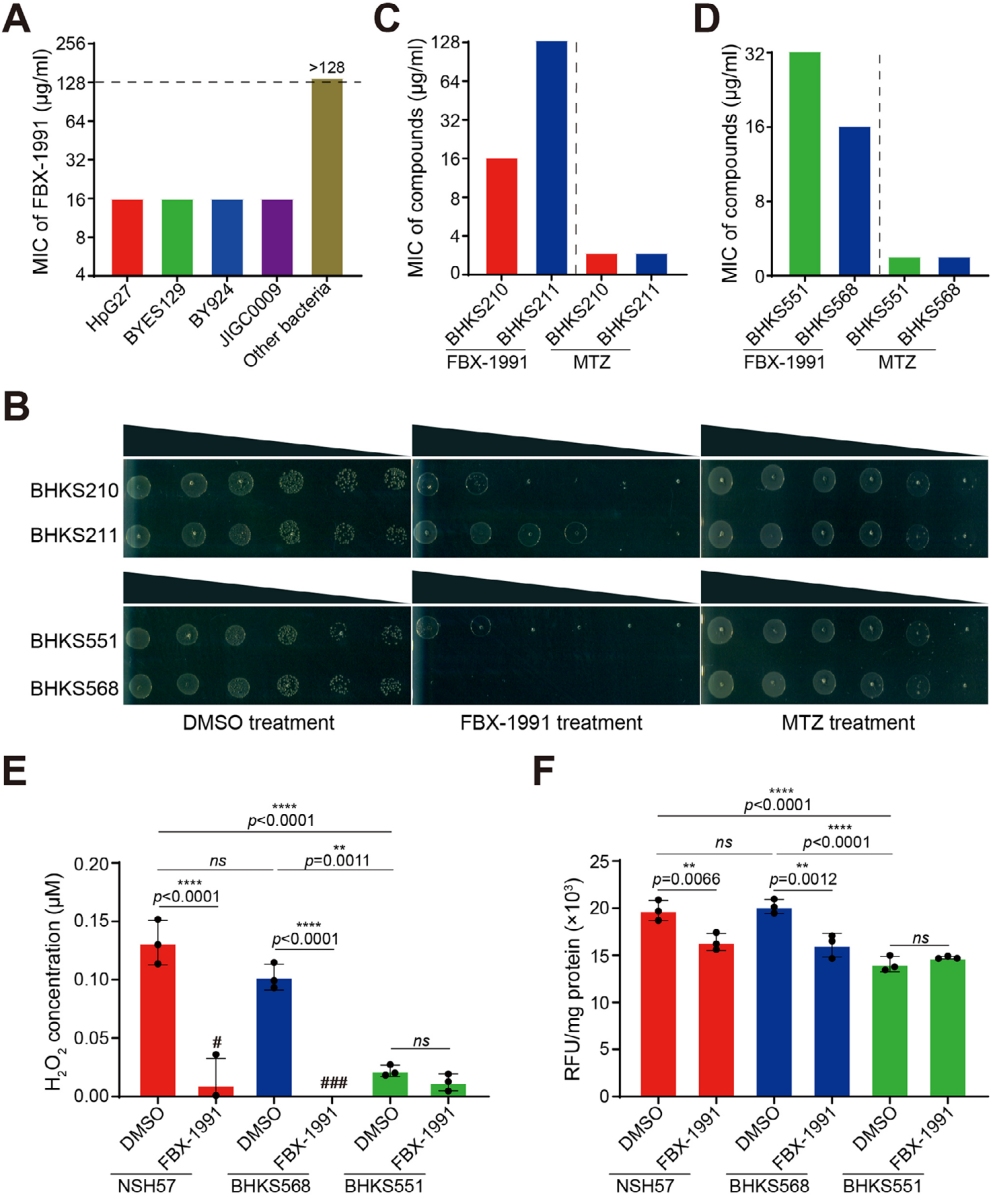
Identification of FBX‐1991 as a FabX specific inhibitor in vivo. A) Antibacterial activity of FBX‐1991 against *H. pylori* standard strain HpG27 and three clinical isolates. The MIC for *H. pylori* strains is 16 µg mL^−1^, whereas for the other 15 bacterial strains (as detailed in the Experimental Section), all the MIC values are >128 µg mL^−1^. B) Resistant spot dilution assays were performed on Columbia blood plates with FBX‐1991, comparing strain BHKS210 (NSH57/empty vector) with BHKS211 (NSH57/*fabX^OE^*), as well as strain BHKS551 (NSH57 IR0203::*fabA* Δ*fabX*) with BHKS568 (NSH57 IR0203::*fabX* Δ*fabX*). Growth on plates with metronidazole (MTZ) or DMSO served as negative controls. The data are representative of three independent experiments. C) Overexpression of *fabX* conferred an increased MIC of FBX‐1991. D) Replacement of the *fabX* gene with *fabA* conferred an increased MIC of FBX‐1991. The antibacterial activities of FBX‐1991 against strains BHKS210, BHKS211, BHKS551, and BHKS568 were evaluated through MIC determination. E) H_2_O_2_ release by *H. pylori* cells was quantified following treatment with DMSO or 1×MIC of FBX‐1991 for 4 h. ###, not detected. F) ROS production was quantified using a Cell ROS Assay kit in *H. pylori* cells treated with DMSO or 1×MIC of FBX‐1991 for 4 h. The data represent the mean (±SD) of three independent experiments with statistical analyses performed by one‐way ANOVA with Tukey's multiple comparisons test.

To further investigate the inhibitory mechanism of FBX‐1991 in vivo, its antibacterial activity was tested against a panel of 15 different bacterial species. The results suggested that FBX‐1991 significantly inhibits the growth of *H. pylori* strain G27 and three clinical drug‐resistant isolates with MIC values of 16 µg mL^−1^, but does not inhibit the growth of the non‐*Helicobacter* strains, even at a concentration of 128 µg mL^−1^ (**Figure** **5**A). We then examined the effect of FabX overexpression on the sensitivity of *H. pylori* to FBX‐1991. The spot dilution assays showed that the overexpression of FabX conferred increased resistance to FBX‐1991 (Figure 5B). This increased resistance was further validated by an eightfold elevation in MIC values in the FabX overexpressing strain compared to the parental strain carrying the empty vector (Figure 5C). To further confirm FabX as the specific target of FBX‐1991 in vivo, we utilized strains BHKS568 (NSH57 IR0203::*fabX* Δ*fabX*) and BHKS551 (NSH57 IR0203::*fabA* Δ*fabX*) to compare their sensitivity to FBX‐1991. As expected, strain BHKS568 exhibited greater sensitivity to FBX‐1991 compared to strain BHKS551 (Figure 5D). As a control, the overexpression of FabX and the replacement of FabX with FabA did not affect the sensitivity of *H. pylori* to metronidazole (MTZ). These results suggested that FBX‐1991 inhibits the growth of *H. pylori* by specifically targeting FabX in vivo.

Subsequently, we evaluated the in vivo UFA and cyclopropane fatty acid synthesis levels as well as the levels of hydrogen peroxide and ROS excretion of *H. pylori* strains after treatment with FBX‐1991. The GC‐MS analysis of fatty acid composition suggested that FBX‐1991 treatment partially inhibits the biosynthesis of UFA and cyclopropane fatty acids in *H. pylori* in vivo, as expected (Figure S10, Supporting Information). In addition, FBX‐1991 significantly reduced the accumulation of hydrogen peroxide and overall ROS levels in NSH57 and BHKS568 strains compared to DMSO treatments (Figure 5E,F). In contrast, the BHKS551 strain, in which *fabX* was replaced by *fabA*, showed no change in its levels upon FBX‐1991 treatment. These results indicate that FBX‐1991 partially inhibits UFA and cyclopropane fatty acid synthesis, ROS production and subsequent excretion from *H. pylori* by specifically targeting FabX in vivo.

## 3 Discussion

*H. pylori* infection leads to numerous human gastric diseases such as gastric fundus inflammation, peptic ulcers, and gastric adenocarcinomas. Current broad‐spectrum antibiotic based quadruple chemotherapy against *H. pylori* infection induces several intolerable side effects including antibiotic resistance, gastrointestinal symptoms, and dysbiosis. Developing highly selective pharmaceuticals against *H. pylori* is an urgent global issue. So far, previous studies have shown that numerous natural products from fruits, vegetables, spices, and medicinal plants possess inhibitory effects against *H. pylori*, with MIC values reaching up to micrograms per milliliter (µg/mL), likely by targeting its urease, adhesive, invasive, and inflammatory properties.^[^ ^24^ ^]^ Besides, bacteria utilize a conventional and essential Type‐II fatty acid biosynthesis pathway (FAS‐II) to produce SFA and UFA to support the cell growth, and therefore the enzymes involved in FAS‐II have been considered important  antibacterial  drug targets. Remarkably, we previously discovered that *H. pylori* uses an atypical dehydrogenase/isomerase FabX instead of the species conserved dehydratase/isomerase FabA to synthesize UFA from saturated fatty acid substrates, accompanied by ROS production. Moreover, since genes that encode FabX and its homologous enzymes rarely exist in the genomes of human associated bacteria, and knocking out the *fabX* gene is lethal to *H. pylori*, targeting FabX with inhibitors could be a more specific anti‐*H. pylori* therapeutic strategy without significantly disrupting the healthy gut microbiota, and may facilitate the development of species‐specific anti‐*H. pylori* chemotherapy.

In this study, we demonstrated that the ROS produced by FabX is essential for *H. pylori* gastric colonization in addition to synthesizing UFA for *H. pylori* growth. This well explains why *H. pylori* chose to utilize the atypical species‐specific FabX rather than other conventional enzymes (such as FabA) to catalyze such a high‐energy demanding desaturation reaction for UFA production. Moreover, we developed a lead inhibitor FBX‐1991 targeting FabX specifically, which inhibits the enzymatic activity of FabX by disrupting the regular conformation of the catalytic loop and preventing the insertion of the acyl substrate from catalysis, showing a comparable MIC value to that of the reported natural products.^[^ ^24^ ^]^ In addition to inhibiting the growth of *H. pylori*, FBX‐1991 further inhibits ROS generation of *H. pylori* for its gastric colonization, and such primary in vivo effects of FBX‐1991 are likely accompanied by and due to its partial inhibition of UFA synthesis. In contrast, a potent FabX inhibitor would be expected to completely inhibit bacterial growth, as observed with the FabA inhibitor 3‐decynoyl‐N‐acetylcysteamine.^[^ ^36^ ^]^ In conclusion, the mechanism of *H. pylori* in the corrosion of gastric mucosa that we elucidated, as well as the inhibitor we reported may help to better understand the pathogenic function of *H. pylori* in gastric infection, and provide the molecular basis for the development of species‐specific anti*‐H. pylori* chemotherapy.

## 4 Experimental Section

### Bacterial Strains and Culture Media

*H. pylori* strains used in this study were cultured on brain heart infusion (BHI) broth (Oxoid, UK) or Columbia blood agar medium (Oxoid, UK) supplemented with 10% fetal calf serum (FCS). The cultures were incubated at 37 °C for 48–72 h under microaerobic environments in a double‐gas CO_2_ incubator (Binder, Germany). The wild‐type strains G27 and NSH57 used in this study were kindly provided by Prof. Nina R. Salama from the Fred Hutchinson Cancer Research Center (USA). Three clinical *H. pylori* strains (BYES129, BY924, and JIGC0009) were isolated from biopsy samples from three patients with gastritis or gastric cancer using standard protocols. Strains BHKS551 (NSH57 IR0203::fabA ΔfabX) and BHKS568 (NSH57 IR0203::fabX ΔfabX) were described previously.^[^ ^19^ ^]^ The following 15 reference strains of different species were also used: *Escherichia coli* ATCC 25922, *Klebsiella pneumoniae* ATCC 35657, *Pseudomonas aeruginosa* PAO1, *Salmonella enterica* serovar Typhimurium ATCC 14028, *Shigella dysenteriae* Sd197, *Acinetobacter baumannii* ATCC 19606, *Proteus mirabilis* ATCC 29906, *Enterobacter cloacae* ATCC 13047, *Stenotrophomonas maltophilia* ATCC 51331, *Bacillus subtilis* 168, *Enterococcus faecium* ATCC 19434, *Staphylococcus aureus* ATCC 25923, *Staphylococcus haemolyticus* ATCC 29970, *Listeria monocytogenes* EGDe, and *Morganella morganii* ATCC 25830. These non‐*Helicobacter* bacterial strains were cultured as described previously.^[^ ^25^ ^]^

### DNA Manipulation and Construction of *H. pylori* Strain Overexpressing fabX

The *fabX*‐overexpressing strain BHKS211 (NSH57/*fabX^OE^*) was constructed by introducing the expression plasmid pBHK683, an *E. coli*‐*H. pylori* shuttle plasmid pTM117 that expresses *fabX*,^[^ ^26^ ^]^ into the wild‐type strain NSH57 using natural transformation. To construct the expression plasmid, oligonucleotide primers (*fabX*‐XbaI‐L: CCTGTCTAGACCTTAAATCCTTAGTTT, *fabX*‐KpnI‐R: ATATGGTACCTTAACCCTCTGTAAGCTC) were first designed, synthesized by Sangon Biotech Co., Ltd. (Shanghai). Genomic DNA from the *H. pylori* strain NSH57 was used as a template to amplify the target gene *fabX* via PCR. The recombinant plasmid pBHK683 was then constructed by ligating the digested DNA fragment with the digested pTM117 vector using restriction endonucleases KpnI, XbaI, along with T4 DNA ligase. The construct was verified by colony PCR, DNA sequencing. The control strain BHKS210 (NSH57/vector) was constructed by introducing the empty vector pTM117 into the wild‐type strain NSH57.

### RNA Extraction, Quantitative Real‐Time PCR

Total RNA of two *H. pylori* strains (BHKS210, BHKS211) was extracted using the RNAprep Pure Cell/Bacteria Kit (Tiangen Biotech; Beijing, China) according to the manufacturer's instructions. The extracted RNA was confirmed to be free of genomic DNA by PCR, was subsequently used for reverse transcription reactions. The first‐strand complementary DNA (cDNA) was synthesized using HiScript II Reverse Transcriptase Kit (Vazyme Biotech; Nanjing, China). The resulting cDNA served as the template for subsequent PCR amplification. qRT‐PCR was performed using SupRealQ Purple Universal SYBR qPCR Master Mix (Vazyme Biotech) on a real‐time PCR instrument (LightCycler 96 System; Roche, Switzerland). The reactions were conducted according to the manufacturer's instructions, with the following thermal cycling conditions: preincubation at 95 °C for 30 s, followed by 40 cycles of amplification at 95 °C for 10 s, 60 °C for 30 s. The relative expression of the *fabX* gene was normalized using the housekeeping 16S rRNA gene, quantified using the comparative 2^−ΔΔCt^ method. The PCR primer sequences are as follows: 16S‐F: 5′‐CTTAACCATAGAACTGCATTTGAAACTAC‐3′, 16S‐R: 5′‐GGTCGCCTTCGCAATGAGTA‐3′, *fabX*‐F: 5′‐AGCGAGTGGGGTGCAGATGG‐3′, *fabX*‐R: 5′‐GCCCTAGCCGGATAGCCTACAG‐3′. The bar graph was generated using GraphPad Prism 9.5 software, statistical analysis was performed using Student's *t*‐test.

### Antibiotic Sensitivity Assay

The sensitivity of *H. pylori* strains to various compounds was assessed by determining the MIC and evaluating the growth of the strains on the drug‐containing agar media. The MIC was measured using the broth microdilution assay as described previously.^[^ ^27^ ^]^ Twofold serial dilutions of the test compounds were prepared using BHI broth supplemented with 10% FCS in 96‐well microtiter plates. Diluted *H. pylori* cultures, adjusted to a final concentration of 5 × 10^5^ CFU mL^−1^, were then added to each well. The cultures were incubated for 48–72 h in a microaerobic environment at 37 °C. The MIC was determined by visual inspection.

The drug sensitivity of *H. pylori* strains was also determined by observing their growth on drug‐containing Columbia blood agar media. *H. pylori* strains were initially cultured to the mid‐logarithmic growth phase in BHI broth supplemented with 10% FCS. The bacterial concentration was then adjusted to an optical density of 0.5 at 600 nm and concentrated 25‐fold. A fivefold concentration gradient was used to prepare serial dilutions of the bacterial suspension. Subsequently, 3 µL of the diluted bacterial suspensions were spotted onto pre‐prepared drug‐containing agar media. The culture plates were incubated in a microaerobic environment for 3–4 d and were subsequently scanned for analysis.

### Mouse Colonization Assay

The in vivo protocol design and procedures were approved by the Institutional Animal Care and Use Committee (IACUC) of Nanjing Medical University under ethical protocol approval no. IACUC‐2005036. Six‐week‐old specific‐pathogen‐free female C57BL/6 mice, obtained from the Animal Core Facility of Nanjing Medical University of China, were utilized in this study. The mice were randomly assigned to three groups, each consisting of six individuals. *H. pylori* strains NSH57, BHKS551, and BHKS568 were cultured in BHI broth containing 10% FCS for ≈24 h. These bacterial cultures were then resuspended in the culture medium to an optical density of 3.0 at 600 nm (1 × 10^9^ CFU mL^−1^). A 0.4 mL bacterial suspension was administered to the C57BL/6 mice via oral gavage every 48 h, with a total of five administrations. Two weeks following the final gavage, the mice were euthanized, and their stomachs were excised, weighed, and homogenized in BHI broth containing 10% FCS. Serial dilutions of the gastric homogenate were plated onto Columbia blood agar supplemented with Dent selective supplement (Oxoid, UK) and incubated in a microaerobic environment at 37 °C for ≈5 d. *H. pylori* colonies were then counted, and the results were expressed as CFU per gram of stomach tissue.

### Preparation of Cellular Fatty Acid Methyl Esters and Analysis of Fatty Acids

The *H. pylori* cultures were grown at 37 °C in 10 mL BHI broth containing FCS until reaching an optical density of 0.4 at 600 nm. The cellular fatty acid methyl esters were then methylated and extracted according to previously described protocols with minor modifications.^[^ ^28^, ^29^ ^]^ Briefly, *H. pylori* whole cells from above were washed with PBS, resuspended in 1 mL of 4N NaOH‐methanol, and incubated at 100 °C for ≈1 h with shaking. Subsequently, 2 mL of 4 m HCl‐methanol was added, and fatty acid methyl esters were extracted with petroleum ether. The petroleum ether phases were dried, and the fatty acid methyl esters were resuspended in n‐hexane and analyzed by gas chromatography‐mass spectrometry (GC‐MS) as described previously.^[^ ^30^ ^]^ The fatty acid methyl esters were identified by comparing their retention times to known standards and by matching their mass spectra with reference spectrum libraries NIST08 and WILEY8n.

### Measurement of ROS and Hydrogen Peroxide Production

The ROS levels were quantitatively measured using a Cell (GMS10016.13 v. A) ROS Assay kit (Genmed Scientifics, Inc., Wilmington, DE) which uses 5‐ (and‐6)‐chloromethyl‐2′,7′‐dichlorofluorescin diacetate (CM‐H_2_DCFDA) as a fluorescent probe. This probe detects various primary ROS, including O_2_^• −^, H_2_O_2_, OH^•^, nitrogen dioxide. CM‐H_2_DCFDA freely permeates the plasma membrane, is hydrolyzed by intracellular esterase in the cytosol, forming nonfluorescent 2′,7′‐dichlorodihydrofluorescein (DCFH). In the presence of ROS, DCFH is oxidized to highly fluorescent dichlorofluorescein (DCF). Briefly, the *H. pylori* cultures were grown at 37 °C in BHI broth containing 10% FCS until reaching an optical density of 0.4 at 600 nm. Aliquots of the GENMED Reagent B were added to the cultures (100 µL) to achieve a final concentration of 10 × 10^−6^ m. The cultures were then incubated at 37 °C for another 30 min in the dark. The fluorescence intensity was subsequently measured on a Cell Imaging Multimode Microplate Reader (Cytation 5; Biotek, USA) using excitation, emission wavelengths of 495 and 520 nm, respectively. The H_2_O_2_ levels produced by *H. pylori* strains were determined using the Amplex^®^ Red Hydrogen Peroxide/Peroxidase Assay Kit from Invitrogen, following the previously described method.^[^ ^19^ ^]^ To assess the effect of compound 1991 on H_2_O_2_, ROS generation in vivo, *H. pylori* cells were incubated with compound 1991 at 1×MIC or DMSO for 4 h at 37 °C, followed by measurement as described above.

### Protein Expression and Purification

The subcloning, expression, and purification of FabX, FabI, and ACP from *H. pylori*, FabK from *S. pneumoniae*, and FabA from *E. coli* were described previously.^[^ ^19^ ^]^ Briefly, *fabX*, *fabI*, *fabK*, and *fabA* genes were subcloned to pQE2 vector, while the *Acp* gene was subcloned to pET32a vector, respectively. The proteins were expressed in BL21(DE3) Rosetta cells, purified by using Ni‐NTA affinity column, HiTrap‐SP column, GE HiLoad 16/600 Superdex75 prep grade gel‐filtration column. The peaks from the gel‐filtration column were further pooled, concentrated, and the final concentration of the proteins was quantified by the Bradford reagent purchased from Bio‐Rad.

### FabX‐FabI Coupled Enzymatic Characterization and High‐Throughput Screening

The reaction mixture contains 50 × 10^−3^ m sodium phosphate, pH 7.2, 50 × 10^−3^ m NaCl, 0.25 × 10^−6^ m FabX, 1 × 10^−6^ m FabI, 125 × 10^−6^ m NADH, and 4 × 10^−6^ m decanoyl‐ACP in a total volume of 25 µL. A final concentration of 50 × 10^−6^ m compound from the chemical library (5% DMSO) was added to the reaction, and then reaction was then measured by monitoring the decrease in absorbance at 340 nm (NADH) using a UV Synergy Mx (Biotek) at 37 °C. The obtained data were fitted using nonlinear regression analysis of GraphPad Prism 8 software.

### Chemical Synthesis

The details of the chemical synthesis are described in the Supporting Information.

### Protein Thermal Shift Assay

The protein thermal shift assay was performed by using a QuantStudio7 PRO (Thermo Fisher). FabX was diluted with a storage buffer to a final concentration of 5 × 10^−6^ m and mixed with various concentrations of compounds and 5X SYPRO Orange dye (Sigma‐Aldrich). After incubation, 10 µL of samples were added to a 384‐well plate and centrifuged. The thermal shift program was set to 25–95 °C for 30 min. Subsequently, the melt curve was analyzed by protein thermal shift analysis software (Thermo Fisher) and the Tm value was fitted.

### Microscale Thermophoresis Assay

The assay was performed using a NanoTemper Monolith NT.115 instrument (NanoTemper Technologies).^[^ ^31^ ^]^ 10 × 10^−6^ m of FabX was labeled with the fluorescence dye NT‐647 according to manufacturer's protocol. 10 µL labeled protein was subsequently mixed with various concentrations of compound and incubated at room temperature for 5 min. For measurements, samples were filled into capillaries and the assay was conducted on the NanoTemper Monolith NT.115 instrument with 20% excitation power and 40% MST power. Statistical analysis as *K*_d_ was performed using NanoTemper analysis software and the figures were presented using GraphPad Prism software.

### Isothermal Titration Calorimetry (ITC) Assay

FabX protein was prepared in a dialysis buffer containing 20 × 10^−3^ m NaH_2_PO_4_, pH 8.0, 50 × 10^−3^ m NaCl and 1 × 10^−3^ m TCEP. MicroCal PEAQ‐ITC (Malvern) was used to determine the binding affinities between FabX (in the cell) and compound (in the syringe) with a molar ratio of 1:6.25 (FabX: compound) at 10 °C. The titration assay was conducted using a default method of 19 injections at an interval of 180 s. The *K*_d_ and *N* values were fitted by MicroCal PEAQ‐ITC Analysis Software (Malvern).

### Crystallization

Purified 30 mg mL^−1^ FabX was mixed with 1 × 10^−3^ m FBX‐1991 or FBX‐1872 compound (5% DMSO), and incubated at 4 °C for 30 min. 1 µL of the mixture was then combined with an equal volume of reservoir solution and equilibrated against 100 µL of the reservoir solution at 293 K. Brown color crystals were observed within a week in the reservoir solution containing 0.1 m Tris‐HCl, pH 7.0, and 25% PEG3350 (w/v). The crystals were soaked in protectant containing mother liquor with 30% glycerol (v/v) and flash frozen for X‐ray diffraction.

### Data Collection and Refinement

The crystal diffraction data were collected at BL19U1 beamline of National Facility for Protein Science in Shanghai (NFPS) at Shanghai Synchrotron Radiation Facility (SSRF), and processed with HKL3000 v721.3 (Table S4, Supporting Information).^[^ ^32^ ^]^ Molecular replacement was used to solve the structures with the reported FabX structure (PDB code: 7E1Q) as the search model by using CCP4 8.0‐Phaser.^[^ ^19^, ^33^ ^]^ The solution was then conducted by the graphics program Coot 0.9.8.1,^[^ ^34^ ^]^ and refined by using Phenix 1.19.2.^[^ ^35^ ^]^ The protein structure viewer was performed by PyMol 2.5.4. The atomic coordinates and structure factors for the crystal structures reported in this study were deposited under accession codes 9JSY (FabX−FBX‐1991 complex) and 9K7H (FabX−FBX‐1872 complex).

### Chemical Synthesis

All reagents and solvents were purchased from commercial sources and were used without further purification. The column chromatography purification instrument used was CombiFlash Rf 150 (Teledyne Isco) flash chromatograph. NMR data were measured by Varian Mercury Plus 400 and Bruker Ascend 600 MHz NMR spectrometers, with tetramethylsilane (TMS) as the internal standard, and the spectra were processed by MestRenova 12.0. Chemical shifts are expressed in ppm. In the NMR tabulation, s indicates singlet; d, doublet; t, triplet; q, quartet; p, pentet; hept, heptet; and m, multiplet. Low‐resolution mass spectrometry (LRMS) data were measured by a 6120 quadrupole mass spectrometer (Agilent, Santa Clara, CA), while high‐resolution mass spectrometry (HRMS) data were measured by a three‐stage TOF 5600+ MS/MS system (AB Sciex, Concord, ON, Canada) with electrospray ionization (ESI) as the ionization mode. The purity of all derivatives was determined by high‐performance liquid chromatography (HPLC, Agilent 1260 Infinity II system or Shimadzu LC‐20AD) using a C18 column (Promosil, 150 × 4.6 mm, 5 µm, UV 280 nm). The mobile phases are as follows: A, H_2_O (with 0.1% TFA); B, methanol (with 0.1% TFA). The flow rate is 1.0 mL min^−1^. The gradient program was set as follows: (method 1) 0–2 min, 40% B; 2–8 min, linear increase to 95% B; 8–11 min, 95% B; 11–13 min, return to 40% B; 13–15 min, 40% B for column re‐equilibration; (method 2) 0–2 min, 40% B; 2–8 min, linear increase to 95% B; 8–16 min, 95% B; 16–18 min, return to 40% B; 18–20 min, 40% B for column re‐equilibration. The purities of all final compounds were determined to be ≥95% by HPLC.

### Statistical Analysis

All statistical analyses were performed using the GraphPad Prism (GraphPad Software, La Jolla, CA) software package. Data were presented as the means ± SD of three independent experiments with statistical analyses conducted by one‐way ANOVA with Tukey's multiple comparisons test.

## Conflict of Interest

L.Z., X.R., L.Z., L.Z., H.B., and X.H. were named inventors of a pending patent application (Application No. 202510035387.2; applicant: Fudan University; filed on January 9, 2025, to the Chinese Patent Office) related to the work. The other authors declare no competing interests

## Author Contributions

L.Z., X.R., and X.H. contributed equally to this work. L.Z., H.B., and L.Z. conceived the original idea, designed the experiments; L.Z. and C.C. performed protein purification; L.Z. conducted enzymatic assay, biophysical assay, and crystallization; X.H., D.H., G.Z., and L.Z. performed antibacterial assays; X.R. carried out chemical synthesis; L.Z., H.B., and L.Z. wrote the paper. All authors discussed and commented on the manuscript.

## Supporting information



Supporting Information

## Data Availability

The data are available in the paper and its Supporting Information files. The diffraction data generated in this study have been deposited in the PDB database (www.rcsb.org) under accession codes: 9JSY (FabX–FBX‐1991 complex) and 9K7H (FabX‐FBX‐1872 complex) (Table S4, Supporting Information).
