# Supplementary material for: Antagonist Targeting the Species‐Specific Fatty Acid Dehydrogenase/Isomerase FabX for Anti‐H. pylori Infection
Source: Adv Sci (Weinh). 2025 Mar 16;12(18):2414844. doi: 10.1002/advs.202414844 (PMC12079444; doi:10.1002/advs.202414844)
Supplement: Supplementary file 1 — Supporting Information [file ADVS-12-2414844-s001.docx]

Supplementary Information for

Antagonist Targeting the Species-Specific Fatty Acid Dehydrogenase/Isomerase FabX for Anti-*H. pylori* Infection

Lin Zhang^1,§^, Xiaoxue Ruan^2,§^, Xudong Hang^3,4,§^, Ding Heng^3,5^, Chang Cai^1^, Liping Zeng^3^, Guoxin Zhang^5^, Lu Zhou^2,6,*^, Hongkai Bi^3,4,*^, Liang Zhang^1,7,*^

^1^Department of Pharmacology and Chemical Biology, School of Medicine, Shanghai Jiao Tong University, Shanghai, 200025, China; ^2^Department of Medicinal Chemistry, School of Pharmacy, Fudan University, Shanghai, 201203, China; ^3^Department of Pathogen Biology & Jiangsu Key Laboratory of Pathogen Biology, Nanjing Medical University, Nanjing, Jiangsu, 211166, China; ^4^NHC Key Laboratory of Tropical Disease Control, School of Tropical Medicine, Hainan Medical University, Haikou, Hainan, 571199, China; ^5^Department of Gastroenterology, The First Affiliated Hospital of Nanjing Medical University, Nanjing, Jiangsu, 210009, China; ^6^Quzhou Fudan Institute, Quzhou, 324002, China; ^7^Department of Chemical Biology, School of Chemistry and Chemical Engineering, Shanghai Jiao Tong University, Shanghai, 200240, China.

^§^ These authors contribute equally to this work.

*Correspondences: Liang Zhang, Email: [liangzhang2014@sjtu.edu.cn](mailto:liangzhang2014@sjtu.edu.cn); Hongkai Bi, Email: [hkbi@njmu.edu.cn](mailto:hkbi@njmu.edu.cn); Lu Zhou, E-mail: [zhoulu@fudan.edu.cn](mailto:zhoulu@fudan.edu.cn)

This PDF file includes:

Table S1 to S4;

Figures S1 to S10;

Schemes S1 to S3;

**Table S1. First round optimization of A moiety.**

| **Cmpd** | **R** | **Inhibition (%)** | | **Cmpd** | **R** | **Inhibition (%)** | |
| --- | --- | --- | --- | --- | --- | --- | --- |
|  |  | **10 μM** | **1 μM** |  |  | **10 μM** | **1 μM** |
| **1a** |  | N.A. | N.A.* | **1g** |  | 21 | N.A. |
| **1b** |  | N.A. | N.A. | **1h** |  | 35 | N.A. |
| **1c** |  | 23 | N.A. | **1i** |  | 65 | 19 |
| **1d** |  | 12 | N.A. | **1j** |  | 63 | N.A. |
| **1e** |  | 88 | 31 | **1k** |  | 12 | N.A. |
| **1f** |  | 23 | N.A. | **1l** |  | 25 | 12 |

*N.A.= No activity (Inhibition < 10%)

**Table S2. Investigation of pyridine** **moiety.**

| **Cmpd** | **R** | **Inhibition** | | **IC_50_ (μM)** |
| --- | --- | --- | --- | --- |
|  |  | **10 μM** | **1 μM** |  |
| **8c** | **** | 100 | 77 | 0.625±0.082 |
| **11a** | **** | 100 | 100 | 0.480±0.061 |
| **11b** | **** | N.A.* | N.A. | N.D.* |
| **11c** | **** | 60 | 17 | N.D. |
| **11d** | **** | 94 | 60 | 2.379±0.225 |

*N.A.= No activity; N.D.= Not determined; IC_50_ = mean ± s.d., n = 3.

**Table S3.** **Antimicrobial activity of selected compounds.**

| **Cmpd** | **MIC (μg/mL)** | | | | | |
| --- | --- | --- | --- | --- | --- | --- |
|  | **Hp129** | **G27** | **BHKS568** | **BHKS551** | **BHKS210** | **BHKS211** |
| **8c** | 16 | 16 | 16 | 16 | 16 | 32 |
| **8h** | N.D. | 64 | 64 | 64 | 64 | 64 |
| **8i** | 64 | 64 | 64 | 64 | 64 | 64 |
| **8j** | ＞64 | ＞64 | ＞64 | ＞64 | ＞128 | ＞128 |
| **8k** | ＞64 | ＞64 | ＞64 | ＞64 | N.D. | N.D. |
| **8l** | ＞64 | ＞64 | ＞64 | ＞64 | N.D. | N.D. |
| **8m** | 128 | 128 | 128 | 128 | 128 | 128 |
| **8n** | 16 | 16 | 16 | 16 | 16 | 16 |

**Table S4. Data collection and refinement statistics.**

|  | FabX−FBX-1991 | FabX−FBX-1872 |
| --- | --- | --- |
| **PDB codes** | 9JSY | 9K7H |
| **Data collection** | | |
| Space group | P2_1_ | P2_1_ |
| Cell dimensions |  |  |
| *a, b, c* (Å) | 52.98, 65.54, 53.28 | 47.18, 48.51, 75.90 |
| α, β, γ (°) | 90.00, 98.29, 90.00 | 90.00, 93.79, 90.00 |
| Wavelength (Å) | 0.9785 | 0.9785 |
| Resolution (Å)* | 50.00-2.00 (2.07-2.00) | 50.00-1.60 (1.69-1.60) |
| *R*_merge_ (%) | 10.8 (39.0) | 5.5 (36.7) |
| *I*/σ*I* | 13.8 (3.2) | 16.4 (2.6) |
| Completeness (%) | 98.3 (88.7) | 92.6 (72.3) |
| Redundancy | 6.0 (4.7) | 5.9 (4.2) |
| CC_1/2_ (%) | 98.2 (88.6) | 99.9 (86.5) |
| **Refinement** | | |
| Resolution (Å) | 26.36-2.00 | 30.32-1.60 |
| No. reflections | 22901 | 41302 |
| *R*_work_/*R*_free_ | 0.162/0.195 | 0.220/0.248 |
| No. atoms |  |  |
| Protein | 2677 | 2738 |
| Water | 260 | 220 |
| Ligand/ion | 69 | 67 |
| B-factors |  |  |
| Protein | 27.07 | 22.92 |
| Water | 35.21 | 29.49 |
| Ligand/ion | 24.41 | 21.05 |
| R.m.s deviations |  |  |
| Bond lengths (Å) | 0.009 | 0.008 |
| Bond angles (°) | 1.120 | 1.082 |

*Highest-resolution shell is shown in parentheses.


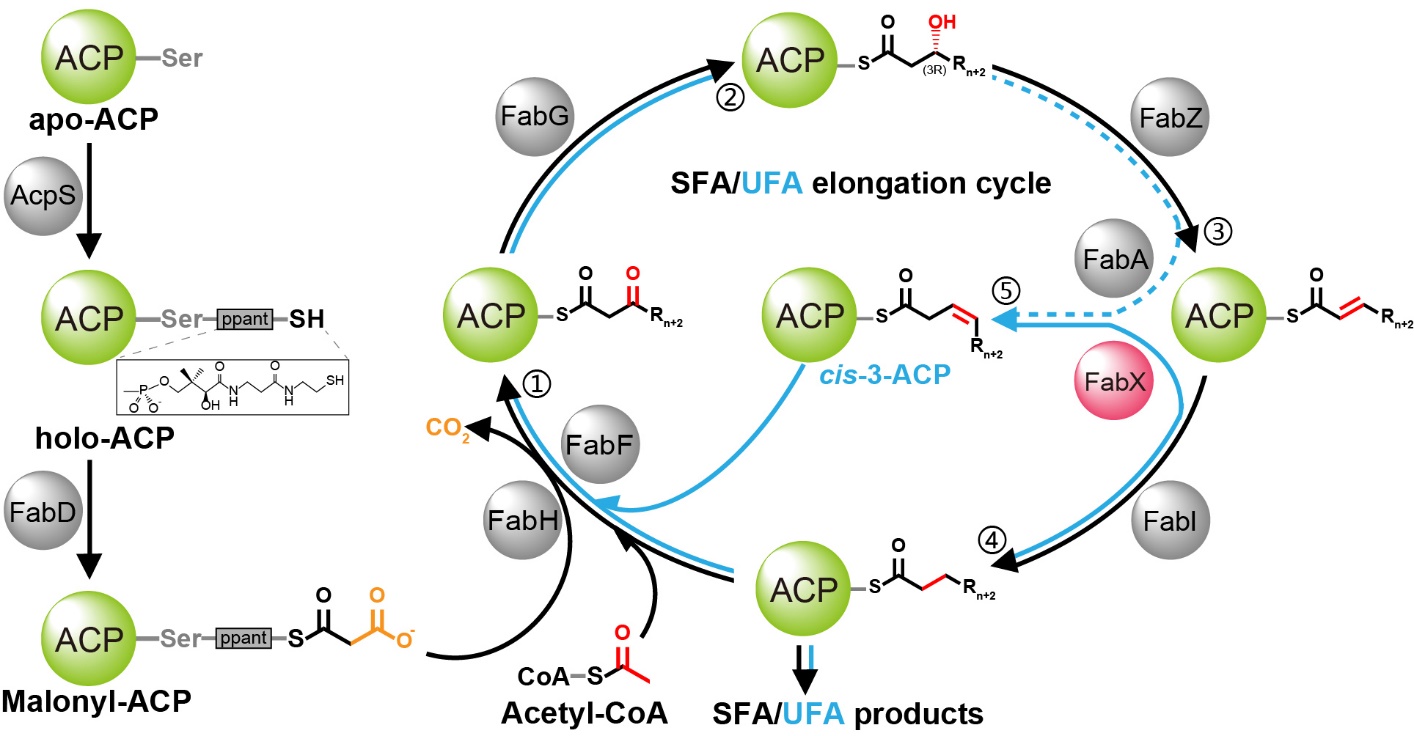


**Figure S1. Schematic diagram of Type-II fatty acid biosynthesis pathway (FAS-II) in *Helicobacter pylori*.** FabX (red sphere) catalyzes the dehydrogenation and isomerization of the saturated acyl group on ACP (the fifth step in the elongation cycle) for *cis*-double bond introduction and UFA synthesis. The black and blue curves indicate the SFA and UFA synthesis processes, respectively, while the blue dashes indicate the absence of the conventional *fabA* gene in the genome of *H. pylori*.


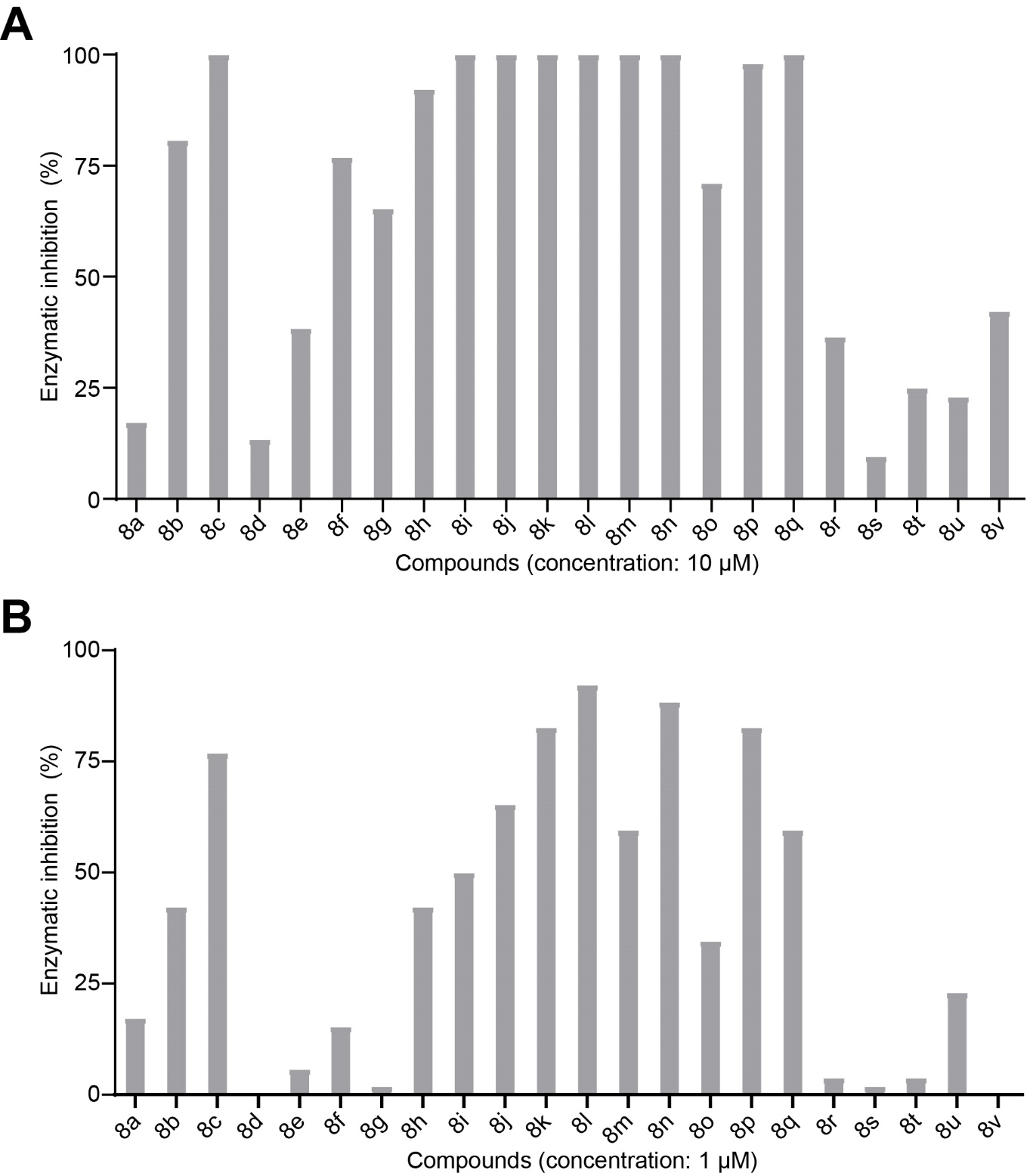
**Figure S2. Bioactivity evaluation of FBX-1 derivatives at 10 μM and 1 μM in the FabX-FabI enzyme-coupled assay.**


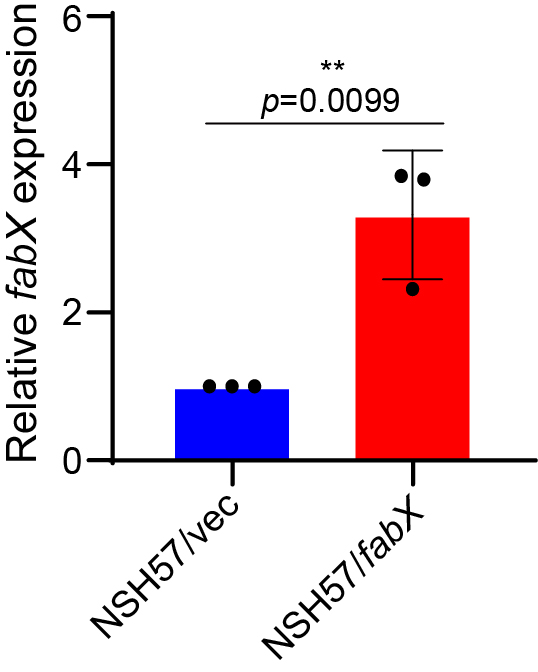
**Figure S3. Overexpression of FabX in strain BHKS211.** BHKS210 (NSH57/Vec) and BHKS211 (NSH57/*fabX*) were cultured in BHI broth supplemented with 10% FCS to an OD_600_ of 0.3 and then harvested. RNA isolation, reverse transcription, and real-time PCR were performed as described in the EXPERIMENTAL METHODS section. The relative levels of *fabX* expression in BHKS211 compared to the control strain BHKS210 are represented. The data are the averages ± standard deviations from three independent experiments.


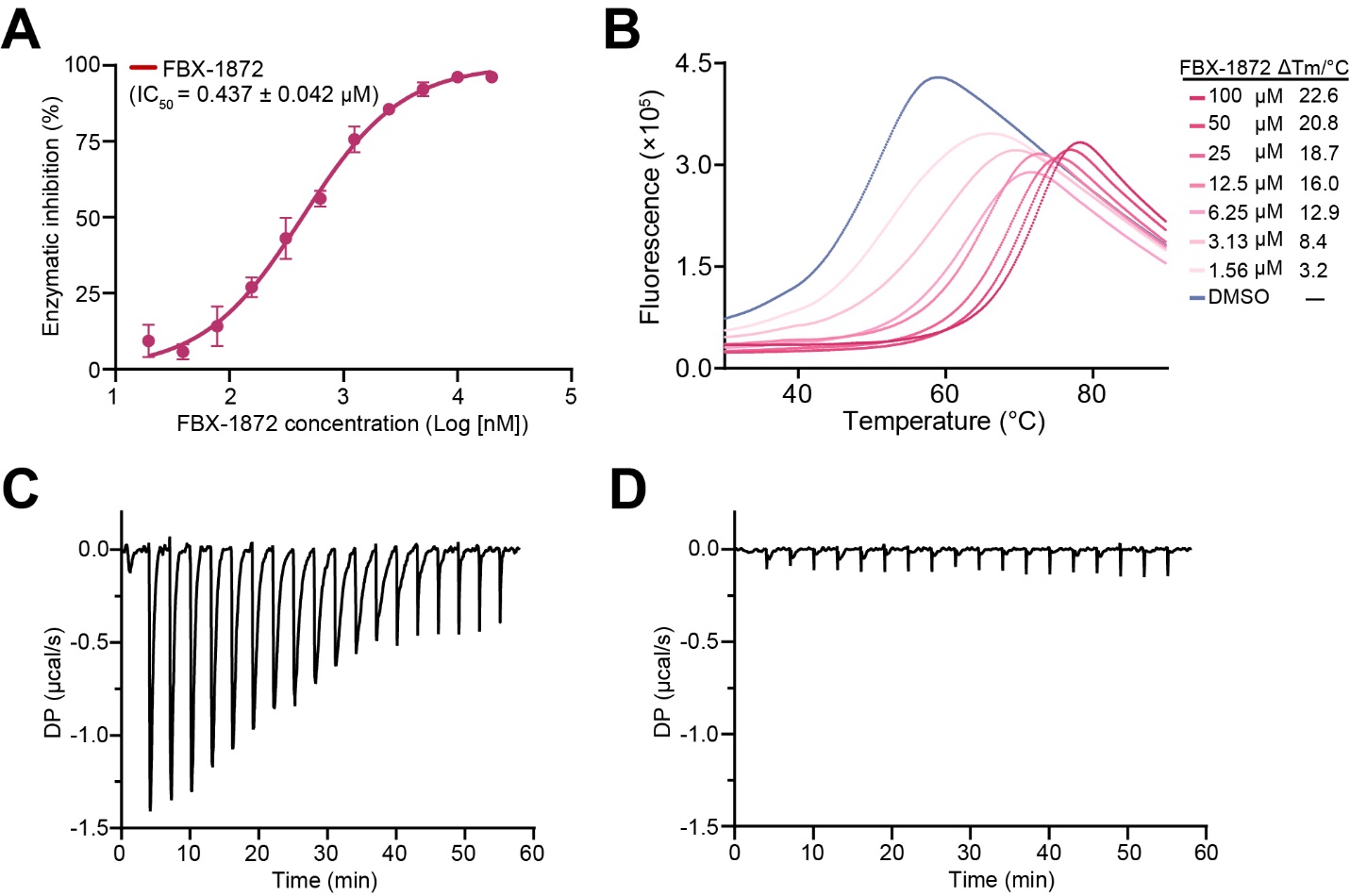
**Figure S4. Identification of FBX-1872 against FabX.** (A) Evaluation of enzymatic inhibition of FBX-1872 against FabX. (B) Dose dependent protein thermal shift assay of FBX-1872 against FabX. (C) ITC assay of FBX-1872 against FabX. (D) ITC assay of FBX-1872 against buffer solution.

**
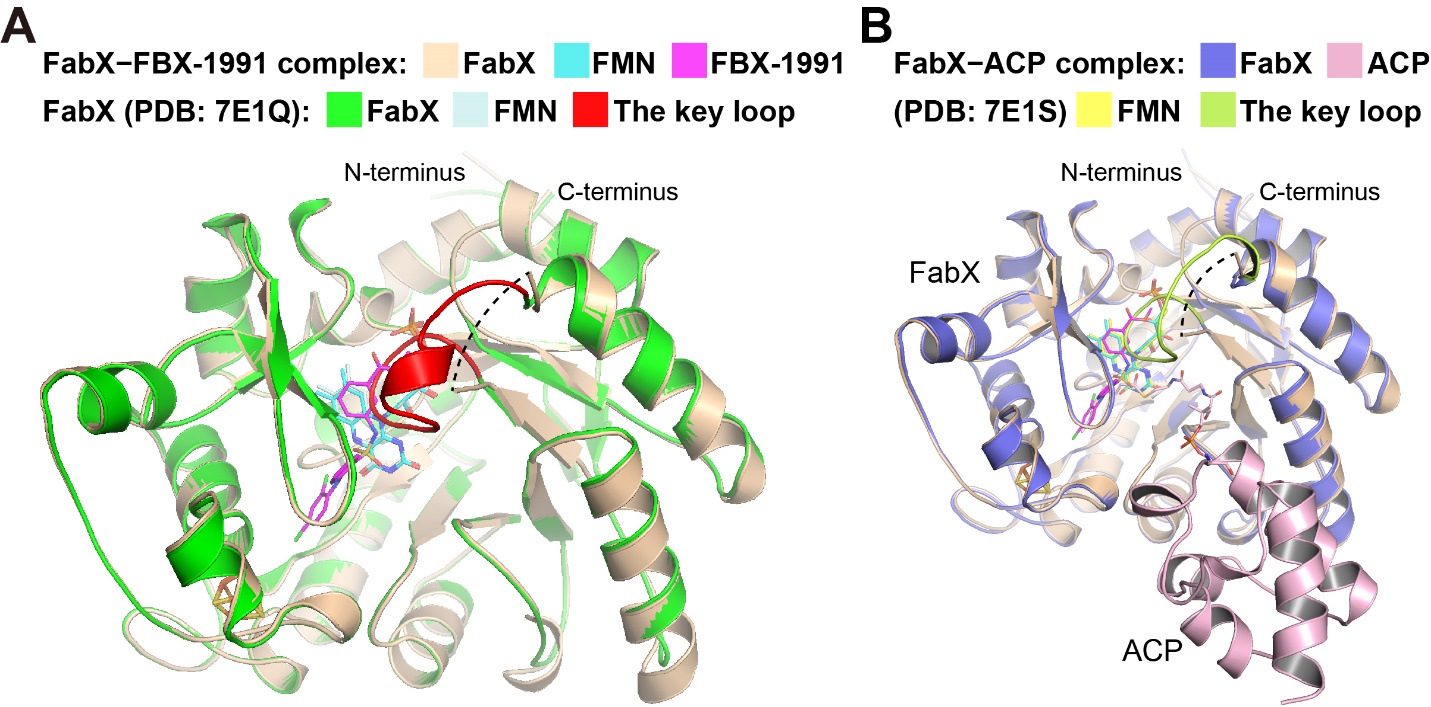
**

**Figure S5. Structural superposition of FabX−FBX-1991** complex structure **with FabX or FabX−octanoyl-ACP complex structures.** (A) Superposition of the FabX−FBX-1991 complex structure with that of FabX (PDB code: 7E1Q). The red region indicates the key loop between β6 and α8 observed in the FabX structure, which contains the family conserved and essential catalytic residue His182. In the structure of FabX−FBX-1991 complex, this loop was disordered. (B) Superposition of the FabX−FBX-1991 complex structure with that of FabX−octanoyl-ACP complex (PDB code: 7E1S). The lime region indicates the key loop observed in the FabX−octanoyl-ACP complex.


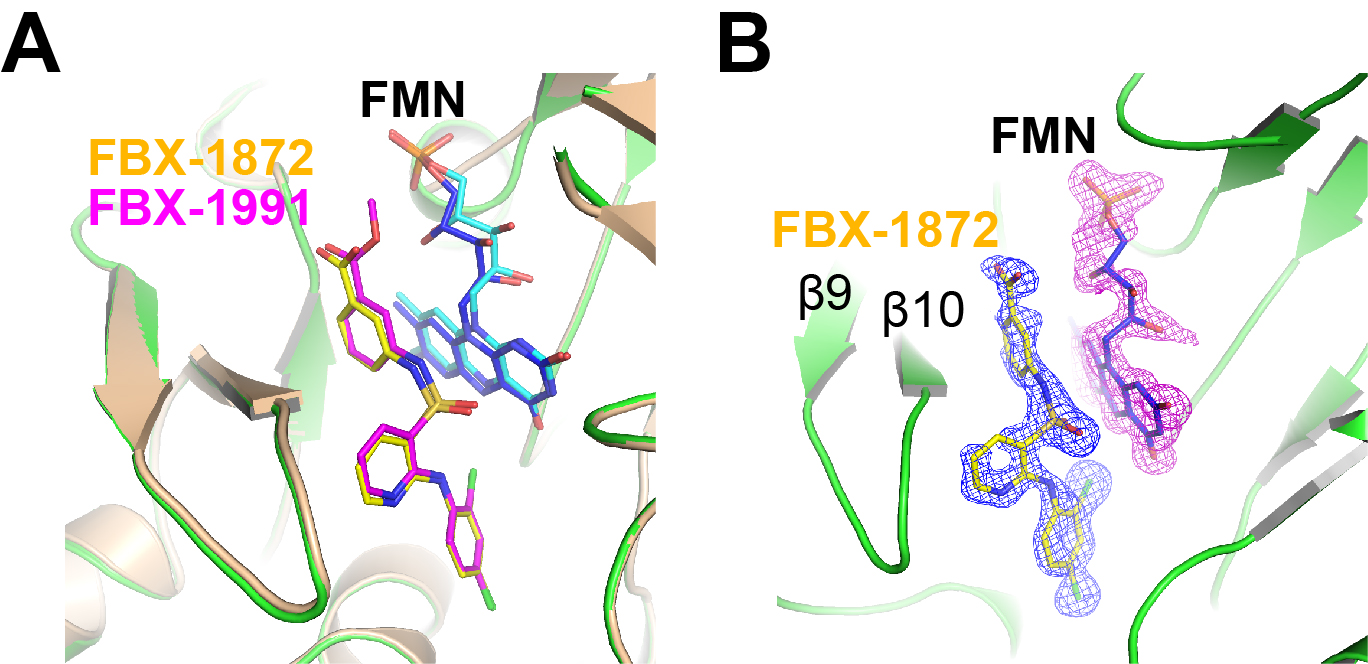
**Figure S6. Structural superposition of FabX−FBX-1991 and FabX−FBX-1872 complexes.** (A) The binding conformations of FBX-1991 and FBX-1872 inside the catalytic tunnel. (B) The fofc omit map contoured at 3.0 σ around FBX-1872 and FMN.

**
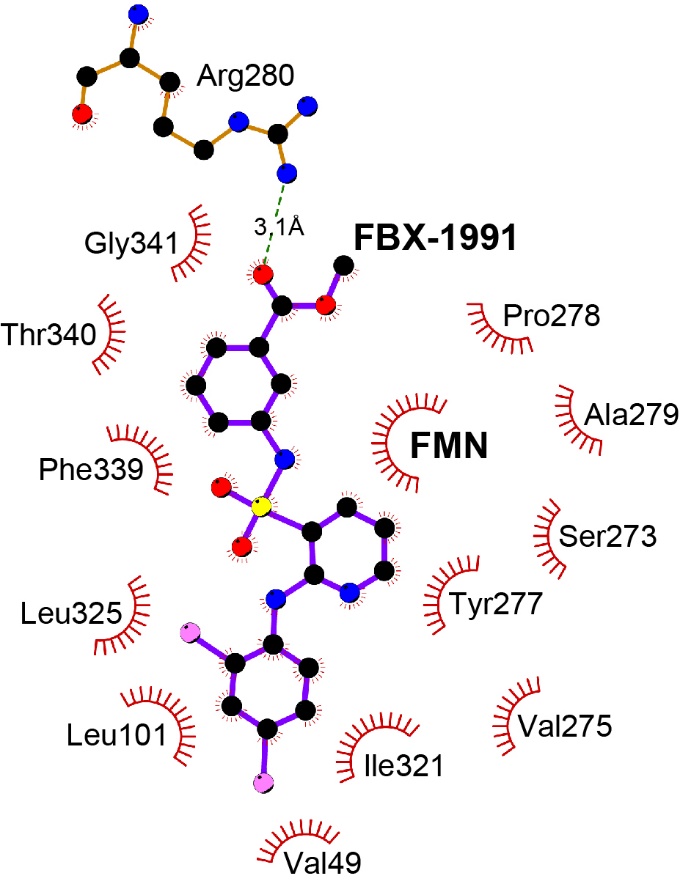
**

**Figure S7. Interactions between FabX and FBX-1991.** The schematic diagram was generated by using LigPlot^+^ v.2.2 online server (<https://www.ebi.ac.uk/thornton-srv/software/LigPlus/>), and modified.

**
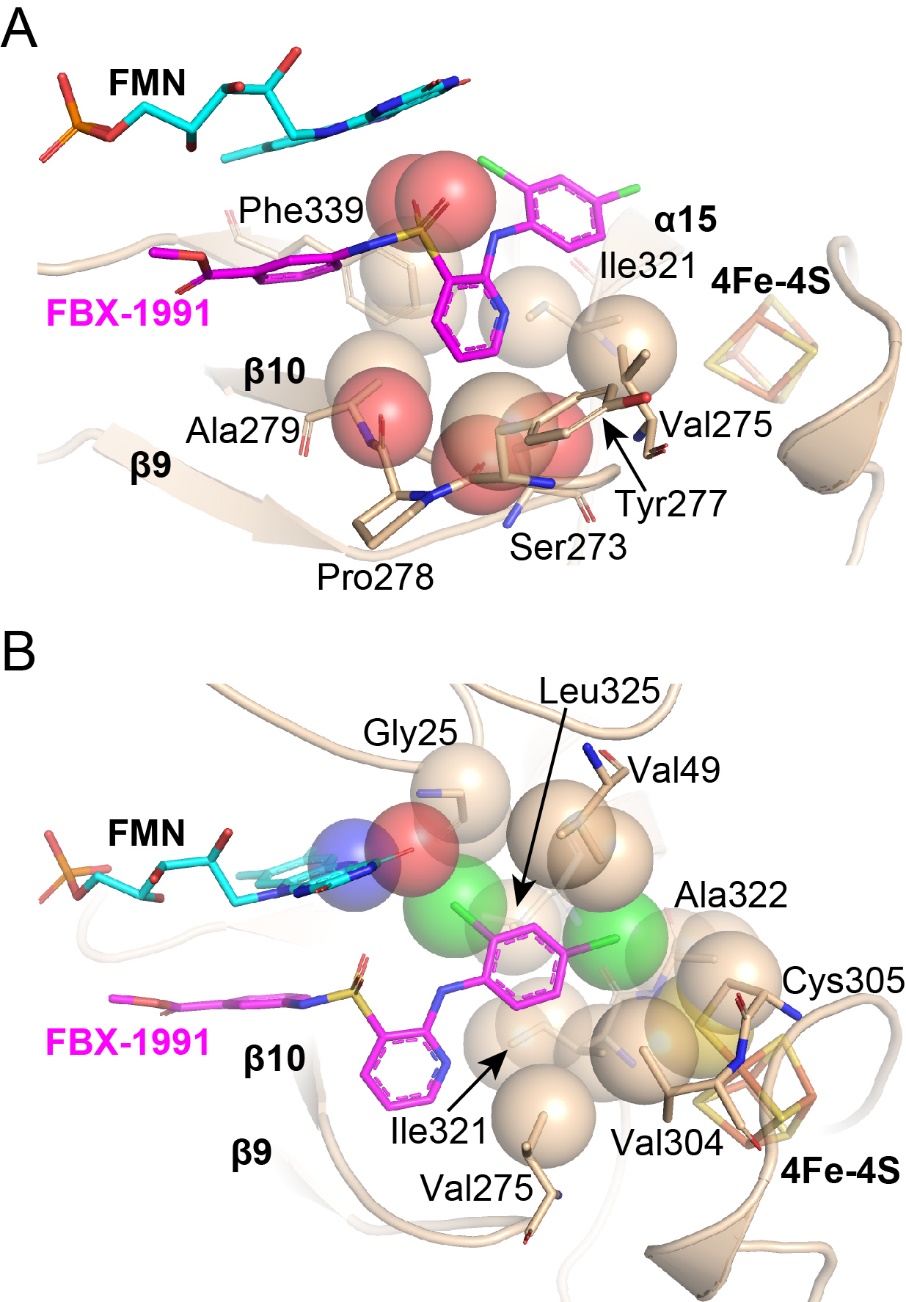
**

**Figure S8. Interactions between FabX and FBX-1991.** The spheres indicate the atoms involved in the interactions.


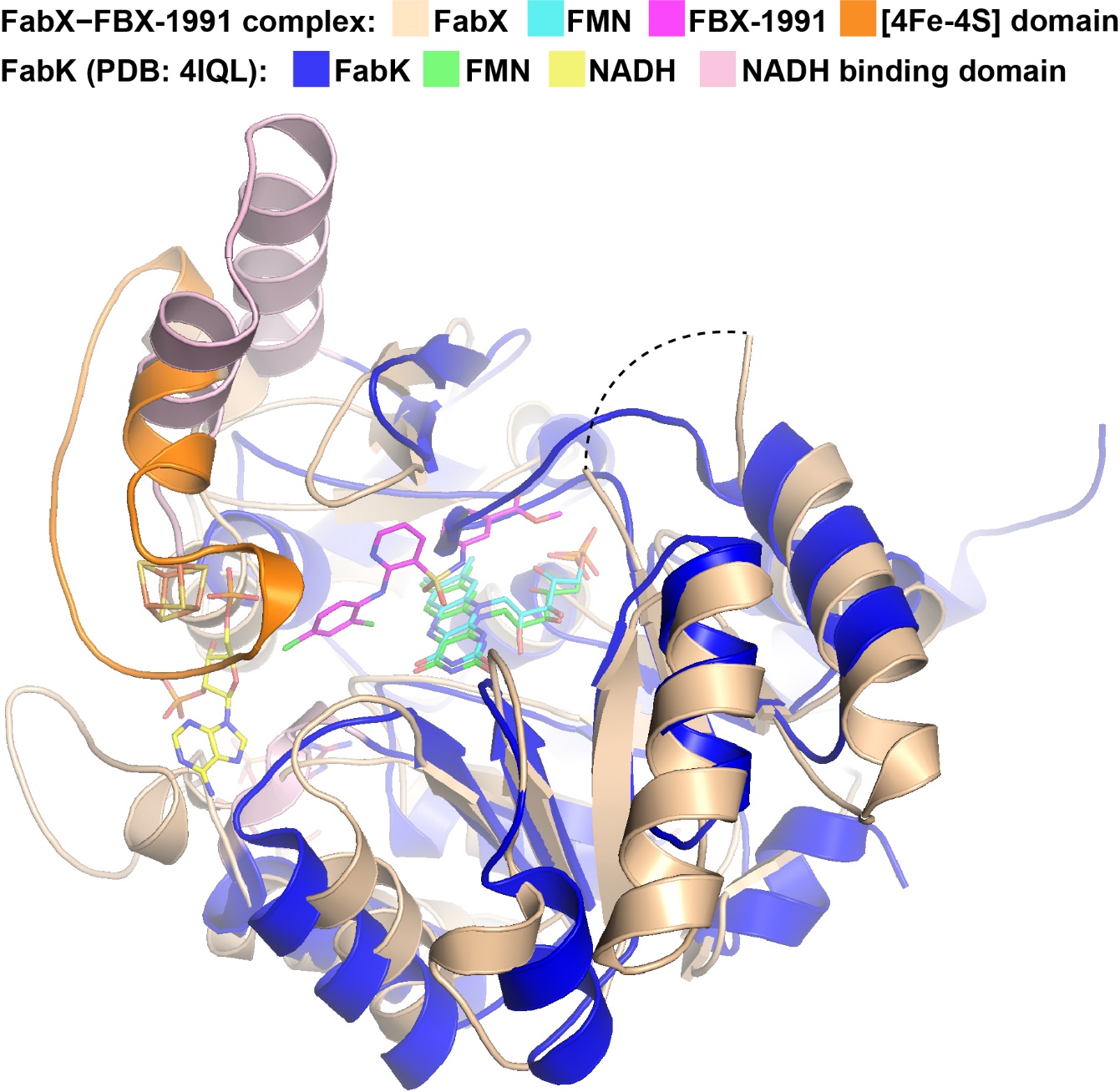


**Figure S9. Superposition of the FabX−FBX-1991 complex structure with the reported FabK structure from *Porphyromonas gingivalis* (PDB code: 4IQL).**

**
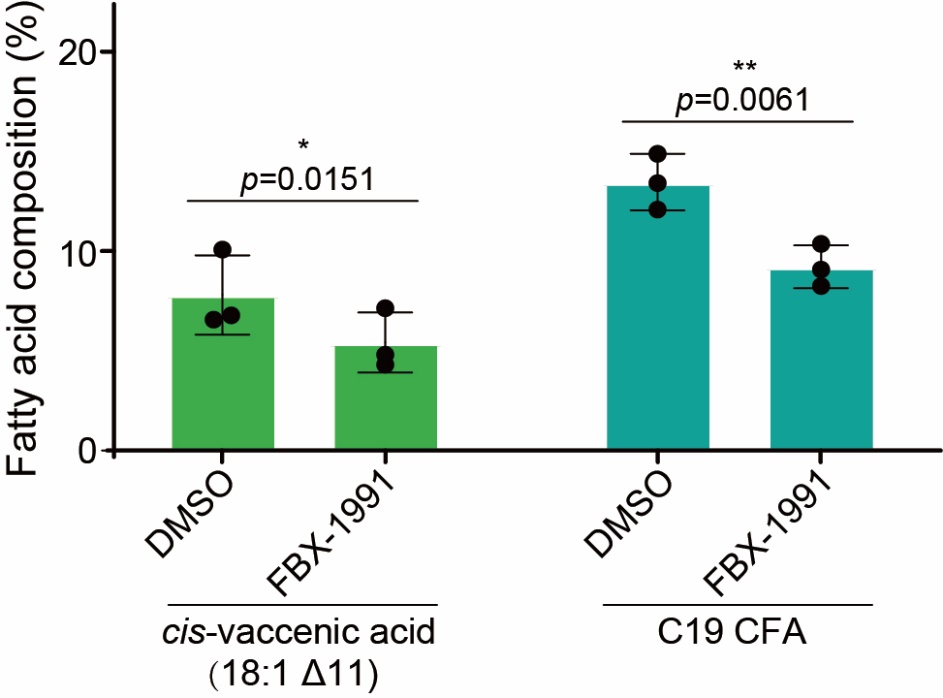
**

**Figure S10. Analysis of fatty acid composition of *H. pylori* strain NSH57 following treatment with DMSO or 4×MIC of FBX-1991 for 8 hours.** The data represent the mean (± SD) of three independent experiments with statistical analyses performed by one-way ANOVA with Tukey’s multiple comparisons test.

**Scheme S1. General Synthetic Route of Compounds FBX-1, 1a-1l, 8a-8i, 8k-8m, 8p-8s*^a^***

*^a^* Reagents and conditions: (a) Pd_2_(dba)_3_, Xantphos, toluene, 80 ℃, overnight; (b) Pd_2_(dba)_3_, Xantphos, Cs_2_CO_3_, 1,4-Dioxane, 100 ℃, overnight; (c) NCS, AcOH/H_2_O (V/V=3:1), r.t., 2 h; (d) Py, r.t., 3-5 h.

**3-(benzylthio)pyridin-2-amine (4)**

To a solution of 3-bromo-2-pyridinamine (5.62 g, 32 mmol) in toluene (100 mL), benzyl mercaptan (4.23 g, 34 mmol), Pd_2_(dba)_3_ (593 mg, 0.02 eq), Xantphos (750 mg, 0.04 eq), DIPEA (9.2 mL, 2.2 eq) were added at room temperature. The reaction mixture was stirred at 80℃ overnight under inert atmosphere. The resulting mixture was diluted with water, extracted with ethyl acetate (EtOAc). The extract was washed with saturated brine, dried over Na_2_SO_4_ and evaporated in vacuo. The residue was purified by silica gel chromatography to afford **4** (5.93 g, 85% yield) as yellow solid. MS (ESI) (*m/z*): 217.0 (M+H)^+^. ^1^H NMR (400 MHz, DMSO-*d*_6_) δ 7.86 (dd, *J* = 4.8, 1.6 Hz, 1H), 7.34 (dd, *J* = 7.6, 1.6 Hz, 1H), 7.29 – 7.19 (m, 5H), 6.44 (dd, *J* = 7.2, 4.8 Hz, 1H), 6.04 (s, 2H), 4.01 (s, 2H).

***General Procedure for the Synthesis of Intermediate (6-1 ~ 6-4)***

To a solution of **4** (1 eq) in anhydrous 1,4-dioxane, iodobenzene with different substituents (1 eq), Pd_2_(dba)_3_ (0.05 eq), Xantphos (0.11 eq), Cs_2_CO_3_ (1.5 eq) were added at room temperature. The reaction mixture was stirred at 100℃ overnight under inert atmosphere. The resulting mixture was filtered by celite; then the filtrate was diluted with water, extracted with EtOAc, washed with saturated brine, dried over Na_2_SO_4_ and evaporated in vacuo. The residue was purified by silica gel chromatography to afford **6-1 ~ 6-4** as colorless oil.

**3-(benzylthio)-*N*-(4-chlorophenyl)pyridin-2-amine (6-1)**

Colorless oil; yield 91%. MS (ESI) (*m/z*): 326.9 (M+H)^+^. ^1^H NMR (400 MHz, DMSO-*d*_6_) δ 8.19 (s, 1H), 8.06 (dd, *J* = 4.8, 1.6 Hz, 1H), 7.65 (d, *J* = 8.8 Hz, 2H), 7.61 (dd, *J* = 7.6, 1.6 Hz, 1H), 7.29 (d, *J* = 8.8 Hz, 2H), 7.27 – 7.20 (m, 5H), 6.76 (ddd, *J* = 7.6, 4.8, 0.4 Hz, 1H), 4.11 (s, 2H).

**3-(benzylthio)-N-(2,4-difluorophenyl)pyridin-2-amine (6-2)**

Colorless oil; yield 27%. MS (ESI) (*m/z*): 329.0 (M+H)^+^. ^1^H NMR (600 MHz, Chloroform-*d*) δ 8.37 (td, *J* = 9.6, 6.0 Hz, 1H), 8.15 (dd, *J* = 4.8, 1.8 Hz, 1H), 7.84 (s, 1H), 7.49 (dd, *J* = 7.2, 1.8 Hz, 1H), 7.22 – 7.20 (m, 2H), 7.18 – 7.16 (m, 1H), 7.13 – 7.12 (m, 1H), 7.11 (s, 1H), 6.91 – 6.84 (m, 2H), 6.64 (dd, *J* = 7.2, 4.8 Hz, 1H), 3.94 (s, 2H).

**3-(benzylthio)-*N*-(2-chloro-4-fluorophenyl)pyridin-2-amine (6-3)**

Yellow solid; yield 97%. MS (ESI) (*m/z*): 345.09 (M+H)^+^. ^1^H NMR (600 MHz, DMSO-*d*_6_) δ 8.28 (dd, *J* = 9.0, 6.0 Hz, 1H), 8.23 (s, 1H), 8.09 (dd, *J* = 4.8, 1.8 Hz, 1H), 7.67 (dd, *J* = 7.8, 1.8 Hz, 1H), 7.48 (dd, *J* = 8.4, 3.0 Hz, 1H), 7.23 – 7.16 (m, 5H), 6.79 (dd, *J* = 7.8, 4.8 Hz, 1H), 4.13 (s, 2H).

**3-(benzylthio)-*N*-(2,4-dichlorophenyl)pyridin-2-amine (6-4)**

White solid; yield 78%. ^1^H NMR (400 MHz, DMSO-*d*_6_) δ 8.47 (d, *J* = 9.2 Hz, 1H), 8.35 (s, 1H), 8.15 (d, *J* = 5.2 Hz, 1H), 7.74 (d, *J* = 7.6 Hz, 1H), 7.62 (s, 1H), 7.38 (d, *J* = 9.2 Hz, 1H), 7.24 – 7.11 (m, 5H), 6.85 (dd, *J* = 7.2, 4.8 Hz, 1H), 4.13 (s, 2H).

***General Procedure for the Synthesis of*** ***Sulfonyl Chloride Intermediates (7-1 ~ 7-4)***

The corresponding intermediate **6** was suspended in AcOH/H_2_O (V:V=3:1), and N-chlorosuccinimide (3.0 eq) was added slowly under ice bath. Then the mixture was stirred at room temperature for 2 h and monitored by LC-MS. After LC-MS showed the product was formed, the residue was poured into water and extracted with EtOAc. The organic layer was washed with brine, dried with Na_2_SO_4_, filtered, and evaporated in vacuo. The residue was purified by silica gel chromatography to afford sulfonyl chloride. After the product was confirmed by LC-MS, it was directly used in the next step without NMR validation.

***General Procedure for the Synthesis of 1, 1a-1l, 8a-8i, 8k-8m, 8p-8s***

To a solution of amine in anhydrous pyridine was added 1.0 equivalent of intermediate **7** and stirred at room temperature 3-5 h. The reaction mixture was washed with 1 mol/L aqueous HCl, extracted with EtOAc and the combined extracts were washed with water and brine, dried over Na_2_SO_4_, filtered, and evaporated in vacuo. The residue was purified by silica gel chromatography to obtain the final compound.

**2-((4-chlorophenyl)amino)-N-(4-fluorophenyl)pyridine-3-sulfonamide (FBX-1)**

White solid; yield 64%. ^1^H NMR (600 MHz, DMSO-*d*_6_) δ 10.68 (s, 1H), 8.33 – 8.30 (m, 2H), 7.95 (dd, *J* = 7.8, 1.8 Hz, 1H), 7.58 (d, *J* = 9.0 Hz, 2H), 7.38 (d, *J* = 9.0 Hz, 2H), 7.08 (d, *J* = 4.2 Hz, 2H), 7.07 (d, *J* = 1.2 Hz, 2H), 6.90 (dd, *J* = 7.8, 4.8 Hz, 1H). ^13^C NMR (151 MHz, DMSO-*d*_6_) δ 159.51 (d, *J* = 241.8 Hz), 152.51, 150.65, 139.44, 138.12, 133.01, 128.51 (2C), 126.65, 123.57 (d, *J* = 8.6 Hz, 2C), 122.66 (2C), 117.53, 116.26 (d, *J* = 23.0 Hz, 2C), 114.74. MS (ESI) (*m/z*): 376.31 (M+H)^+^. HRMS (ESI) calcd for C_17_H_13_ClFN_3_O_2_S [M-H]^-^: 376.0328; found: 376.0325.

**2-((4-chlorophenyl)amino)-*N*-(4-(trifluoromethyl)phenyl)pyridine-3-sulfonamide (1a)**

White solid; yield 32%. ^1^H NMR (400 MHz, DMSO-*d*_6_) δ 11.35 (s, 1H), 8.33 (d, *J* = 4.8 Hz, 1H), 8.28 (s, 1H), 8.15 (d, *J* = 8.0 Hz, 1H), 7.65 – 7.55 (m, 4H), 7.38 (d, *J* = 8.8 Hz, 2H), 7.26 (d, *J* = 8.0 Hz, 2H), 6.95 (dd, *J* = 7.8, 4.8 Hz, 1H). ^13^C NMR (151 MHz, DMSO-*d*_6_) δ 152.74, 150.52, 139.53, 137.91, 128.42 (2C), 126.75 (q, *J* = 3.7 Hz, 2C), 122.82 (2C), 119.25 (2C), 114.72. MS (ESI) (*m/z*): 428.19 (M+H)^+^. HRMS (ESI) calcd for C_18_H_13_ClF_3_N_3_O_2_S [M+H]^+^: 428.0442; found: 428.0444.

**2-((4-chlorophenyl)amino)-*N*-(3-(trifluoromethyl)phenyl)pyridine-3-sulfonamide (1b)**

White solid; yield 34%. ^1^H NMR (600 MHz, DMSO-*d*_6_) δ 11.16 (s, 1H), 8.37 – 8.25 (m, 2H), 8.06 (d, *J* = 7.8 Hz, 1H), 7.55 (d, *J* = 8.4 Hz, 2H), 7.49 (t, *J* = 7.8 Hz, 1H), 7.41 – 7.33 (m, 4H), 7.32 (s, 1H), 6.94 (dd, *J* = 7.8, 4.8 Hz, 1H). ^13^C NMR (151 MHz, DMSO-*d*_6_) δ 152.74, 150.59, 139.47, 137.94, 130.86, 130.01 (q, *J* = 32.1 Hz), 128.44 (2C), 126.73, 123.86, 122.73 (2C), 116.04 (q, *J* = 4.0 Hz), 114.75. MS (ESI) (*m/z*): 428.19 (M+H)^+^. HRMS (ESI) calcd for C_18_H_13_ClF_3_N_3_O_2_S [M+H]^+^: 428.0442; found: 428.0448.

**2-((4-chlorophenyl)amino)-*N*-(3-iodophenyl)pyridine-3-sulfonamide (1c)**

White solid; yield 30%. ^1^H NMR (600 MHz, DMSO-*d*_6_) δ 10.89 (s, 1H), 8.33 (dd, *J* = 4.8, 1.8 Hz, 1H), 8.26 (s, 1H), 8.04 (dd, *J* = 7.8, 1.8 Hz, 1H), 7.57 (d, *J* = 8.4 Hz, 2H), 7.40 – 7.37 (m, 4H), 7.11 – 7.08 (m, 1H), 7.04 (t, *J* = 7.8 Hz, 1H), 6.95 (dd, *J* = 7.8, 4.8 Hz, 1H). ^13^C NMR (151 MHz, DMSO-*d*_6_) δ 152.71, 150.56, 139.49, 138.12, 137.94, 133.29, 131.39, 128.43(2C), 128.40, 126.73, 122.82(2C), 119.41, 117.22, 114.75, 95.01. MS (ESI) (*m/z*): 486.11 (M+H)^+^. HRMS (ESI) calcd for C_17_H_13_ClIN_3_O_2_S [M+H]^+^: 485.9535; found: 485.9539.

**2-((4-chlorophenyl)amino)-*N*-(4-cyanophenyl)pyridine-3-sulfonamide (1d)**

White solid; yield 44%. ^1^H NMR (600 MHz, DMSO-*d*_6_) δ 11.46 (s, 1H), 8.34 (dd, *J* = 4.8, 1.8 Hz, 1H), 8.25 (s, 1H), 8.17 (dd, *J* = 7.8, 1.8 Hz, 1H), 7.71 (d, *J* = 9.0 Hz, 2H), 7.58 (d, *J* = 9.0 Hz, 2H), 7.38 (d, *J* = 9.0 Hz, 2H), 7.21 (d, *J* = 9.0 Hz, 2H), 6.96 (dd, *J* = 7.8, 4.8 Hz, 1H). ^13^C NMR (151 MHz, DMSO-*d*_6_) δ 152.92, 150.52, 139.66, 137.87, 133.88 (2C), 128.45 (2C), 126.88, 122.98 (2C), 118.84 (2C), 118.54, 117.10, 114.78, 105.96. MS (ESI) (*m/z*): 385.15 (M+H)^+^. HRMS (ESI) calcd for C_18_H_13_ClN_4_O_2_S [M+H]^+^: 385.0521; found: 385.0525.

**2-((4-chlorophenyl)amino)-*N*-(3-cyanophenyl)pyridine-3-sulfonamide (1e)**

White solid; yield 52%. ^1^H NMR (600 MHz, Chloroform-*d*) δ 11.22 (s, 1H), 8.33 (dd, *J* = 4.8, 1.8 Hz, 1H), 8.27 (s, 1H), 8.12 (dd, *J* = 7.8, 1.8 Hz, 1H), 7.57 (d, *J* = 8.4 Hz, 2H), 7.51 (dt, *J* = 7.8, 1.2 Hz, 1H), 7.46 (t, *J* = 7.8 Hz, 1H), 7.42 (t, *J* = 1.8 Hz, 1H), 7.40 – 7.36 (m, 3H), 6.95 (dd, *J* = 7.8, 4.8 Hz, 1H). ^13^C NMR (151 MHz, DMSO-*d*_6_) δ 152.84, 150.53, 139.58, 137.91, 137.78, 131.00, 128.42 (2C), 128.21, 126.77, 124.57, 122.82 (2C), 122.57, 118.05, 117.09, 114.82, 112.27. MS (ESI) (*m/z*): 385.17 (M+H)^+^. HRMS (ESI) calcd for C_18_H_13_ClN_4_O_2_S [M+H]^+^: 385.0521; found: 385.0525.

**2-((4-chlorophenyl)amino)-*N*-(4-methoxyphenyl)pyridine-3-sulfonamide (1f)**

White solid; yield 38%. ^1^H NMR (600 MHz, DMSO-*d*_6_) δ 10.35 (s, 1H), 8.31 (dd, *J* = 4.8, 1.8 Hz, 1H), 8.29 (s, 1H), 7.89 (dd, *J* = 7.8, 1.8 Hz, 1H), 7.56 (d, *J* = 9.0 Hz, 2H), 7.37 (d, *J* = 9.0 Hz, 2H), 6.96 (d, *J* = 9.0 Hz, 2H), 6.89 (dd, *J* = 7.8, 4.8 Hz, 1H), 6.79 (d, *J* = 8.4 Hz, 2H), 3.63 (s, 3H). ^13^C NMR (151 MHz, DMSO-*d*_6_) δ 157.11, 152.24, 150.62, 139.27, 138.10, 128.82, 128.38(2C), 126.43, 124.21(2C), 122.41(2C), 117.73, 114.64, 114.57(2C), 55.14. MS (ESI) (*m/z*): 390.17 (M+H)^+^. HRMS (ESI) calcd for C_18_H_16_ClN_3_O_3_S [M+H]^+^: 390.0674; found: 390.0684.

**2-((4-chlorophenyl)amino)-*N*-(3-methoxyphenyl)pyridine-3-sulfonamide (1g)**

White solid; yield 34%. ^1^H NMR (400 MHz, DMSO-*d*_6_) δ 10.77 (s, 1H), 8.31 (dd, *J* = 4.8, 2.0 Hz, 1H), 8.29 (s, 1H), 8.05 (dd, *J* = 7.6, 2.0 Hz, 1H), 7.59 (d, *J* = 8.8 Hz, 2H), 7.38 (d, *J* = 8.8 Hz, 2H), 7.14 (t, *J* = 8.4 Hz, 1H), 6.93 (dd, *J* = 7.6, 4.8 Hz, 1H), 6.66 – 6.59 (m, 3H), 3.64 (s, 3H). ^13^C NMR (151 MHz, DMSO-*d*_6_) δ 159.81, 152.45, 150.55, 139.52, 138.03, 137.87, 130.30, 128.41 (2C), 126.63, 122.74 (2C), 117.47, 114.63, 112.25, 109.85, 105.94, 55.03. MS (ESI) (*m/z*): 390.18 (M+H)^+^. HRMS (ESI) calcd for C_18_H_16_ClN_3_O_3_S [M+H]^+^: 390.0674; found: 390.0681.

**Ethyl 3-((2-((4-chlorophenyl)amino)pyridine)-3-sulfonamido)benzoate (1h)**

White solid; yield 58%. ^1^H NMR (400 MHz, DMSO-*d*_6_) δ 10.98 (s, 1H), 8.31 (dd, *J* = 4.8, 1.6 Hz, 1H), 8.29 (s, 1H), 8.04 (dd, *J* = 8.0, 1.2 Hz, 1H), 7.65 (s, 1H), 7.61 (d, *J* = 7.2 Hz, 1H), 7.55 (d, *J* = 8.8 Hz, 2H), 7.43 – 7.33 (m, 4H), 6.93 (dd, *J* = 7.6, 4.8 Hz, 1H), 4.25 (q, *J* = 7.2 Hz, 2H), 1.27 (t, *J* = 7.2 Hz, 3H). ^13^C NMR (151 MHz, DMSO-*d*_6_) δ 164.92, 152.66, 150.55, 139.43, 137.94, 137.14, 130.98, 130.00, 128.40 (2C), 126.66, 125.25, 124.82, 122.63 (2C), 120.62, 117.30, 114.73, 60.95, 14.05. MS (ESI) (*m/z*): 432.23 (M+H)^+^. HRMS (ESI) calcd for C_20_H_18_ClN_3_O_4_S [M+H]^+^: 432.0779; found: 432.0783.

**3-((2-((4-chlorophenyl)amino)pyridine)-3-sulfonamido)benzoic acid (1i)**

30 mg **1h** was dissolved in 1 mL of ethanol, and 5 M NaOH (20 eq) was added. After heating under reflux for 7 h, the ethanol was evaporated in vacuo, and the pH of residue was adjusted to acidic with 1 M HCl to give precipitate. The precipitate was collected and dried to obtain **1i**. White solid; yield 96%. ^1^H NMR (600 MHz, DMSO-*d*6) δ 13.05 (s, 1H), 10.95 (s, 1H), 8.34 (s, 1H), 8.30 (dd, *J* = 4.8, 1.8 Hz, 1H), 8.02 (dd, *J* = 7.8, 1.8 Hz, 1H), 7.65 (s, 1H), 7.61 – 7.56 (m, 3H), 7.39 – 7.35 (m, 3H), 7.32 (d, *J* = 7.8 Hz, 1H), 6.91 (dd, *J* = 7.8, 4.8 Hz, 1H). ^13^C NMR (151 MHz, DMSO-*d*_6_) δ 166.64, 152.62, 150.63, 139.43, 138.06, 137.29, 132.04, 129.86, 128.49(2C), 126.73, 125.44, 124.55, 122.79(2C), 120.99, 117.49, 114.73. MS (ESI) (*m/z*): 404.18 (M+H)^+^. HRMS (ESI) calcd for C_18_H_14_ClN_3_O_4_S [M+H]^+^: 404.0466; found: 404.0470.

**2-((4-chlorophenyl)amino)-*N*-(3-ethynylphenyl)pyridine-3-sulfonamide (1j)**

White solid; yield 66%. ^1^H NMR (400 MHz, DMSO-*d*_6_) δ 10.94 (s, 1H), 8.32 (dd, *J* = 4.8, 1.6 Hz, 1H), 8.28 (s, 1H), 8.04 (dd, *J* = 7.6, 1.6 Hz, 1H), 7.58 (d, *J* = 8.8 Hz, 2H), 7.39 (d, *J* = 8.8 Hz, 2H), 7.26 (t, *J* = 8.0 Hz, 1H), 7.17 – 7.09 (m, 3H), 6.93 (dd, *J* = 8.0, 4.8 Hz, 1H), 4.23 (s, 1H). ^13^C NMR (151 MHz, DMSO-*d*_6_) δ 152.64, 150.56, 139.47, 137.95, 137.05, 129.99, 128.43(2C), 127.92, 126.74, 122.90, 122.86(2C), 122.70, 120.80, 117.25, 114.71, 82.59, 81.46. MS (ESI) (*m/z*): 384.20 (M+H)^+^. HRMS (ESI) calcd for C_19_H_14_ClN_3_O_2_S [M+H]^+^: 384.0568; found: 384.0575.

**2-((4-chlorophenyl)amino)-*N*-(6-fluoropyridin-3-yl)pyridine-3-sulfonamide (1k)**

White solid; yield 22%. ^1^H NMR (600 MHz, DMSO-*d*_6_) δ 10.97 (s, 1H), 8.35 (dd, *J* = 4.8, 1.8 Hz, 1H), 8.30 (s, 1H), 7.99 (dd, *J* = 7.8, 1.8 Hz, 1H), 7.89 (d, *J* = 1.8 Hz, 1H), 7.66 (td, *J* = 9.0, 7.2, 3.0 Hz, 1H), 7.57 (d, *J* = 8.4 Hz, 2H), 7.38 (d, *J* = 9.0 Hz, 2H), 7.10 (dd, *J* = 8.4, 3.0 Hz, 1H), 6.93 (dd, *J* = 7.8, 4.8 Hz, 1H). ^13^C NMR (151 MHz, DMSO-*d*_6_) δ 152.79, 150.58, 140.28, 140.18, 139.42, 137.93, 135.53, 135.47, 128.45(2C), 126.69, 122.64(2C), 114.85, 110.46, 110.20. MS (ESI) (*m/z*): 379.17 (M+H)^+^. HRMS (ESI) calcd for C_16_H_12_ClFN_4_O_2_S [M+H]^+^: 379.0426; found: 379.0431.

***N*-benzyl-2-((4-chlorophenyl)amino)pyridine-3-sulfonamide (1l)**

White solid; yield 42%. ^1^H NMR (400 MHz, DMSO-*d6*) δ 8.80 (s, 1H), 8.33 (s, 1H), 8.29 (d, J = 3.6 Hz, 1H), 8.01 (d, J = 8.0 Hz, 1H), 7.60 (d, J = 8.4 Hz, 2H), 7.38 (d, J = 8.0 Hz, 2H), 7.23 – 7.13 (m, 5H), 6.91 (t, J = 6.0 Hz, 1H), 4.08 (s, 2H). ^13^C NMR (151 MHz, DMSO-*d*_6_) δ 151.62, 150.29, 138.66, 138.30, 136.92, 128.38(2C), 128.22(2C), 127.45(2C), 127.27, 126.21, 122.33(2C), 119.35, 114.67, 45.70. MS (ESI) (*m/z*): 374.21 (M+H)^+^. HRMS (ESI) calcd for C_18_H_16_ClN_3_O_2_S [M+H]^+^: 374.0725; found: 374.0733.

***N*-(3-cyanophenyl)-2-((2,4-difluorophenyl)amino)pyridine-3-sulfonamide (8a)**

White solid; yield 63%. ^1^H NMR (600 MHz, DMSO-*d*_6_) δ 11.05 (s, 1H), 8.28 (d, *J* = 3.6 Hz, 1H), 8.21 (s, 1H), 8.08 (d, *J* = 7.2 Hz, 1H), 7.83 – 7.76 (m, 1H), 7.54 – 7.50 (m, 1H), 7.47 (t, *J* = 8.4 Hz, 1H), 7.44 (s, 1H), 7.40 (d, *J* = 7.8 Hz, 1H), 7.37 – 7.31 (m, 1H), 7.08 (t, *J* = 7.8 Hz, 1H), 6.92 (dd, *J* = 7.8, 4.8 Hz, 1H). ^13^C NMR (151 MHz, DMSO-*d*_6_) δ 158.63 (dd, *J* = 243.5, 11.7 Hz), 155.11 (dd, *J* = 247.4, 12.5 Hz), 153.07, 151.12, 139.37, 137.90, 130.98, 128.34, 126.27 (dd, *J* = 9.4, 2.0 Hz), 124.93, 123.51 (dd, *J* = 11.3, 3.7 Hz), 122.98, 118.14, 117.07, 114.83, 112.28, 111.15 (dd, *J* = 21.7, 3.5 Hz), 104.22 (dd, *J* = 27.0, 24.2 Hz). MS (ESI) (*m/z*): 385.23 (M+H)^+^. HRMS (ESI) calcd for C_18_H_12_F_2_N_4_O_2_S [M+H]^+^: 385.0576; found: 385.0574.

**2-((2-chloro-4-fluorophenyl)amino)-*N*-(3-cyanophenyl)pyridine-3-sulfonamide (8b)**

White solid; yield 77%. ^1^H NMR (600 MHz, DMSO-*d*_6_) δ 11.04 (s, 1H), 8.42 (s, 1H), 8.29 (s, 1H), 8.11 (d, *J* = 6.6 Hz, 1H), 7.84 (t, *J* = 6.6 Hz, 1H), 7.56 – 7.48 (m, 2H), 7.45 (s, 2H), 7.42 – 7.37 (m, 1H), 7.22 (t, *J* = 8.4 Hz, 1H), 6.97 (t, *J* = 6.0 Hz, 1H). ^13^C NMR (151 MHz, DMSO-*d*_6_) δ 158.20 (d, *J* = 244.3 Hz), 152.95, 151.01, 139.41, 137.98, 132.60 (d, *J* = 3.3 Hz), 130.98, 128.30, 126.89 (d, *J* = 10.8 Hz), 126.08 (d, *J* = 8.7 Hz), 125.14, 123.23, 118.14, 117.50, 116.56 (d, *J* = 26.1 Hz), 115.23, 114.54 (d, *J* = 21.8 Hz), 112.27. MS (ESI) (*m/z*): 401.31 (M-H)^-^. HRMS (ESI) calcd for C_18_H_12_ClFN_4_O_2_S [M+H]^+^: 401.0281; found: 401.0277.

*****N*-(3-cyanophenyl)-2-((2,4-dichlorophenyl)amino)pyridine-3-sulfonamide (8c)**

White solid; yield 90%. ^1^H NMR (400 MHz, DMSO-*d*_6_) δ 11.07 (s, 1H), 8.61 (s, 1H), 8.35 (dd, *J* = 4.8, 1.6 Hz, 1H), 8.13 (dd, *J* = 8.0, 2.0 Hz, 1H), 7.98 (d, *J* = 9.2 Hz, 1H), 7.68 (d, *J* = 2.4 Hz, 1H), 7.52 – 7.35 (m, 5H), 7.03 (dd, *J* = 8.0, 4.8 Hz, 1H). ^13^C NMR (151 MHz, DMSO-*d*_6_) δ 152.79, 150.38, 139.32, 137.77, 134.96, 130.91, 128.69, 128.42, 127.45, 127.42, 125.53, 125.45, 124.34, 123.57, 118.09, 118.02, 115.78, 112.21. MS (ESI) (*m/z*): 419.18 (M+H)^+^. HRMS (ESI) calcd for C_18_H_12_Cl_2_N_4_O_2_S [M+H]^+^: 419.0131; found: 419.0135.

***N*-(3-cyano-4-methoxyphenyl)-2-((2,4-dichlorophenyl)amino)pyridine-3-sulfonamide (8d)**

**

White solid; yield 76%. ^1^H NMR (600 MHz, DMSO-*d*_6_) δ 10.50 (s, 1H), 8.57 (s, 1H), 8.40 (dd, *J* = 4.8, 1.8 Hz, 1H), 8.05 (dd, *J* = 7.8, 1.8 Hz, 1H), 8.03 (d, *J* = 9.0 Hz, 1H), 7.63 (d, *J* = 2.4 Hz, 1H), 7.36 (dd, *J* = 9.0, 2.4 Hz, 1H), 7.33 (d, *J* = 2.4 Hz, 1H), 7.27 (dd, *J* = 9.0, 2.4 Hz, 1H), 7.07 (s, 1H), 7.06 (dd, *J* = 4.8, 3.0 Hz, 1H), 3.74 (s, 3H). ^13^C NMR (151 MHz, DMSO-*d*_6_) δ 158.81, 152.60, 150.29, 139.17, 134.91, 130.22, 129.16, 128.55, 128.03, 127.31, 126.71, 124.34, 122.68, 118.61, 116.08, 115.44, 113.25, 100.72, 56.33. MS (ESI) (*m/z*): 449.20 (M+H)^+^. HRMS (ESI) calcd for C_19_H_14_Cl_2_N_4_O_3_S [M+H]^+^: 449.0236; found: 449.0240.

***N*-(5-cyano-2-fluorophenyl)-2-((2,4-dichlorophenyl)amino)pyridine-3-sulfonamide (8e)**

White solid; yield 57%. ^1^H NMR (600 MHz, DMSO-*d*_6_) δ 10.95 (s, 1H), 8.61 (s, 1H), 8.40 (dd, *J* = 4.8, 1.8 Hz, 1H), 8.09 – 8.04 (m, 2H), 7.78 (dd, *J* = 7.2, 2.4 Hz, 1H), 7.70 – 7.66 (m, 1H), 7.63 (d, *J* = 2.4 Hz, 1H), 7.41 – 7.34 (m, 2H), 7.03 (dd, *J* = 7.8, 4.8 Hz, 1H). ^13^C NMR (151 MHz, DMSO-*d*_6_) δ 159.58, 157.88, 152.79, 150.27, 139.08, 134.96, 131.48, 128.63, 127.48, 127.04, 124.81, 123.41, 119.14, 118.04, 117.90, 117.37, 115.89, 108.30 (d, *J* = 3.7 Hz). MS (ESI) (*m/z*): 437.17 (M+H)^+^. HRMS (ESI) calcd for C_18_H_11_Cl_2_FN_4_O_2_S [M+H]^+^: 437.0037; found: 437.0040.

***N*-(3-cyano-4-fluorophenyl)-2-((2,4-dichlorophenyl)amino)pyridine-3-sulfonamide (8f)**

White solid; yield 76%. ^1^H NMR (600 MHz, DMSO-*d*_6_) δ 10.91 (s, 1H), 8.57 (s, 1H), 8.39 (dd, *J* = 4.8, 1.8 Hz, 1H), 8.10 (dd, *J* = 7.8, 1.8 Hz, 1H), 8.01 (d, *J* = 9.0 Hz, 1H), 7.65 (d, *J* = 2.4 Hz, 1H), 7.54 (dd, *J* = 5.4, 2.4 Hz, 1H), 7.41 – 7.37 (m, 3H), 7.05 (dd, *J* = 7.8, 4.8 Hz, 1H). ^13^C NMR (151 MHz, DMSO-*d*_6_) δ 159.78 (d, *J* = 254.3 Hz), 152.83, 150.28, 139.30, 134.89, 133.72 (d, *J* = 2.9 Hz), 129.46 (d, *J* = 8.8 Hz), 128.64, 127.43, 127.14, 126.37, 124.92, 123.55, 118.24, 117.85 (d, *J* = 21.3 Hz), 116.01, 113.19, 100.87 (d, *J* = 16.6 Hz). MS (ESI) (*m/z*): 437.17 (M+H)^+^. HRMS (ESI) calcd for C_18_H_11_Cl_2_FN_4_O_2_S [M+H]^+^: 437.0037; found: 437.0040.

***N*-(5-cyanopyridin-3-yl)-2-((2,4-dichlorophenyl)amino)pyridine-3-sulfonamide (8g)**

**

White solid; yield 56%. ^1^H NMR (400 MHz, DMSO-*d*_6_) δ 8.70 – 8.66 (m, 1H), 8.60 (s, 1H), 8.51 (d, *J* = 2.4 Hz, 1H), 8.37 (dd, *J* = 4.8, 2.0 Hz, 1H), 8.20 (dd, *J* = 8.0, 2.0 Hz, 1H), 8.01 (d, *J* = 8.8 Hz, 1H), 7.96 (t, *J* = 2.4 Hz, 1H), 7.67 (d, *J* = 2.8 Hz, 1H), 7.40 (dd, *J* = 9.2, 1.6 Hz, 1H), 7.04 (dd, *J* = 7.6, 4.8 Hz, 1H). ^13^C NMR (151 MHz, DMSO-*d*_6_) δ 152.98, 150.36, 148.10, 145.70, 139.39, 134.93, 133.95, 130.56, 128.69, 127.51, 125.54, 124.47, 118.10, 116.16, 115.91, 109.34. MS (ESI) (*m/z*): 420.17 (M+H)^+^. HRMS (ESI) calcd for C_17_H_11_Cl_2_N_5_O_2_S [M+H]^+^: 420.0083; found: 420.0085.

***N*-(2-cyanopyridin-4-yl)-2-((2,4-dichlorophenyl)amino)pyridine-3-sulfonamide (8h)**

White solid; yield 31%. ^1^H NMR (400 MHz, DMSO-*d*_6_) δ 8.57 (s, 1H), 8.49 (d, *J* = 6.0 Hz, 1H), 8.39 – 8.32 (m, 2H), 7.95 (d, *J* = 8.4 Hz, 1H), 7.70 (d, *J* = 2.4 Hz, 1H), 7.53 (d, *J* = 2.0 Hz, 1H), 7.41 (dd, *J* = 8.8, 2.4 Hz, 1H), 7.33 (dd, *J* = 5.6, 2.0 Hz, 1H), 7.06 (dd, *J* = 7.6, 4.8 Hz, 1H). ^13^C NMR (151 MHz, MeOD) δ 154.00, 152.84, 152.50, 149.23, 140.71, 136.36, 135.30, 130.06, 129.85, 128.45, 127.30, 125.69, 120.28, 118.97, 117.82, 116.91, 116.67. MS (ESI) (*m/z*): 420.17 (M+H)^+^. HRMS (ESI) calcd for C_17_H_11_Cl_2_N_5_O_2_S [M+H]^+^: 420.0083; found: 420.0082.

***N*-(5-cyanothiophen-2-yl)-2-((2,4-dichlorophenyl)amino)pyridine-3-sulfonamide (8i)**

White solid; yield 33%. ^1^H NMR (600 MHz, DMSO-*d*_6_) δ 8.78 (s, 1H), 8.39 (d, *J* = 3.0 Hz, 1H), 8.17 (d, *J* = 9.0 Hz, 1H), 8.10 (d, *J* = 7.8 Hz, 1H), 7.68 (d, *J* = 2.4 Hz, 1H), 7.60 (d, *J* = 4.2 Hz, 1H), 7.41 (dd, *J* = 9.0, 2.4 Hz, 1H), 7.05 (dd, *J* = 7.8, 4.8 Hz, 1H), 6.60 (d, *J* = 3.6 Hz, 1H). ^13^C NMR (151 MHz, DMSO-*d*_6_) δ 152.58, 150.36, 139.16, 138.58, 135.20, 128.74, 127.56, 127.01, 124.85, 123.71, 115.87, 114.65. MS (ESI) (*m/z*): 425.12 (M+H)^+^. HRMS (ESI) calcd for C_16_H_10_Cl_2_N_4_O_2_S_2_ [M+H]^+^: 424.9695; found: 424.9699.

**2-((2,4-dichlorophenyl)amino)-*N*-(3-(5-oxo-4,5-dihydro-1,2,4-oxadiazol-3-yl)phenyl)pyridine-3-sulfonamide (8k)**

White solid; yield 75%. ^1^H NMR (600 MHz, DMSO-*d*_6_) δ 8.64 (s, 1H), 8.33 (dd, *J* = 4.8, 1.8 Hz, 1H), 8.10 (dd, *J* = 7.8, 1.2 Hz, 1H), 7.97 (d, *J* = 8.4 Hz, 1H), 7.63 (d, *J* = 2.4 Hz, 1H), 7.60 (s, 1H), 7.43 (d, *J* = 4.8 Hz, 2H), 7.35 (dd, *J* = 9.0, 2.4 Hz, 1H), 7.32 – 7.27 (m, 1H), 7.01 (dd, *J* = 7.8, 4.8 Hz, 1H). ^13^C NMR (151 MHz, DMSO-*d*_6_) δ 160.18, 157.03, 152.72, 150.47, 139.30, 137.78, 135.07, 130.56, 128.69, 127.45, 127.36, 125.47, 124.66, 124.62, 124.20, 122.70, 118.46, 118.14, 115.82. MS (ESI) (*m/z*): 478.16 (M+H)^+^. HRMS (ESI) calcd for C_19_H_13_Cl_2_N_5_O_4_S [M+H]^+^: 478.0138; found: 478.0141.

***N*-(3-(1*H*-tetrazol-5-yl)phenyl)-2-((2,4-dichlorophenyl)amino)pyridine-3-sulfonamide (8l)**

White solid; yield 29%.^1^H NMR (600 MHz, DMSO-*d*_6_) δ 8.64 (s, 1H), 8.32 (dd, *J* = 4.8, 1.8 Hz, 1H), 8.11 (dd, *J* = 7.8, 1.8 Hz, 1H), 7.95 (d, *J* = 9.0 Hz, 1H), 7.86 – 7.84 (m, 1H), 7.68 (d, *J* = 7.8 Hz, 1H), 7.59 (d, *J* = 2.4 Hz, 1H), 7.45 (t, *J* = 7.8 Hz, 1H), 7.32 (dd, *J* = 9.0, 2.4 Hz, 1H), 7.26 (dd, *J* = 8.4, 1.2 Hz, 1H), 7.01 (dd, *J* = 7.8, 4.8 Hz, 1H). ^13^C NMR (151 MHz, DMSO) δ 152.61, 150.39, 139.24, 137.64, 134.97, 130.54, 129.65, 128.56, 127.31, 127.22, 125.36, 124.10, 123.55, 123.28, 119.41, 118.32, 115.70. MS (ESI) (*m/z*): 462.12 (M+H)^+^. HRMS (ESI) calcd for C_18_H_13_Cl_2_N_7_O_2_S [M+H]^+^: 462.0301; found: 462.0303.

**2-((2,4-dichlorophenyl)amino)-*N*-(3-oxo-3,4-dihydro-2*H*-benzo[*b*][1,4]oxazin-6-yl)pyridine-3-sulfonamide (8m)**

White solid; yield 36%. ^1^H NMR (400 MHz, DMSO-*d*_6_) δ 10.67 (s, 1H), 10.33 (s, 1H), 8.63 (s, 1H), 8.37 (dd, *J* = 4.8, 1.6 Hz, 1H), 8.06 (d, *J* = 8.8 Hz, 1H), 8.02 (dd, *J* = 7.6, 1.6 Hz, 1H), 7.62 (d, *J* = 2.4 Hz, 1H), 7.36 (dd, *J* = 9.2, 2.8 Hz, 1H), 7.04 (dd, *J* = 8.0, 4.8 Hz, 1H), 6.74 (d, *J* = 8.4 Hz, 1H), 6.71 (d, *J* = 2.4 Hz, 1H), 6.55 (dd, *J* = 8.4, 2.4 Hz, 1H), 4.37 (s, 2H). ^13^C NMR (151 MHz, DMSO-*d*_6_) δ 164.51, 152.30, 150.34, 140.88, 139.07, 135.08, 128.50, 127.64, 127.26, 126.65, 124.59, 123.14, 117.48, 116.53, 115.82, 110.71, 66.47. MS (ESI) (*m/z*): 465.18 (M+H)^+^. HRMS (ESI) calcd for C_19_H_14_Cl_2_N_4_O_4_S [M+H]^+^: 465.0186; found: 465.0189.

**Methyl 3-((2-((2,4-dichlorophenyl)amino)pyridine)-3-sulfonamido)benzoate (8p, FBX-1991)**

White Solid; yield 63%. ^1^H NMR (400 MHz, DMSO-*d*_6_) δ 10.83 (s, 1H), 8.63 (s, 1H), 8.33 (d, *J* = 4.4 Hz, 1H), 8.09 – 8.04 (m, 1H), 7.96 (d, *J* = 8.8 Hz, 1H), 7.68 (s, 1H), 7.65 (d, *J* = 2.4 Hz, 1H), 7.59 (d, *J* = 7.2 Hz, 1H), 7.42 – 7.33 (m, 3H), 7.01 (dd, *J* = 8.0, 4.8 Hz, 1H), 3.79 (s, 3H). ^13^C NMR (151 MHz, DMSO-*d*_6_) δ 165.41, 152.65, 150.36, 139.23, 137.07, 134.97, 130.65, 129.96, 128.61, 127.36, 127.19, 125.80, 125.59, 125.27, 123.91, 121.65, 118.29, 115.77, 52.29. MS (ESI) (*m/z*): 452.18 (M+H)^+^. HRMS (ESI) calcd for C_19_H_15_Cl_2_N_3_O_4_S [M+H]^+^: 452.0233; found: 452.0230.

**Ethyl 3-((2-((2,4-dichlorophenyl)amino)pyridine)-3-sulfonamido)benzoate (8q)**

White solid; yield 92%. ^1^H NMR (400 MHz, DMSO-*d*_6_) δ 10.81 (s, 1H), 8.62 (s, 1H), 8.34 (dd, *J* = 4.8, 1.2 Hz, 1H), 8.07 (dd, *J* = 8.0, 0.8 Hz, 1H), 7.96 (d, *J* = 8.8 Hz, 1H), 7.67 (s, 1H), 7.64 (d, *J* = 2.4 Hz, 1H), 7.61 – 7.56 (m, 1H), 7.41 – 7.33 (m, 3H), 7.02 (dd, *J* = 8.0, 4.8 Hz, 1H), 4.25 (q, *J* = 7.2 Hz, 2H), 1.27 (t, *J* = 7.2 Hz, 3H). ^13^C NMR (151 MHz, DMSO-*d*_6_) δ 164.87, 152.62, 150.35, 139.22, 137.03, 134.96, 130.91, 129.89, 128.59, 127.33, 127.13, 125.78, 125.54, 125.20, 123.82, 121.64, 118.32, 115.76, 60.92, 14.05. MS (ESI) (*m/z*): 466.17 (M+H)^+^. HRMS (ESI) calcd for C_20_H_17_Cl_2_N_3_O_4_S [M+H]^+^: 466.0390; found: 466.0394.

**Isopropyl 3-((2-((2,4-dichlorophenyl)amino)pyridine)-3-sulfonamido)benzoate(8r)**

White solid; yield 64%. ^1^H NMR (600 MHz, DMSO-*d*_6_) δ 10.79 (s, 1H), 8.63 (s, 1H), 8.34 (dd, *J* = 4.8, 1.2 Hz, 1H), 8.08 (dd, *J* = 7.8, 1.8 Hz, 1H), 7.97 (d, *J* = 9.0 Hz, 1H), 7.64 (s, 1H), 7.63 (d, *J* = 2.4 Hz, 1H), 7.57 (d, *J* = 7.2 Hz, 1H), 7.39 – 7.33 (m, 3H), 7.02 (dd, *J* = 7.8, 4.8 Hz, 1H), 5.06 (hept, *J* = 6.0 Hz, 1H), 1.26 (d, *J* = 6.0 Hz, 6H). ^13^C NMR (151 MHz, DMSO-*d*_6_) δ 164.35, 152.58, 150.37, 139.22, 137.06, 134.96, 131.25, 129.82, 128.59, 127.31, 127.10, 125.74, 125.48, 125.18, 123.81, 121.66, 118.37, 115.73, 68.38, 21.55 (2C). MS (ESI) (*m/z*): 480.19 (M+H)^+^. HRMS (ESI) calcd for C_21_H_19_Cl_2_N_3_O_4_S [M+H]^+^: 480.0546; found: 480.0551.

***Tert*-butyl 3-((2-((2,4-dichlorophenyl)amino)pyridine)-3-sulfonamido)benzoate (8s)**

White solid; yield 63%. ^1^H NMR (600 MHz, DMSO-*d*_6_) δ 10.77 (s, 1H), 8.61 (s, 1H), 8.35 (dd, *J* = 4.8, 1.8 Hz, 1H), 8.09 (dd, *J* = 7.8, 1.2 Hz, 1H), 7.96 (d, *J* = 9.0 Hz, 1H), 7.62 (d, *J* = 2.4 Hz, 1H), 7.60 (s, 1H), 7.52 (d, *J* = 7.8 Hz, 1H), 7.38 – 7.29 (m, 3H), 7.03 (dd, *J* = 7.8, 4.8 Hz, 1H), 1.49 (s, 9H). ^13^C NMR (151 MHz, DMSO-*d*_6_) δ 164.04, 152.59, 150.38, 139.21, 136.89, 134.94, 132.36, 129.69, 128.59, 127.30, 127.09, 125.53, 125.48, 125.15, 123.80, 121.67, 118.39, 115.75, 80.98, 27.66 (3C). MS (ESI) (*m/z*): 492.53 (M-H)^-^. HRMS (ESI) calcd for C_22_H_21_Cl_2_N_3_O_4_S [M+H]^+^: 494.0703; found: 494.0709.

**Scheme S2. Synthetic Route of Compounds 8n, 8o*^a^***

*^a^* Reagents and conditions: (a) Py, r.t., 3-5 h; (b) 4 M HCl in 1,4-Dioxane, r.t.; (c) Acetic anhydride, K_2_CO_3_, DMF, 0℃ to r.t.

***N*-(3-((*tert*-butyldimethylsilyl)oxy)phenyl)-2-((2,4 dichlorophenyl)amino)pyridine-3-sulfonamide (10)**

Yellow solid; yield 74%. MS (ESI) (*m/z*): 524.10 (M+H)^+^. ^1^H NMR (600 MHz, DMSO-*d*_6_) δ 10.59 (s, 1H), 8.75 (s, 1H), 8.33 (dd, *J* = 4.8, 1.8 Hz, 1H), 8.05 (d, *J* = 8.4 Hz, 1H), 8.01 (dd, *J* = 7.8, 1.8 Hz, 1H), 7.67 (d, *J* = 2.4 Hz, 1H), 7.39 (dd, *J* = 9.0, 2.4 Hz, 1H), 7.10 (t, *J* = 8.4 Hz, 1H), 6.99 (dd, *J* = 7.8, 4.8 Hz, 1H), 6.71 (dd, *J* = 7.8, 1.2 Hz, 1H), 6.56 (t, *J* = 2.4 Hz, 1H), 6.52 (dd, *J* = 8.4, 1.8 Hz, 1H), 0.86 (s, 9H), 0.06 (s, 6H).

**2-((2,4-dichlorophenyl)amino)-*N*-(3-hydroxyphenyl)pyridine-3-sulfonamide (8n)**

Compound **10** (320 mg, 0.6 mmol) was added to a 4 M HCl in 1,4-dioxane (5 mL) and reacted at room temperature. After the reaction was completed by TLC monitoring, the reaction solvent was evaporated in vacuo under reduced pressure, and the residue was added with water and adjusted the pH to neutral with saturated NaHCO_3_, extracted with EtOAc. The organic phase was washed with saturated brine, dried over Na_2_SO_4_, and purified by column chromatography to obtain white solid (yield 69%). ^1^H NMR (600 MHz, DMSO-*d*_6_) δ 10.49 (s, 1H), 9.49 (s, 1H), 8.70 (s, 1H), 8.32 (dd, *J* = 4.8, 1.8 Hz, 1H), 8.05 – 8.01 (m, 2H), 7.67 (d, *J* = 2.4 Hz, 1H), 7.39 (dd, *J* = 9.0, 2.4 Hz, 1H), 7.01 – 6.97 (m, 2H), 6.54 (t, *J* = 1.8 Hz, 1H), 6.51 (d, *J* = 7.8 Hz, 1H), 6.42 (dd, *J* = 7.8, 1.8 Hz, 1H). ^13^C NMR (151 MHz, DMSO-*d*_6_) δ 157.98, 152.29, 150.40, 139.13, 137.65, 135.14, 129.97, 128.62, 127.37, 127.18, 125.50, 124.36, 118.49, 115.50, 111.95, 111.39, 107.91. MS (ESI) (*m/z*): 410.18 (M+H)^+^. HRMS (ESI) calcd for C_17_H_13_Cl_2_N_3_O_3_S [M+H]^+^: 410.0127; found: 410.0131.

**3-((2-((2,4-dichlorophenyl)amino)pyridine)-3-sulfonamido)phenyl acetate (8o)**

Compound **8n** (30 mg, 1.2 eq) and K_2_CO_3_ (12 mg, 0.9 eq) were dissolved in anhydrous DMF, and 8 *μ*L acetic anhydride (1.2 eq) was added under ice bath and reacted at room temperature. After the reaction was completed by TLC monitoring, the reaction mixture was washed with water, extracted with EtOAc, and the organic phase was washed with saturated brine, dried over Na_2_SO_4_, and purified by column chromatography. White solid; yield 64%. ^1^H NMR (600 MHz, DMSO-*d*_6_) δ 10.82 (s, 1H), 8.69 (s, 1H), 8.33 (dd, *J* = 4.8, 1.8 Hz, 1H), 8.06 (dd, *J* = 7.8, 1.8 Hz, 1H), 8.02 (d, *J* = 9.0 Hz, 1H), 7.68 (d, *J* = 2.4 Hz, 1H), 7.40 (dd, *J* = 9.0, 2.4 Hz, 1H), 7.26 (t, *J* = 8.4 Hz, 1H), 6.99 (dd, *J* = 7.8, 4.8 Hz, 1H), 6.96 (d, *J* = 8.4 Hz, 1H), 6.89 (s, 1H), 6.81 (d, *J* = 7.8 Hz, 1H), 2.22 (s, 3H). ^13^C NMR (151 MHz, DMSO-*d*_6_) δ 168.91, 152.62, 150.95, 150.45, 139.32, 137.79, 135.15, 130.26, 128.72, 127.49, 127.40, 125.68, 124.59, 118.24, 118.14, 117.84, 115.66, 114.11, 20.86. MS (ESI) (*m/z*): 450.34 (M-H)^-^. HRMS (ESI) calcd for C_19_H_15_C_l2_N_3_O_4_S [M-H]^-^: 450.0088; found: 450.0092.

**Scheme S3. Synthetic Route of Compounds 8j, 8t-8v*^a^***

*^a^* Reagents and Conditions: (a) 5 M NaOH, EtOH, r.t.; (b) ROH, DMAP, DCC, Et_3_N, DCM, 0°C to r.t.

**3-((2-((2,4-dichlorophenyl)amino)pyridine)-3-sulfonamido)benzoic acid (8j, FBX-1872)**

Compound **8j** was prepared from **8q** by the similar procedure as that described for **1i**. White solid; 98%. ^1^H NMR (600 MHz, DMSO-*d*_6_) δ 13.02 (s, 1H), 10.81 (s, 1H), 8.66 (s, 1H), 8.32 (dd, *J* = 4.8, 1.8 Hz, 1H), 8.05 (dd, *J* = 7.8, 1.8 Hz, 1H), 7.99 (d, *J* = 8.4 Hz, 1H), 7.66 (s, 1H), 7.65 (d, *J* = 1.8 Hz, 1H), 7.58 (d, *J* = 7.2 Hz, 1H), 7.39 – 7.32 (m, 3H), 7.00 (dd, *J* = 7.8, 4.8 Hz, 1H). ^13^C NMR (151 MHz, DMSO) δ 166.61, 152.57, 150.46, 139.23, 137.24, 135.11, 131.97, 129.74, 128.70, 127.43, 127.27, 125.67, 125.47, 125.38, 124.22, 121.94, 118.48, 115.72. MS (ESI) (*m/z*): 438.11 (M+H)^+^. HRMS (ESI) calcd for C_18_H_13_Cl_2_N_3_O_4_S [M+H]^+^: 438.0077; found: 438.0081.

**Hexyl 3-((2-((2,4-dichlorophenyl)amino)pyridine)-3-sulfonamido)benzoate (8t)**

Compound **8j** (60 mg, 1 eq), n-hexanol (5 eq), DMAP (1.2 eq), and Et_3_N (1.5 eq) were dissolved in anhydrous DCM, and DCC (1.1 eq) was added by syringe under an inert atmosphere at 0°C, and the mixture was stirred at room temperature. After the reaction was completed by TLC monitoring, DCC was filtered out, and the filtrate was washed with 1 M HCl, and the organic phase was washed with saturated brine, dried over Na_2_SO_4_, and purified by column chromatography to obtain **8t**. White solid; yield 60%. ^1^H NMR (600 MHz, DMSO-*d*_6_) δ 10.82 (s, 1H), 8.62 (s, 1H), 8.34 (dd, *J* = 4.8, 1.2 Hz, 1H), 8.07 (dd, *J* = 7.8, 1.8 Hz, 1H), 7.96 (d, *J* = 9.0 Hz, 1H), 7.67 (s, 1H), 7.63 (d, *J* = 2.4 Hz, 1H), 7.58 (d, *J* = 7.2 Hz, 1H), 7.39 (t, *J* = 7.8 Hz, 1H), 7.36 (d, *J* = 2.4 Hz, 1H), 7.35 (d, *J* = 1.8 Hz, 1H), 7.01 (dd, *J* = 8.4, 4.8 Hz, 1H), 4.19 (t, *J* = 6.6 Hz, 2H), 1.64 (p, *J* = 6.6 Hz, 2H), 1.34 (p, *J* = 7.2 Hz, 2H), 1.31 – 1.25 (m, 4H), 0.86 (t, *J* = 6.6 Hz, 3H). ^13^C NMR (151 MHz, DMSO-*d*_6_) δ 164.90, 152.61, 150.37, 139.19, 137.05, 134.95, 130.91, 129.92, 128.58, 127.31, 127.14, 125.78, 125.52, 125.22, 123.85, 121.57, 118.29, 115.73, 64.85, 30.85, 28.03, 25.06, 21.98, 13.87. MS (ESI) (*m/z*): 522.29 (M+H)^+^. HRMS (ESI) calcd for C_24_H_25_Cl_2_N_3_O_4_S [M+H]^+^: 522.1016; found: 522.1023.

**Cycloheptyl 3-((2-((2,4-dichlorophenyl)amino)pyridine)-3-sulfonamido)benzoate (8u)**

Compound **8u** was prepared from **8j** and cycloheptanol by the similar procedure as that described for **8t**. ^1^H NMR (600 MHz, DMSO-*d*_6_) δ 10.81 (s, 1H), 8.63 (s, 1H), 8.34 (dd, *J* = 4.8, 1.8 Hz, 1H), 8.07 (dd, *J* = 7.8, 1.8 Hz, 1H), 7.97 (d, *J* = 9.0 Hz, 1H), 7.64 (s, 1H), 7.63 (d, *J* = 2.4 Hz, 1H), 7.57 (d, *J* = 7.2 Hz, 1H), 7.38 (t, *J* = 7.8 Hz, 2H), 7.37 – 7.32 (m, 2H), 7.02 (dd, *J* = 8.4, 4.8 Hz, 1H), 5.03 (tt, *J* = 7.8, 4.2 Hz, 1H), 1.91 – 1.83 (m, 2H), 1.72 – 1.66 (m, 2H), 1.64 – 1.57 (m, 2H), 1.57 – 1.52 (m, 4H), 1.50 – 1.41 (m, 2H). ^13^C NMR (151 MHz, DMSO) δ 164.11, 152.61, 150.38, 139.19, 137.02, 134.95, 131.35, 129.86, 128.59, 127.31, 127.09, 125.75, 125.51, 125.13, 123.75, 121.55, 118.33, 115.74, 75.16, 33.09, 27.72, 22.27. MS (ESI) (*m/z*): 534.0 (M+H)^+^. HRMS (ESI) calcd for C_25_H_25_Cl_2_N_3_O_4_S [M+H]^+^: 534.1016; found: 534.1023.

**(6-((3-((2-((2,4-dichlorophenyl)amino)pyridine)-3 sulfonamido)benzoyl)oxy)hexyl)triphenylphosphonium bromide (8v)**

Compound **8v** was prepared from **8j** and (6-hydroxyhexyl)triphenylphosphine bromide by the similar procedure as that described for **8t**. White solid; yield 16%. ^1^H NMR (600 MHz, DMSO-*d*_6_) δ 9.82 (s, 1H), 8.37 (d, *J* = 9.0 Hz, 1H), 8.17 (d, *J* = 3.0 Hz, 1H), 8.04 (dd, *J* = 7.8, 1.8 Hz, 1H), 7.87 (t, *J* = 6.6 Hz, 3H), 7.82 – 7.78 (m, 4H), 7.77 (s, 2H), 7.77 – 7.73 (m, 6H), 7.54 (d, *J* = 2.4 Hz, 1H), 7.52 (s, 1H), 7.32 (dd, *J* = 9.0, 2.4 Hz, 1H), 7.19 (s, 1H), 7.14 – 7.10 (m, 2H), 6.89 (dd, *J* = 7.6, 4.8 Hz, 1H), 4.13 (t, *J* = 6.0 Hz, 2H), 3.60 – 3.53 (m, 2H), 1.62 – 1.48 (m, 6H), 1.42 – 1.36 (m, 2H). ^13^C NMR (151 MHz, DMSO-*d*_6_) δ 166.16, 150.52, 134.93 (d, *J* = 3.0 Hz), 133.63 (d, *J* = 10.3 Hz), 130.30 (d, *J* = 12.5 Hz), 130.07, 128.68, 128.51, 127.26, 125.54, 121.55, 118.61 (d, *J* = 85.8 Hz), 115.34, 64.10, 29.55 (d, *J* = 16.7 Hz), 27.91, 24.80, 21.77 (d, *J* = 4.2 Hz), 20.23 (d, *J* = 50.3 Hz). MS (ESI) (*m/z*): 782.45 (M+H)^+^. HRMS (ESI) calcd for C_42_H_39_Cl_2_N_3_O_4_PS [M+H]^+^: 782.1770; found: 782.1786.

**Synthesis of compounds 11a-11d**

Compounds **11a-11d** were prepared by the similar procedures described in Scheme S1, except that **2** in the first step was replaced by the corresponding amine substrate.

***N*-(3-cyanophenyl)-3-((2,4-dichlorophenyl)amino)pyridine-4-sulfonamide (11a)**

Yellow solid; yield 43%. ^1^H NMR (600 MHz, DMSO-*d*_6_) δ 11.15 (s, 1H), 8.41 (s, 1H), 8.26 (d, *J* = 5.4 Hz, 1H), 7.69 (d, *J* = 4.8 Hz, 1H), 7.67 (d, *J* = 2.4 Hz, 1H), 7.51 (s, 1H), 7.44 (dt, *J* = 7.2, 1.8 Hz, 1H), 7.43 – 7.40 (m, 2H), 7.38 – 7.36 (m, 1H), 7.34 (dd, *J* = 9.0, 2.4 Hz, 1H), 7.29 (d, *J* = 8.4 Hz, 1H). ^13^C NMR (151 MHz, DMSO-*d*_6_) δ 141.56, 140.80, 137.53, 135.96, 135.28, 130.93, 130.92, 129.54, 128.38, 128.29, 127.73, 126.04, 125.42, 123.52, 122.44, 122.08, 118.02, 112.25. MS (ESI) (*m/z*): 419.18 (M+H)^+^. HRMS (ESI) calcd for C_18_H_12_Cl_2_N_4_O_2_S [M+H]^+^: 419.0131; found: 419.0140.

***N*-(3-cyanophenyl)-4-((2,4-dichlorophenyl)amino)pyridine-3-sulfonamide (11b)**

White solid; yield 8%. ^1^H NMR (600 MHz, DMSO-*d*_6_) δ 8.68 (s, 1H), 8.25 (d, *J* = 6.0 Hz, 1H), 7.81 (d, *J* = 1.8 Hz, 1H), 7.52 – 7.41 (m, 6H), 6.67 (d, *J* = 6.0 Hz, 1H). ^13^C NMR (151 MHz, DMSO-*d*_6_) δ 154.10, 150.65, 148.01, 138.60, 134.69, 131.41, 131.25, 130.33, 130.08, 129.02, 128.46, 127.83, 125.06, 123.15, 118.77, 118.59, 112.65, 109.46. MS (ESI) (*m/z*): 419.18 (M+H)^+^. HRMS (ESI) calcd for C_18_H_12_Cl_2_N_4_O_2_S [M+H]^+^: 419.0131; found: 419.0141.

***N*-(3-cyanophenyl)-3-((2,4-dichlorophenyl)amino)pyridine-2-sulfonamide (11c)**

White solid; yield 35%. ^1^H NMR (600 MHz, DMSO-*d*_6_) δ 11.20 (s, 1H), 8.15 (dd, *J* = 4.2, 1.2 Hz, 1H), 8.08 (s, 1H), 7.73 (d, *J* = 2.4 Hz, 1H), 7.54 – 7.49 (m, 2H), 7.47 (d, *J* = 4.2 Hz, 1H), 7.47 – 7.43 (m, 3H), 7.41 – 7.36 (m, 2H). ^13^C NMR (151 MHz, DMSO-*d*_6_) δ 140.44, 140.18, 138.66, 138.62, 135.63, 130.64, 129.62, 128.65, 128.29, 128.20, 127.55, 126.99, 125.41, 124.63, 123.45, 122.41, 118.22, 111.91. MS (ESI) (*m/z*): 419.14 (M+H)^+^. HRMS (ESI) calcd for C_18_H_12_Cl_2_N_4_O_2_S [M+H]^+^: 419.0131; found: 419.0133.

***N*-(3-cyanophenyl)-2-((2,4-dichlorophenyl)amino)benzenesulfonamide (11d)**

**

White solid; yield 24%. ^1^H NMR (600 MHz, DMSO-*d*_6_) δ 10.84 (s, 1H), 7.83 (dd, *J* = 7.8, 1.2 Hz, 1H), 7.65 (d, *J* = 2.4 Hz, 1H), 7.60 (s, 1H), 7.47 (t, *J* = 8.4 Hz, 1H), 7.40 – 7.37 (m, 2H), 7.37 – 7.35 (m, 1H), 7.35 – 7.32 (m, 1H), 7.31 (dd, *J* = 9.0, 2.4 Hz, 1H), 7.17 (d, *J* = 9.0 Hz, 1H), 7.12 (d, *J* = 8.4 Hz, 1H), 7.05 (d, *J* = 7.8 Hz, 1H). ^13^C NMR (151 MHz, DMSO-*d*_6_) δ 140.29, 138.15, 136.67, 134.77, 130.74, 130.22, 129.36, 127.98, 127.76, 126.66, 125.46, 124.90, 124.46, 122.92, 121.42, 120.68, 118.07, 117.63, 112.02. MS (ESI) (*m/z*): 416.56 (M-H)^-^. HRMS (ESI) calcd for C_19_H_13_Cl_2_N_3_O_2_S [M+H]^+^: 418.0178; found: 418.0182.

**^1^H NMR, ^13^C NMR, HRMS, and HPLC Spectra Data of Final Compounds.**


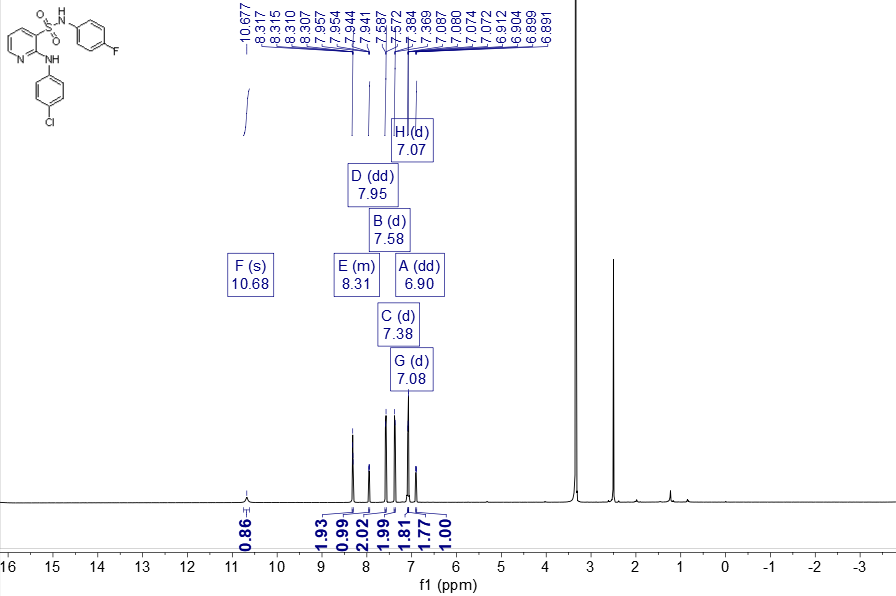


^1^H NMR of FBX-1


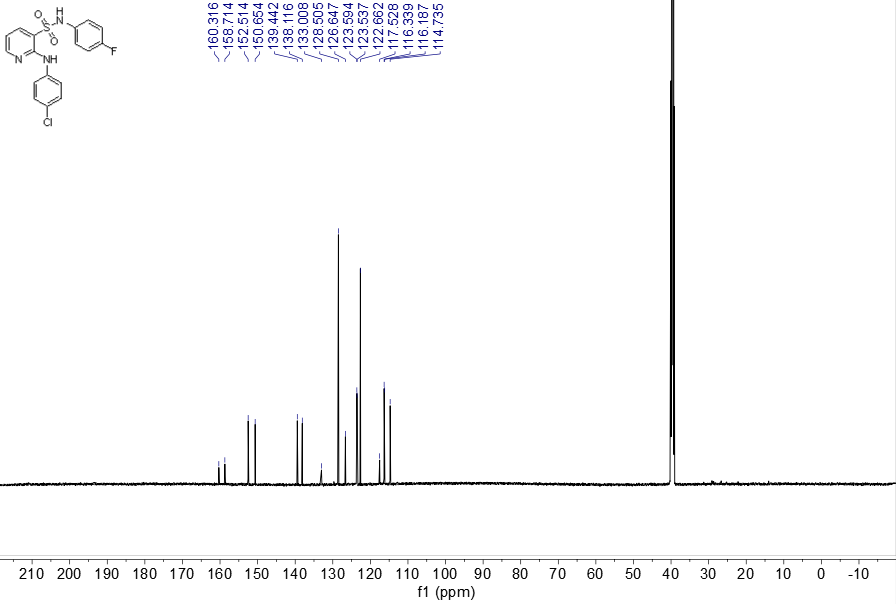


^13^C NMR of FBX-1


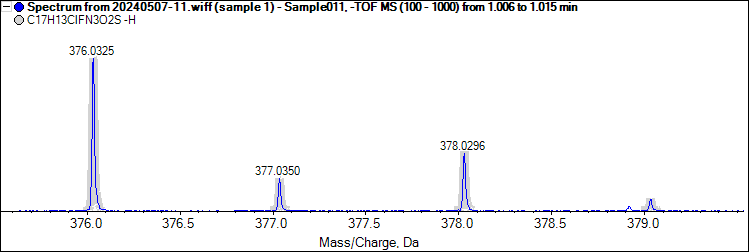


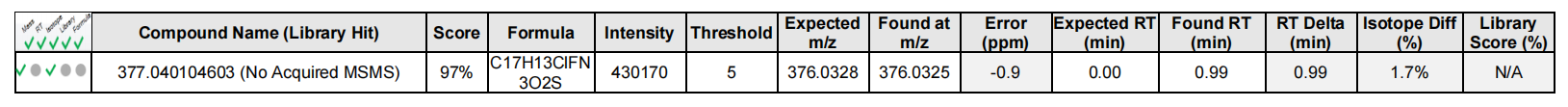


HRMS of FBX-1


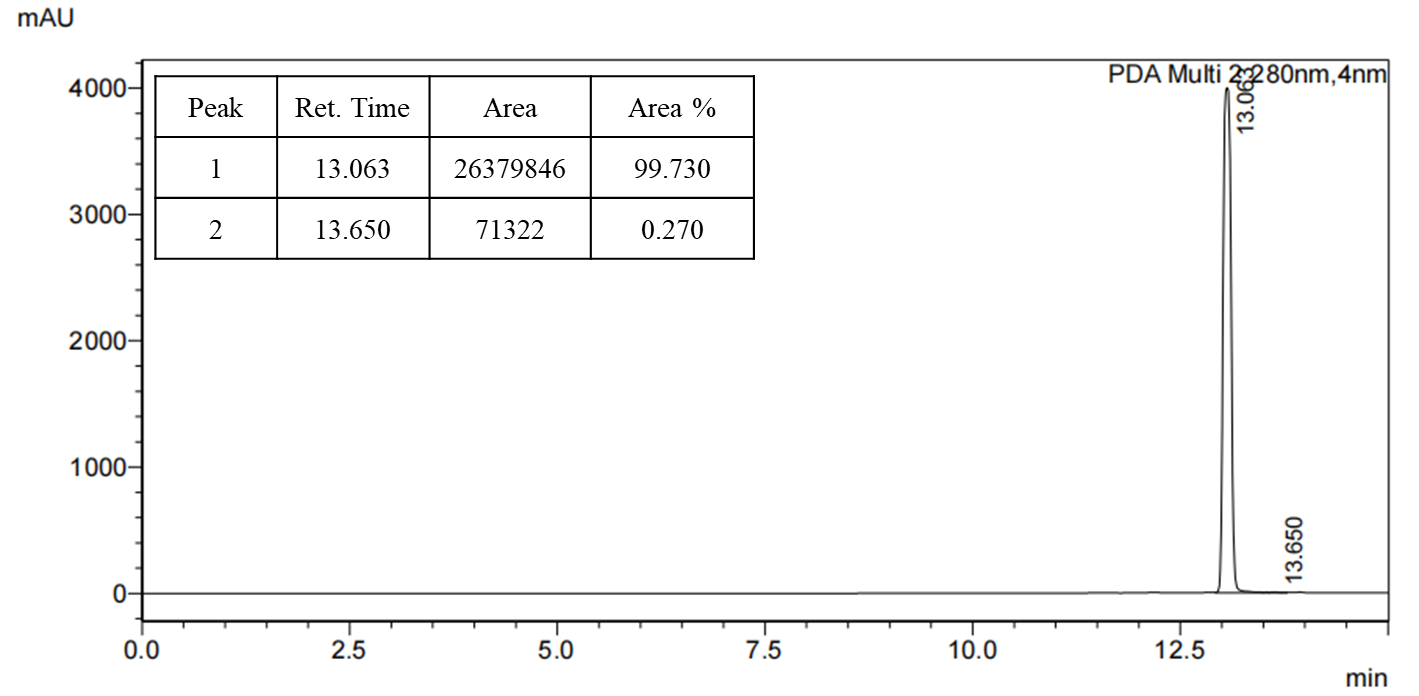


HPLC of FBX-1


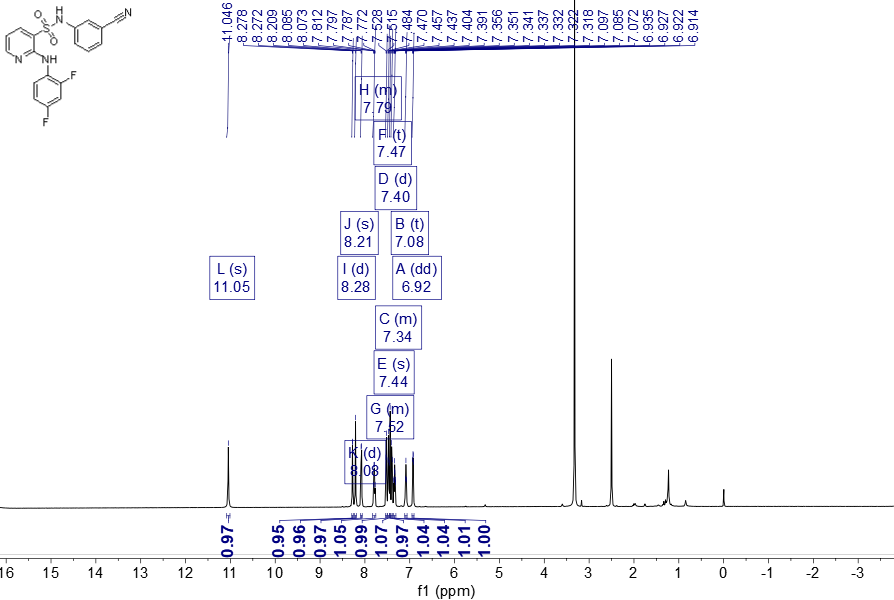


^1^H NMR of 8a


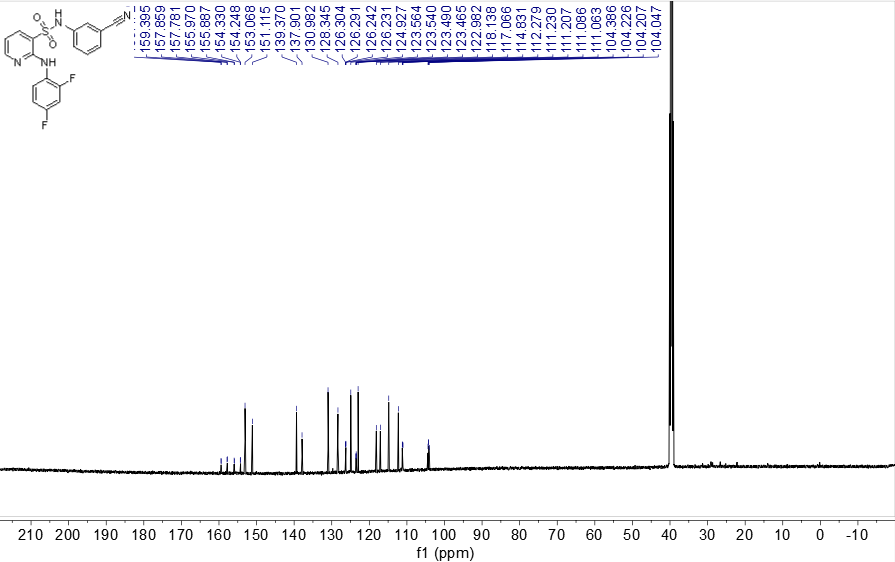


^13^C NMR of 8a


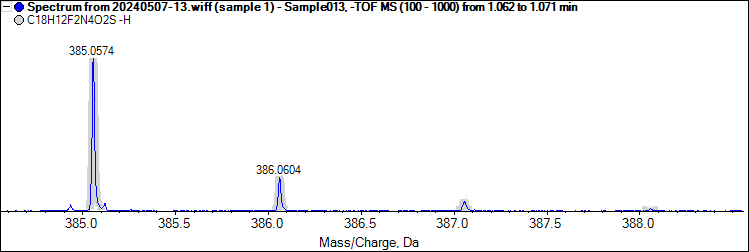


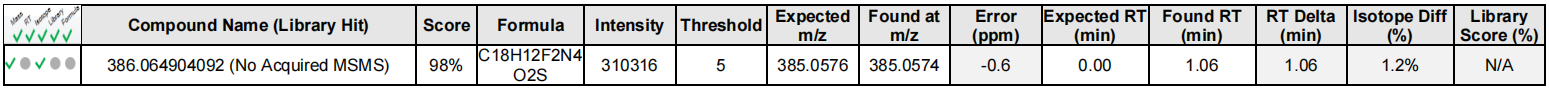


HRMS of 8a


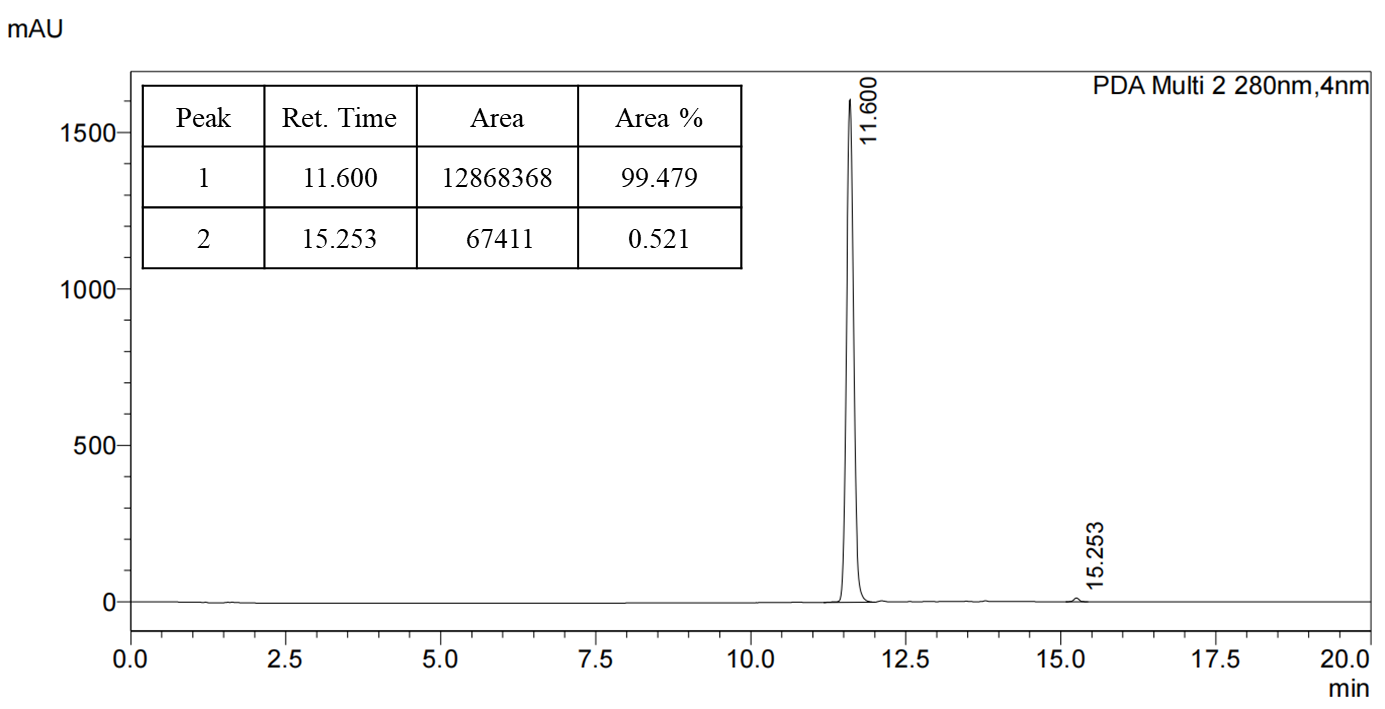


HPLC of 8a


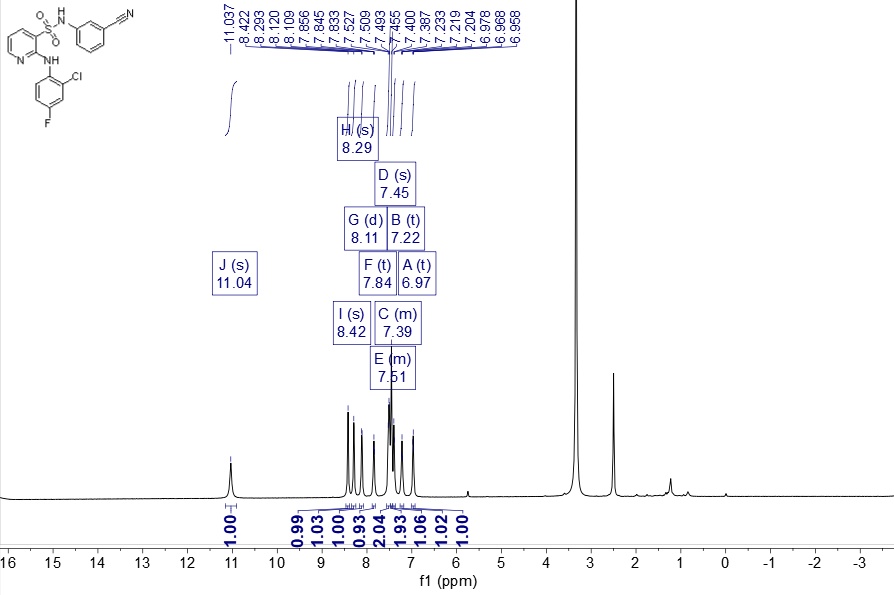


^1^H NMR of 8b


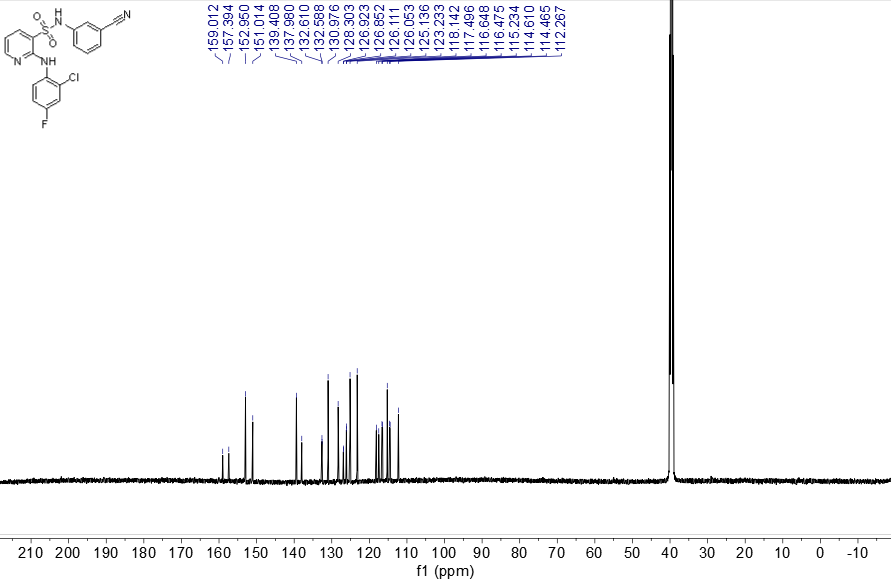


^13^C NMR of 8b


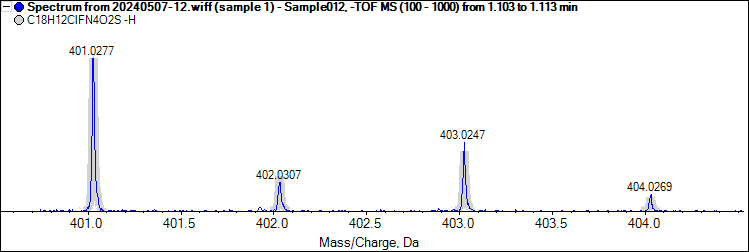


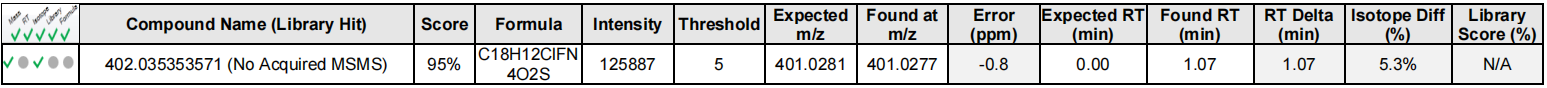


HRMS of 8b


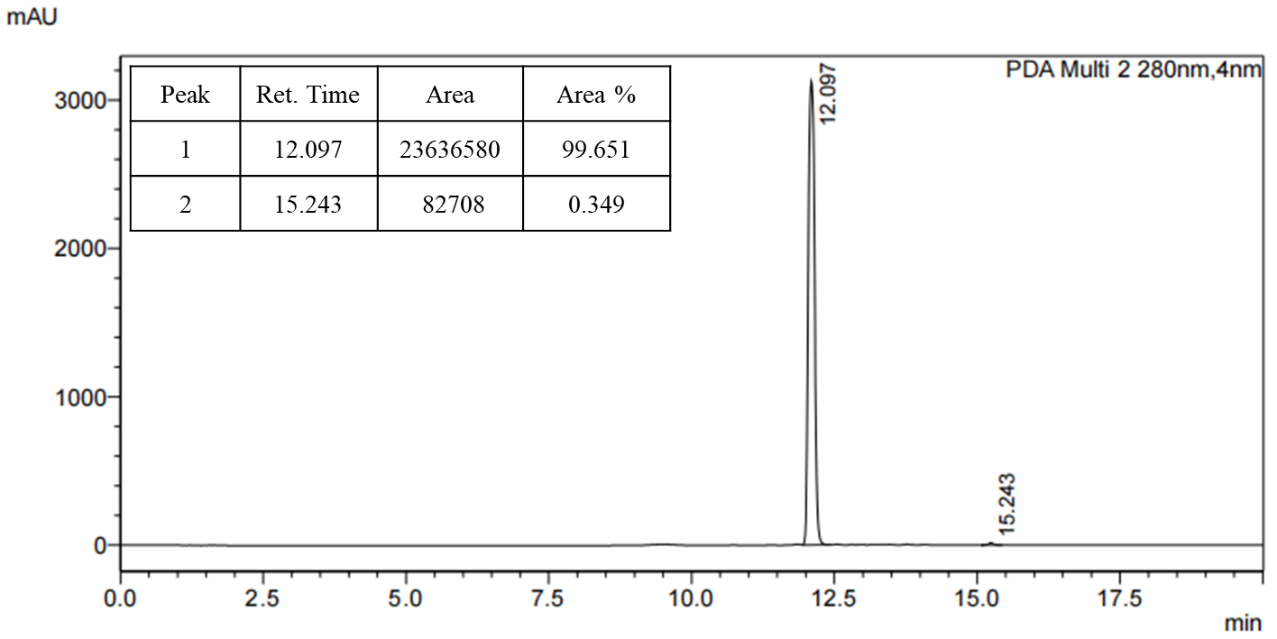


HPLC of 8b


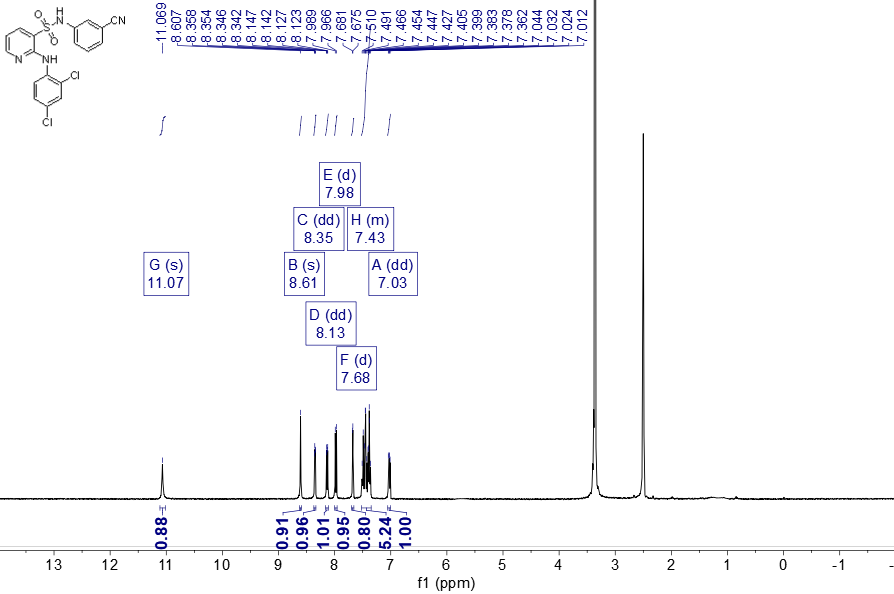


^1^H NMR of 8c


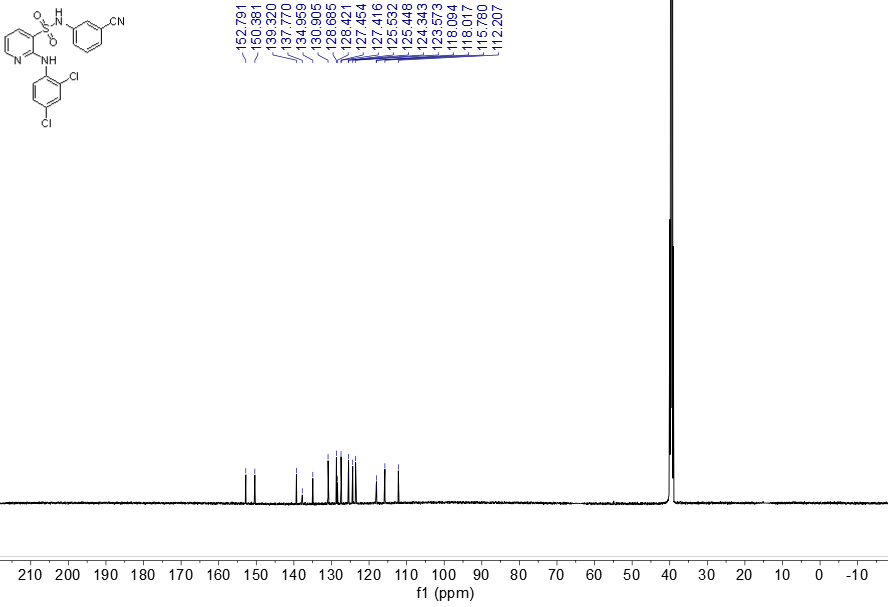


^13^C NMR of 8c


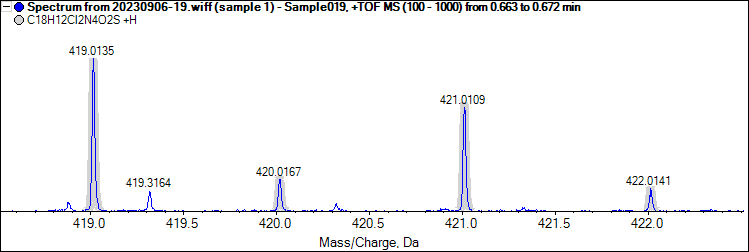


HRMS of 8c


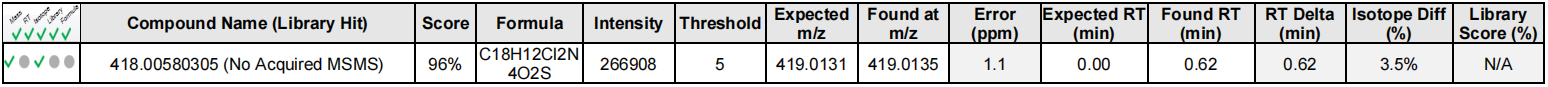


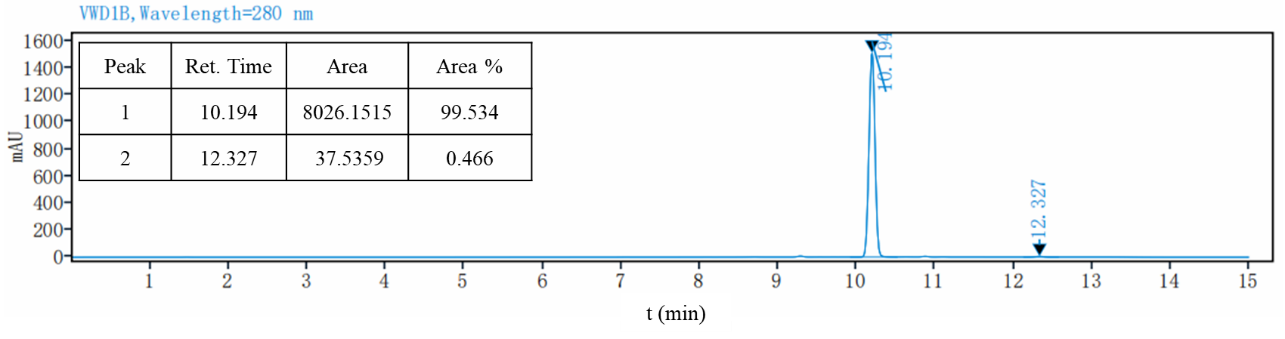


HPLC of 8c


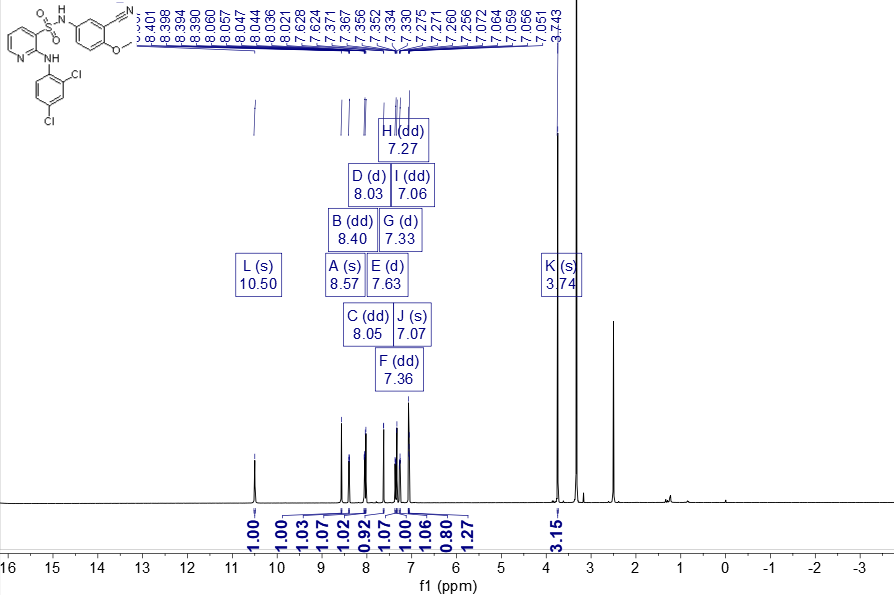


^1^H NMR of 8d


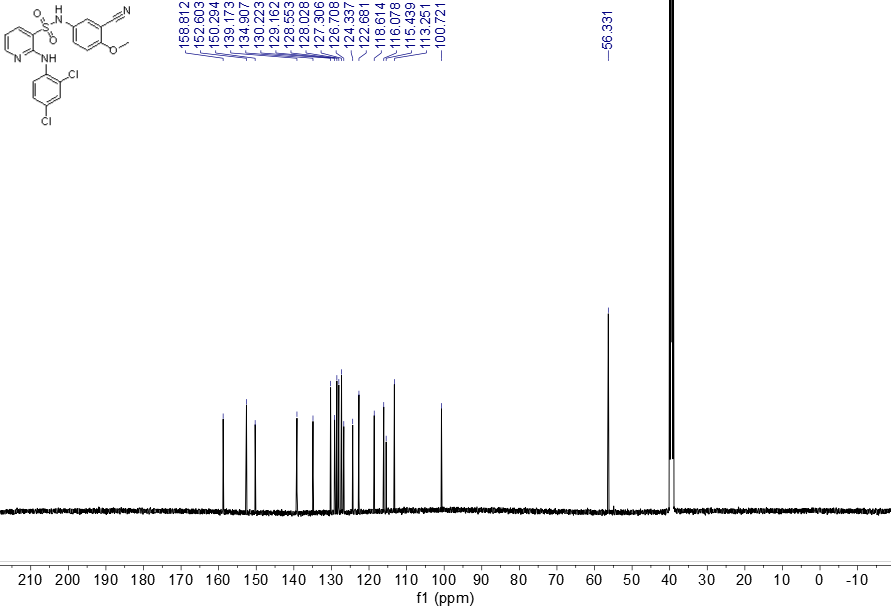


^13^C NMR of 8d


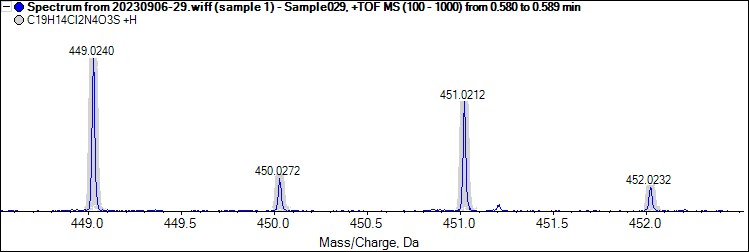


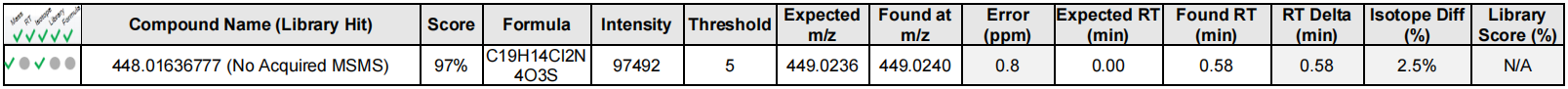


HRMS of 8d


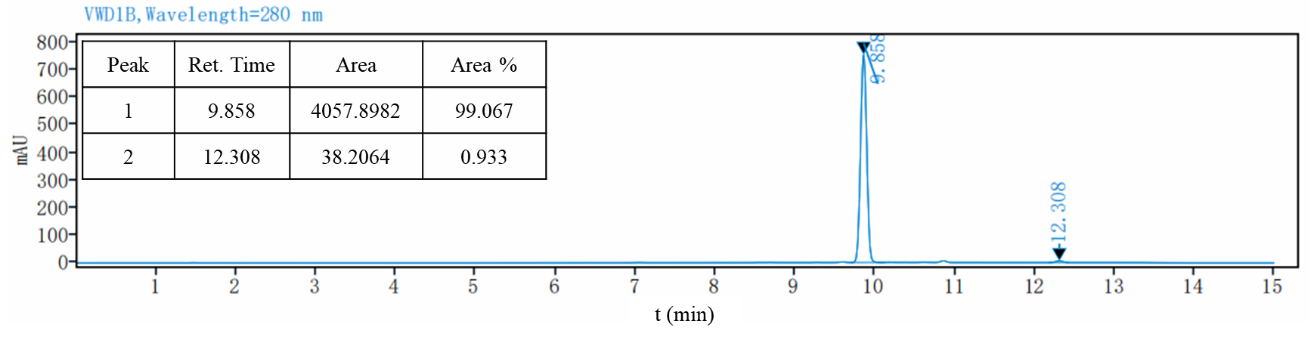


HPLC of 8d


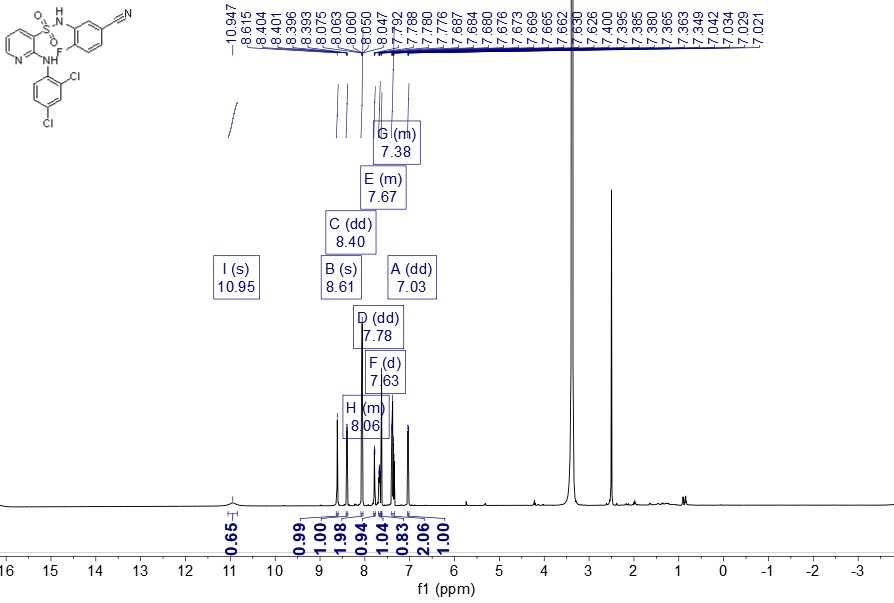


^1^H NMR of 8e


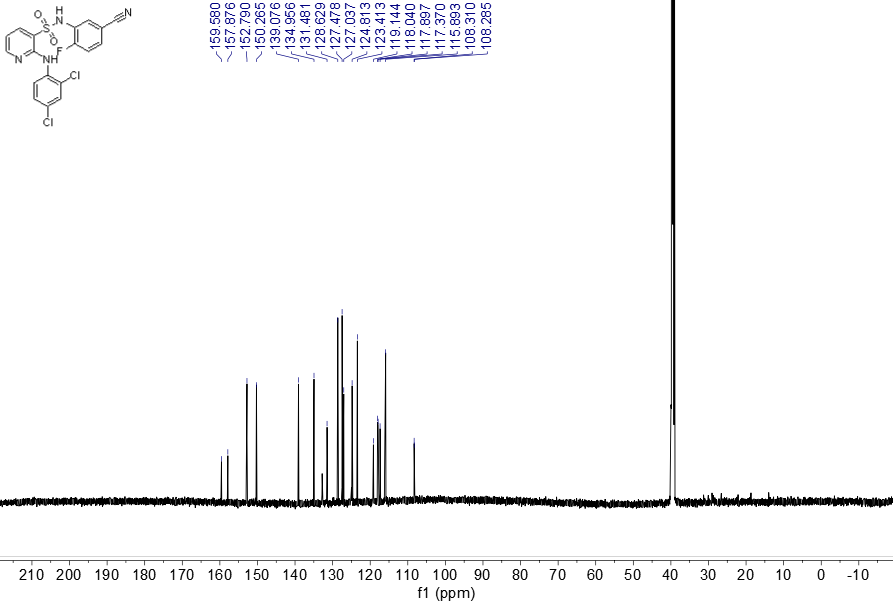


^13^C NMR of 8e


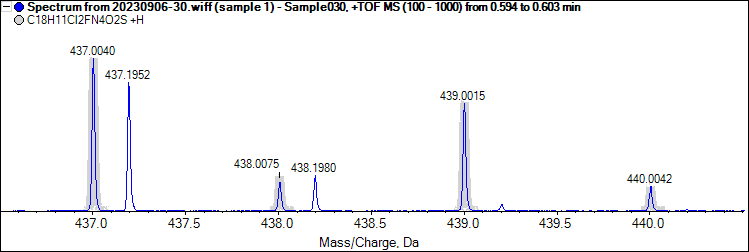


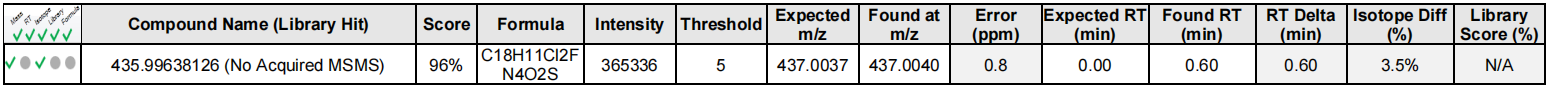


HRMS of 8e


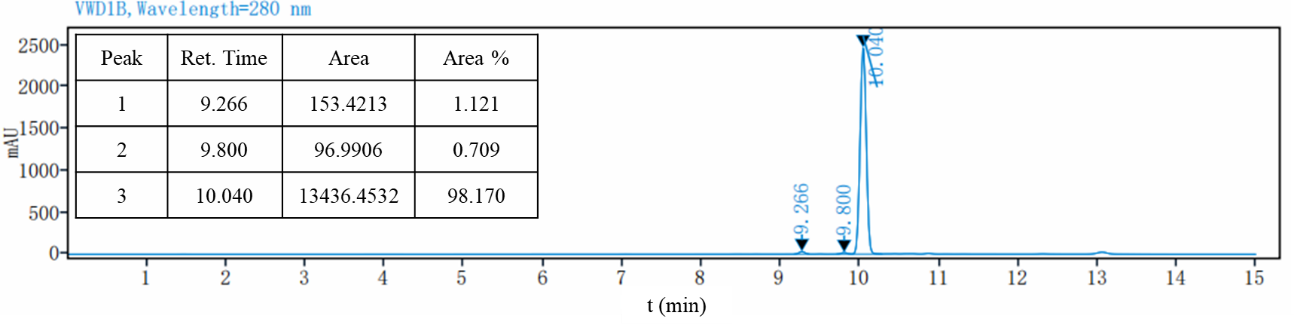


HPLC of 8e


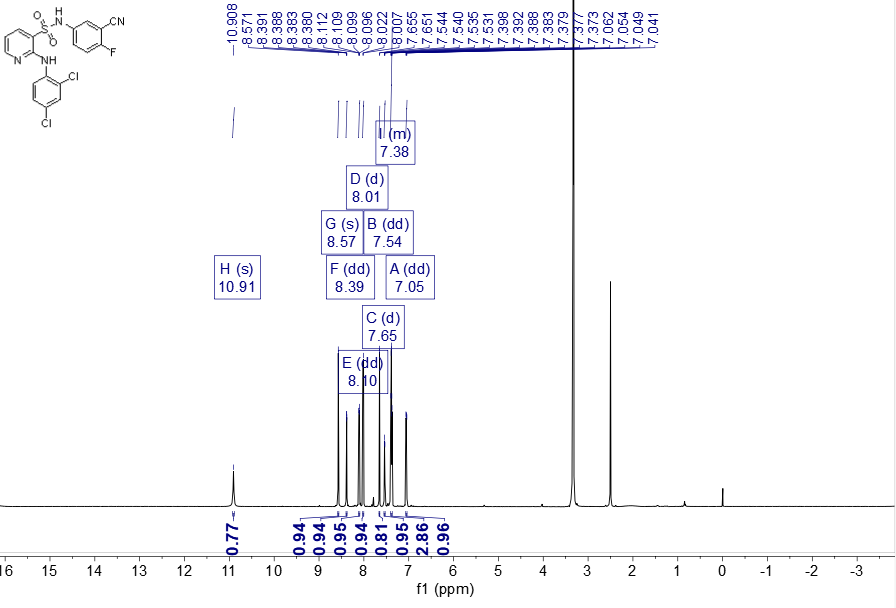


^1^H NMR of 8f


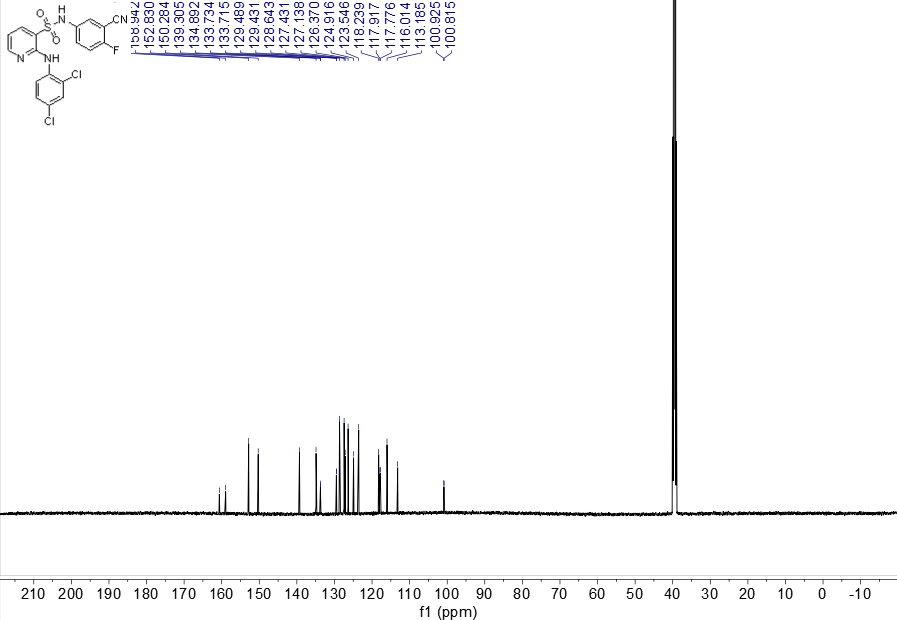


^13^C NMR of 8f


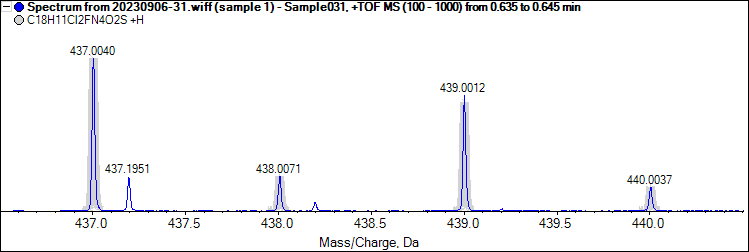


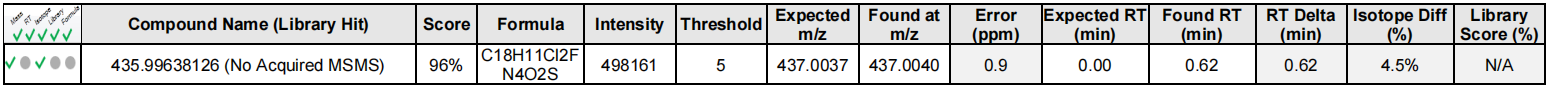


HRMS of 8f


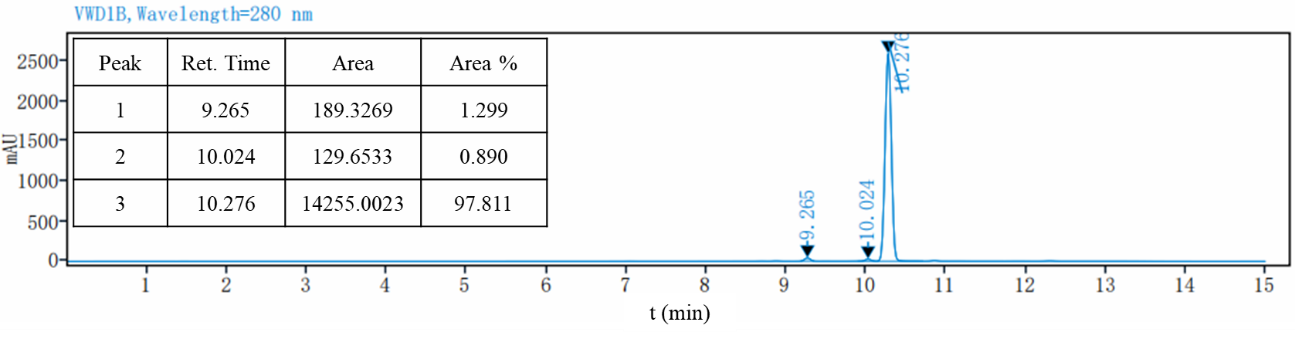


HPLC of 8f


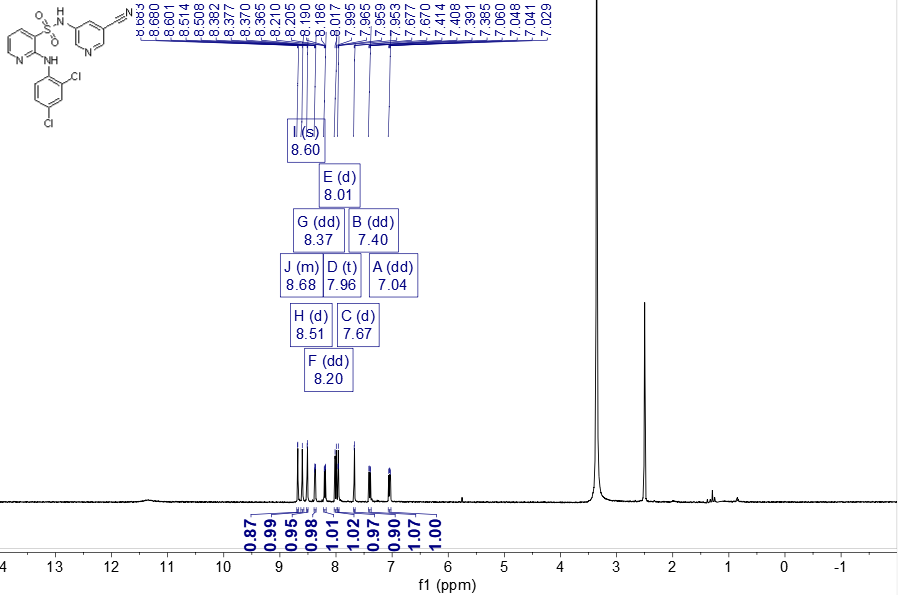


^1^H NMR of 8g


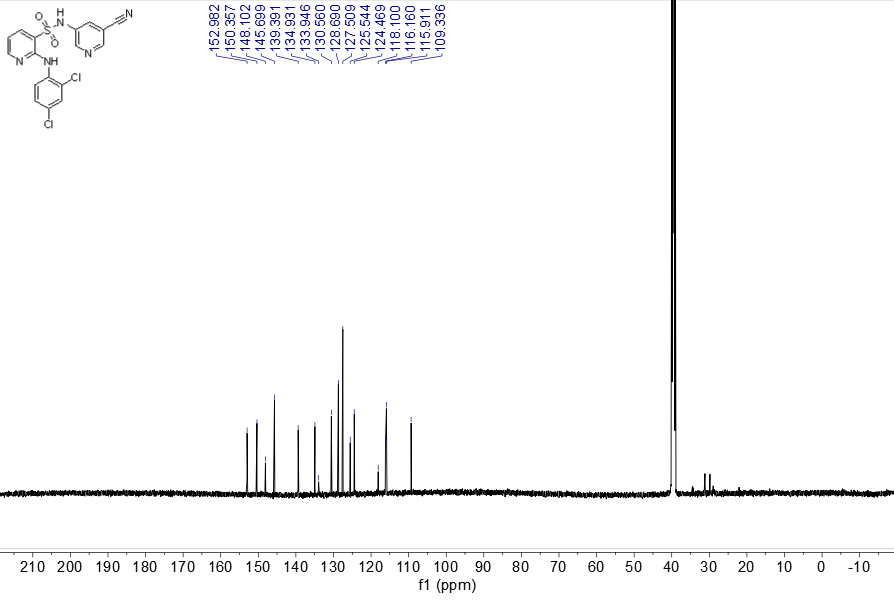


^13^C NMR of 8g


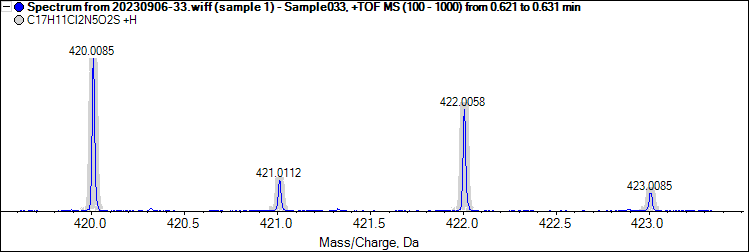


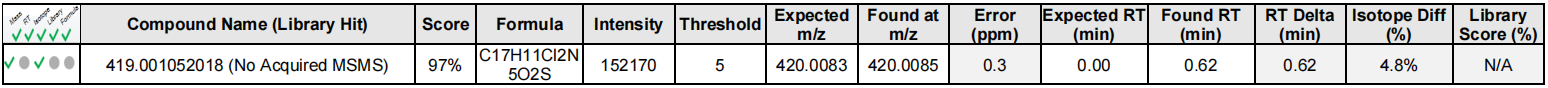


HRMS of 8g


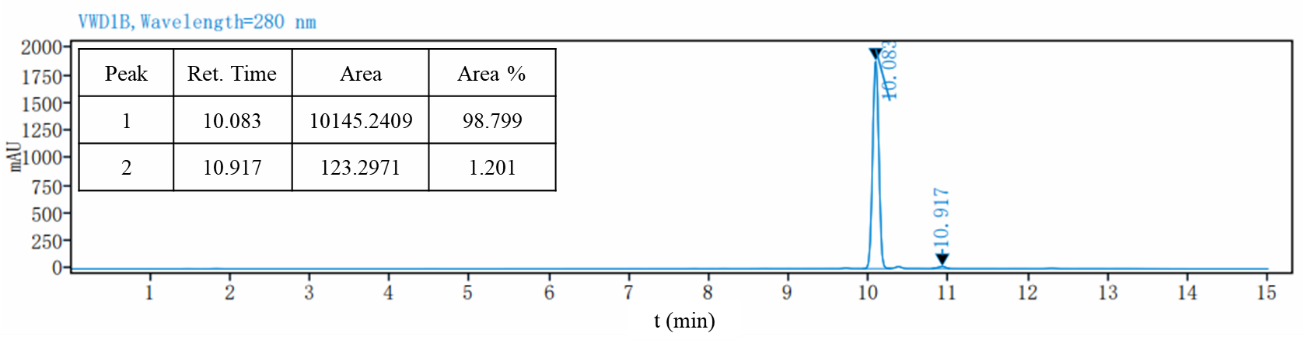


HPLC of 8g


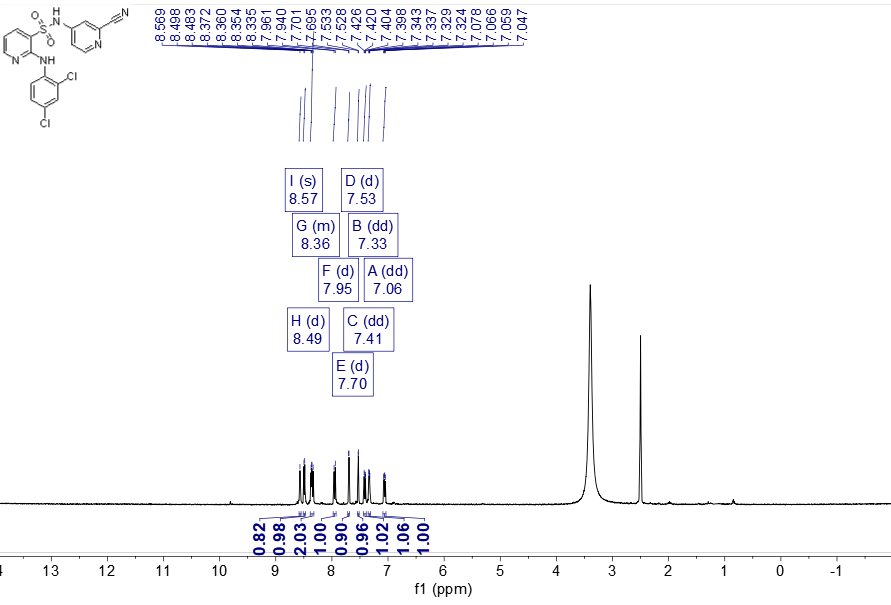


^1^H NMR of 8h


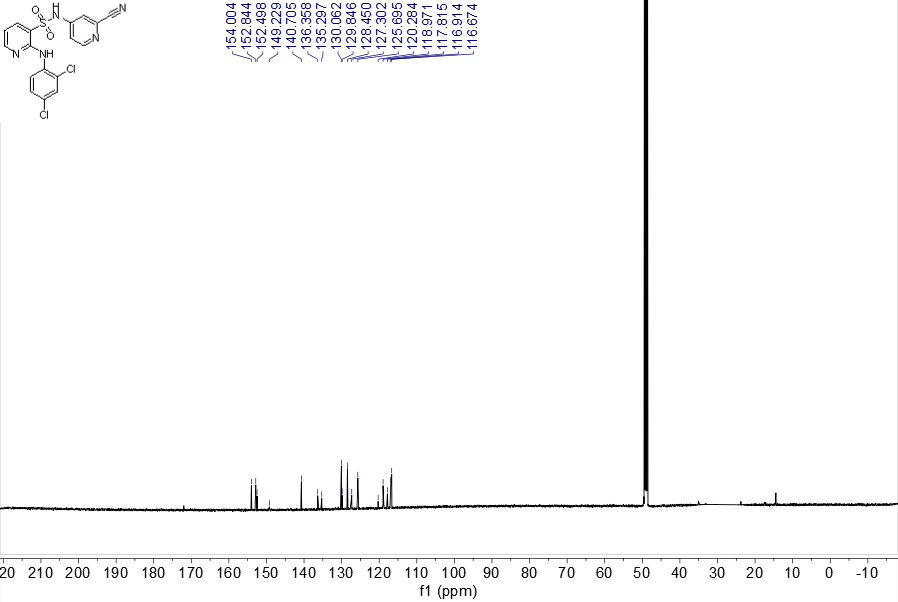


^13^C NMR of 8h


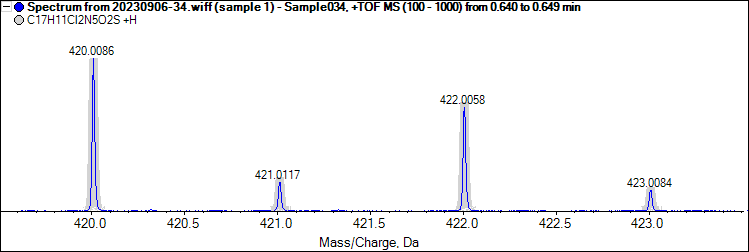


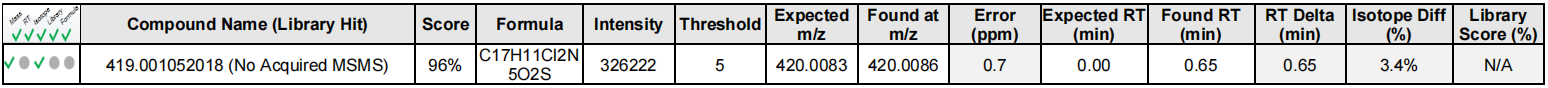


HRMS of 8h


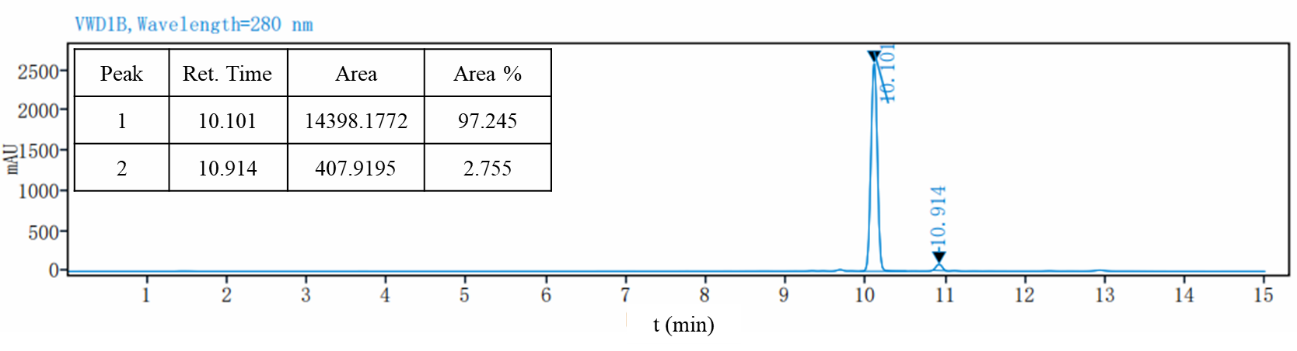


HPLC of 8h


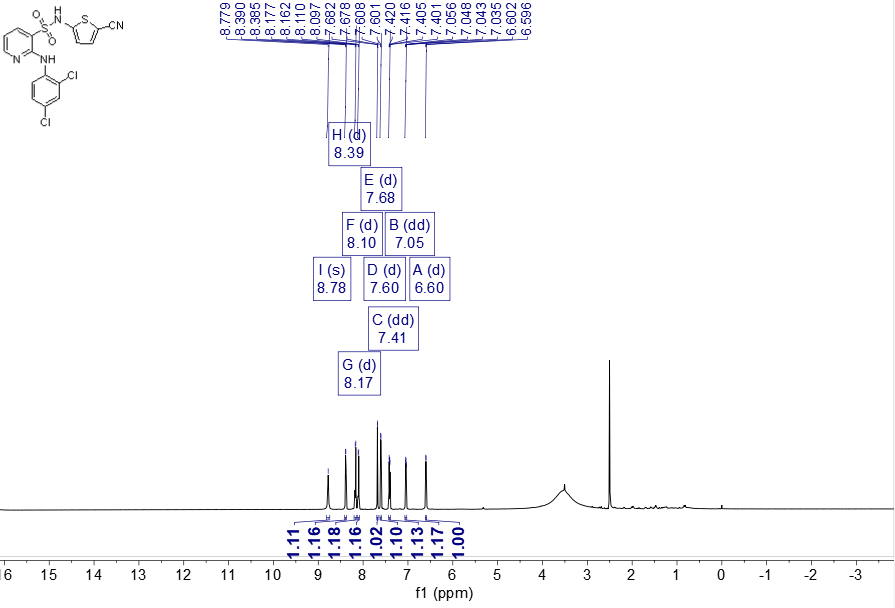


^1^H NMR of 8i


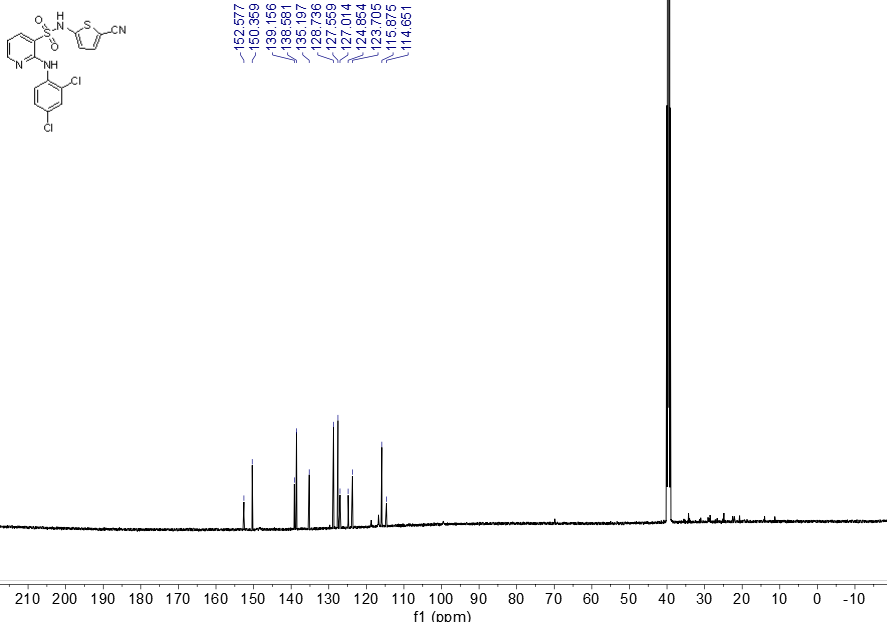


^13^C NMR of 8i


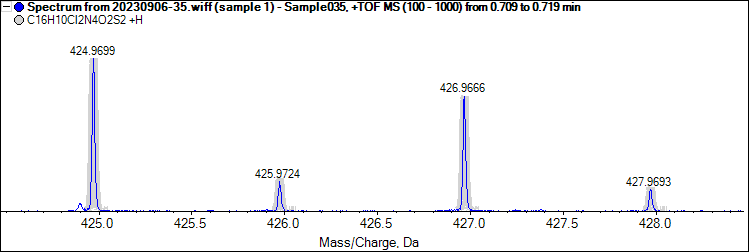


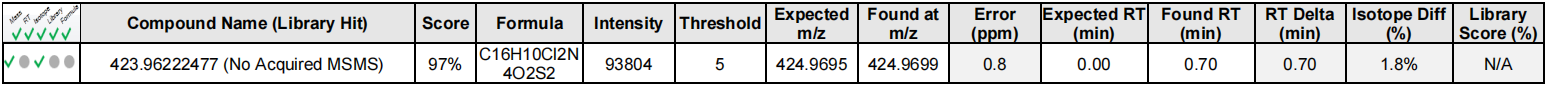


HRMS of 8i


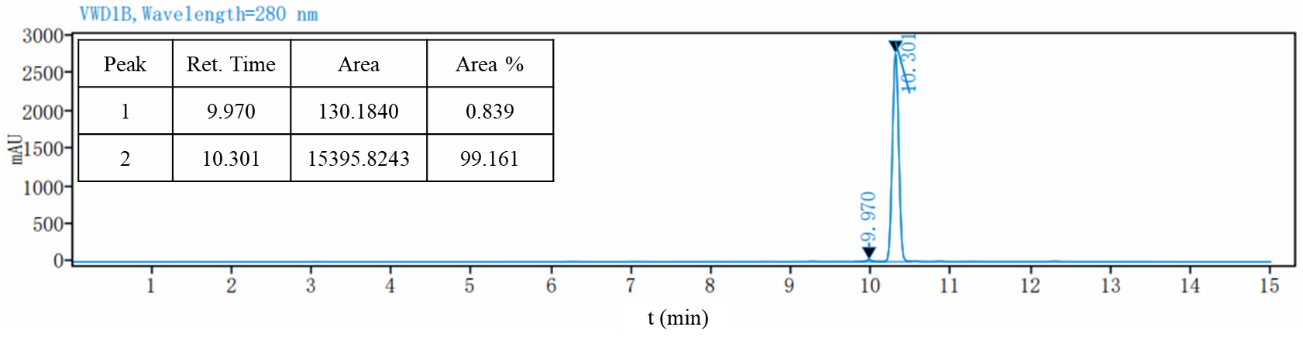


HPLC of 8i


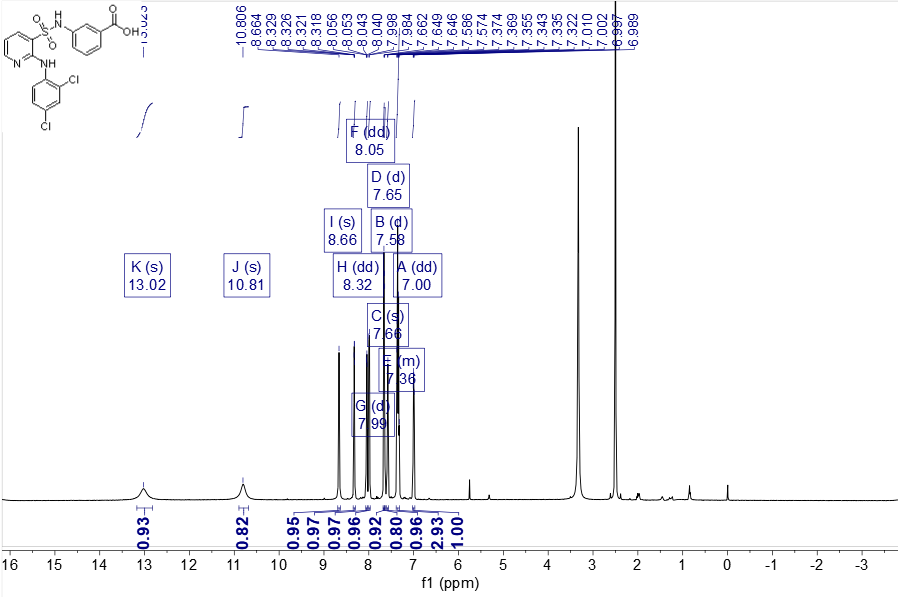


^1^H NMR of 8j


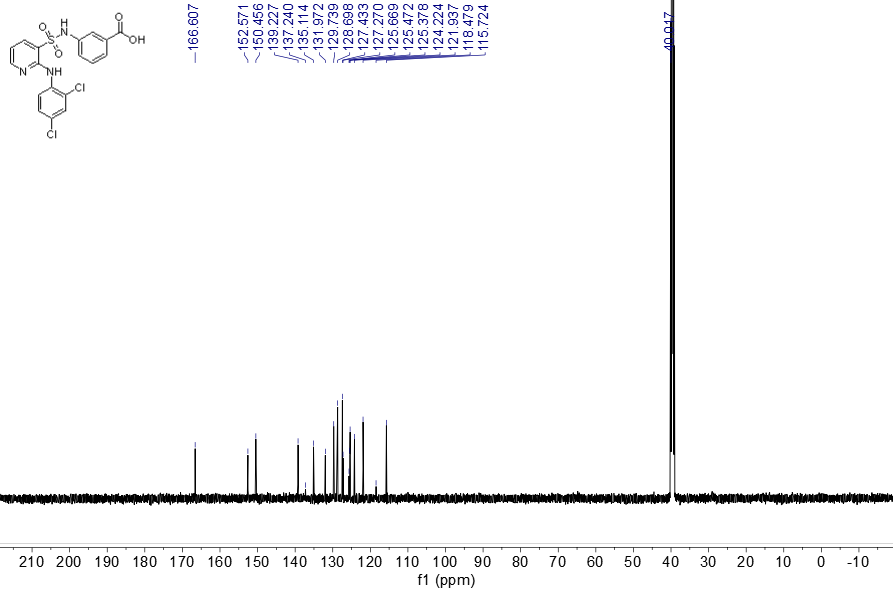


^13^C NMR of 8j


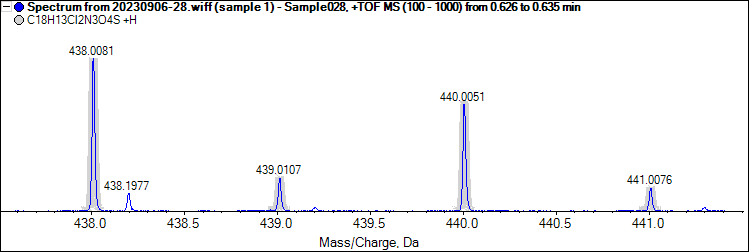


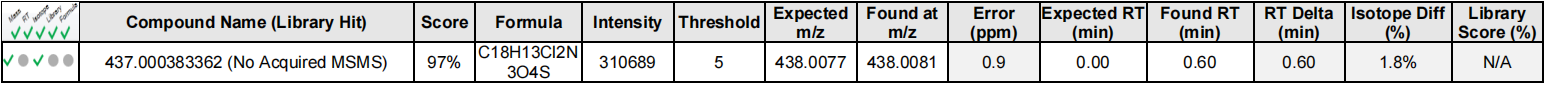


HRMS of 8j


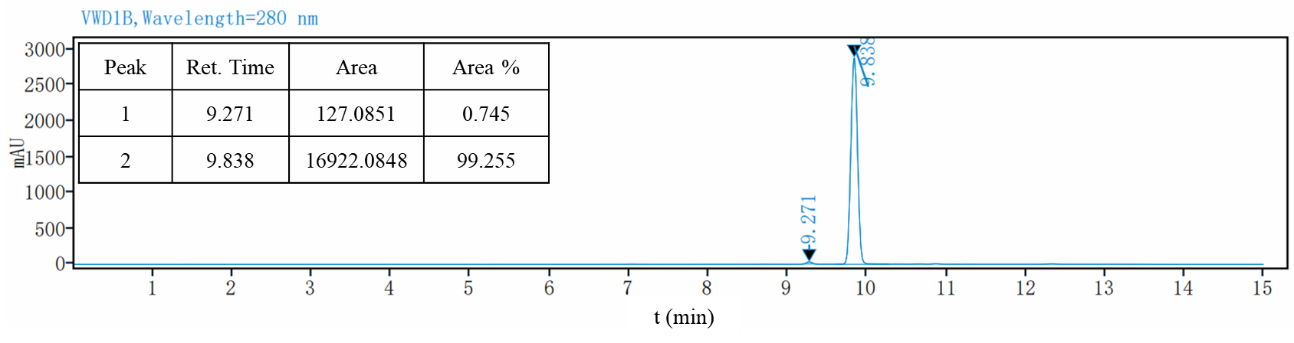


HPLC of 8j


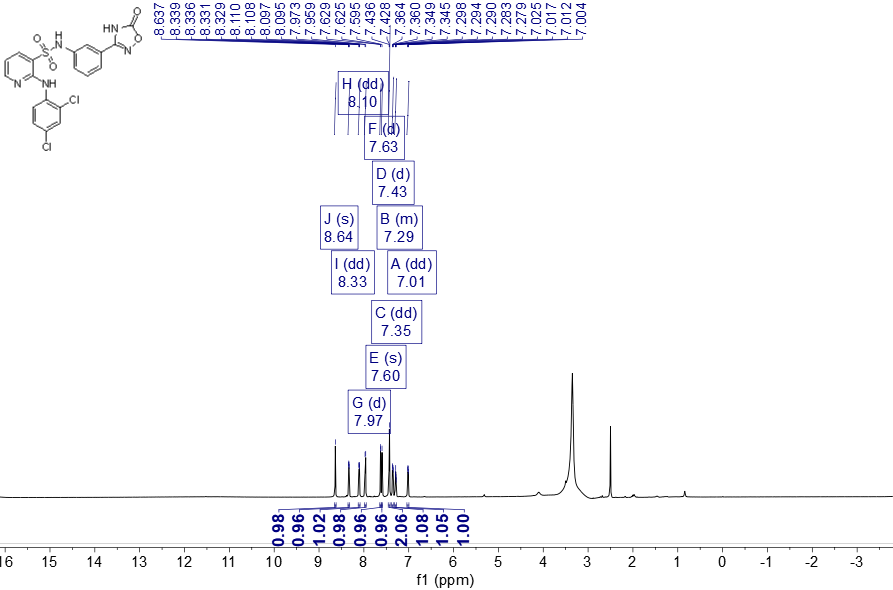


^1^H NMR of 8k


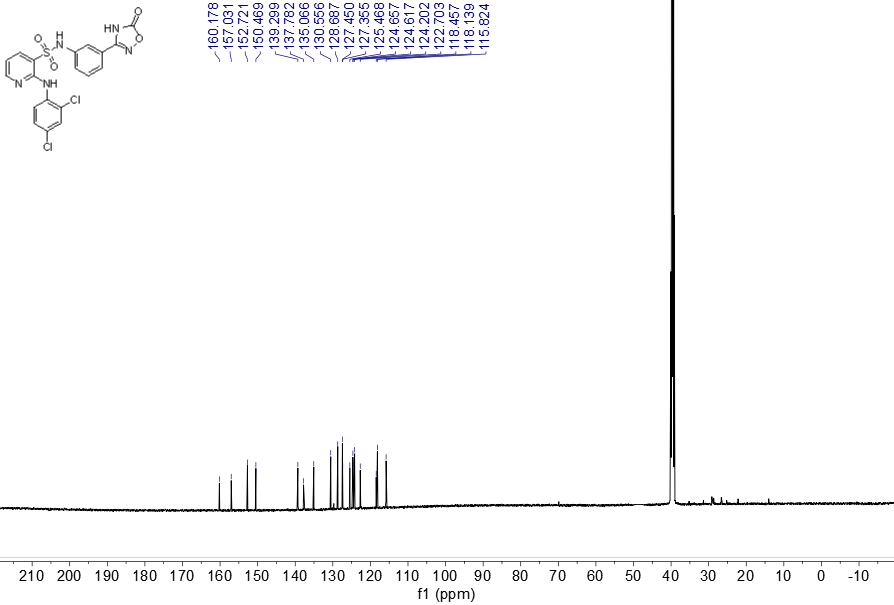


^13^C NMR of 8k


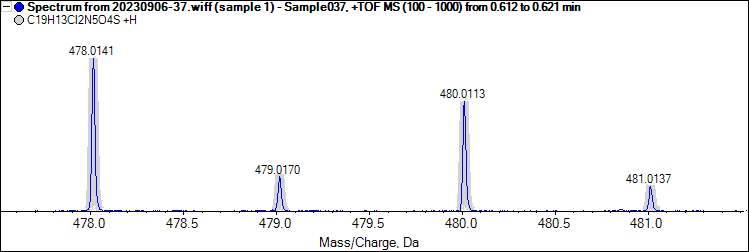


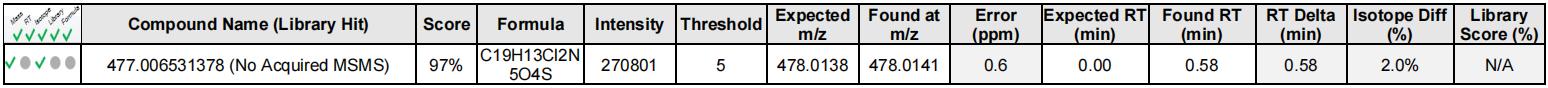


HRMS of 8k


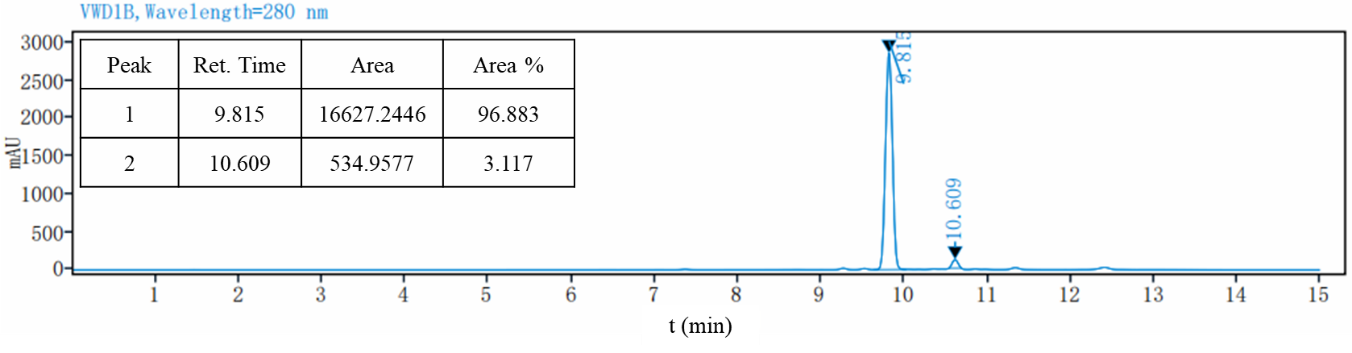


HPLC of 8k


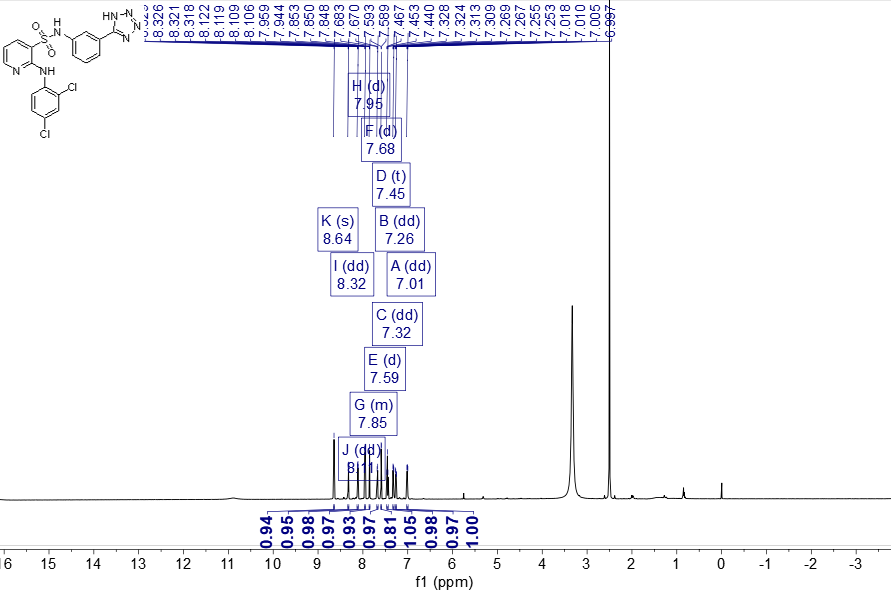


^1^H NMR of 8l


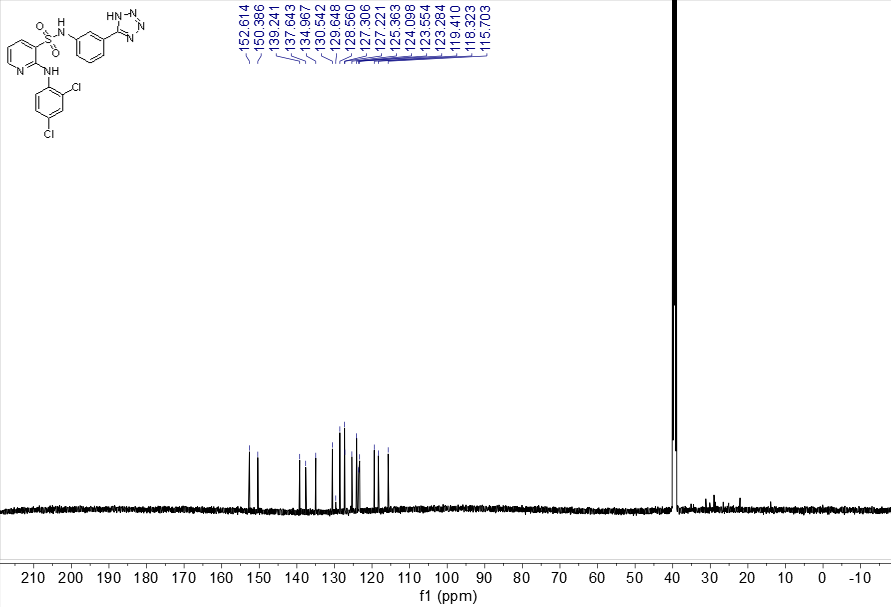


^13^C NMR of 8l


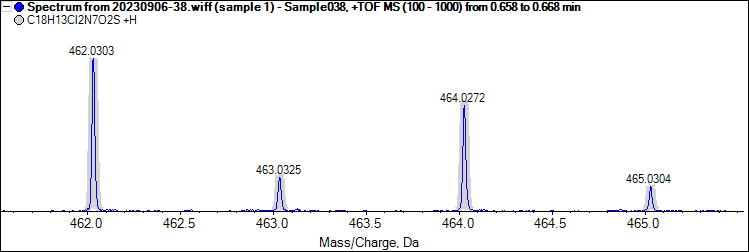


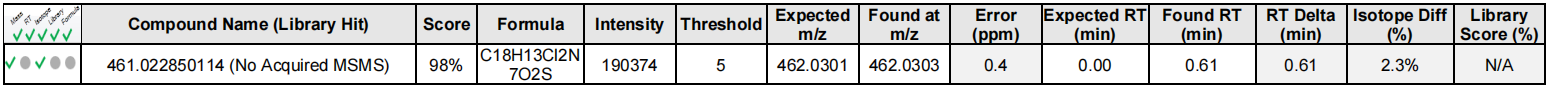


HRMS of 8l


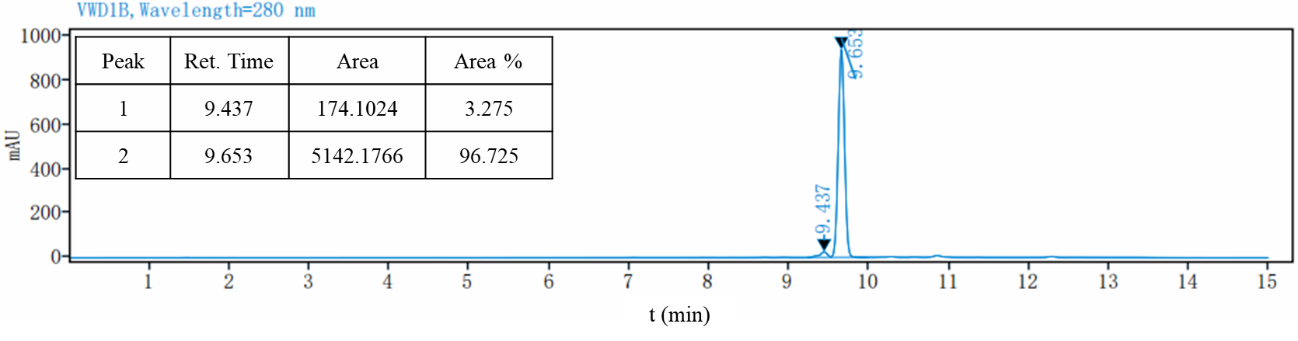


HPLC of 8l


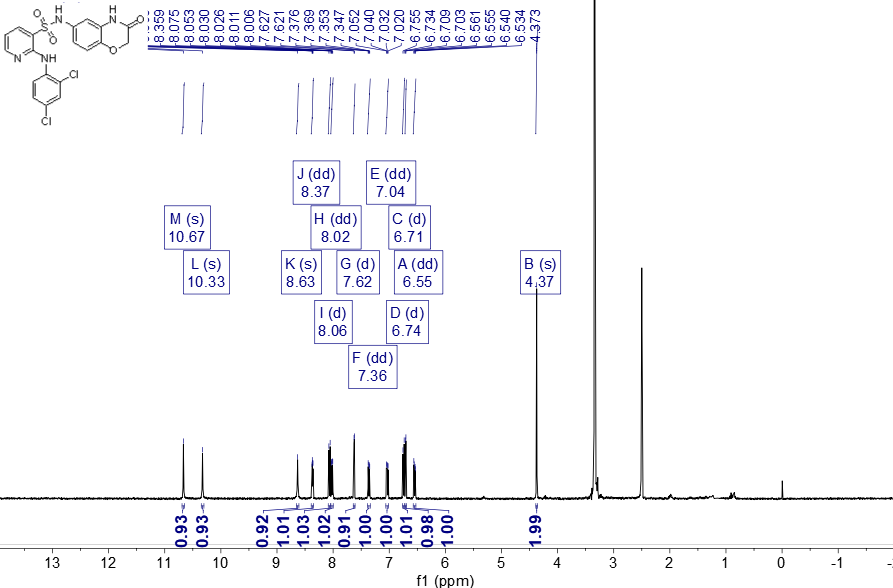


^1^H NMR of 8m


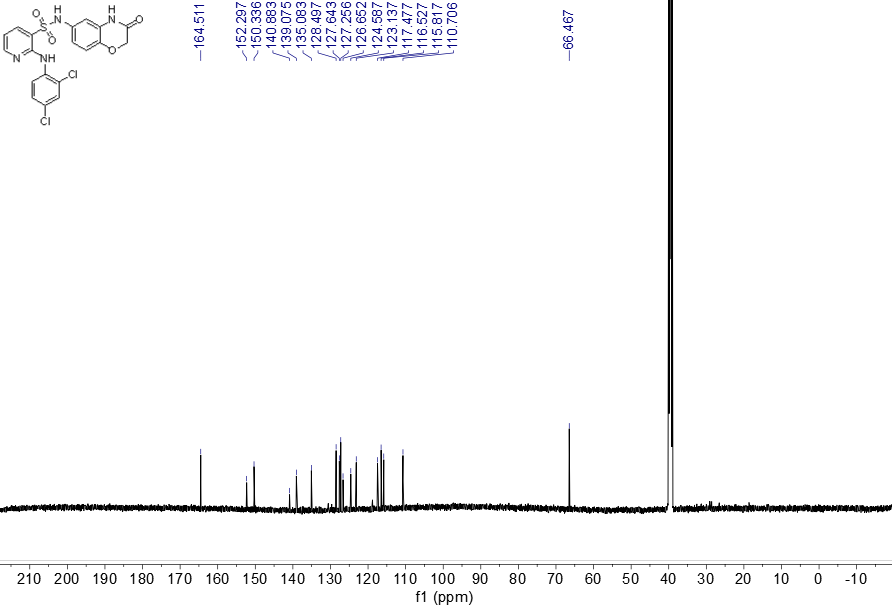


^13^C NMR of 8m


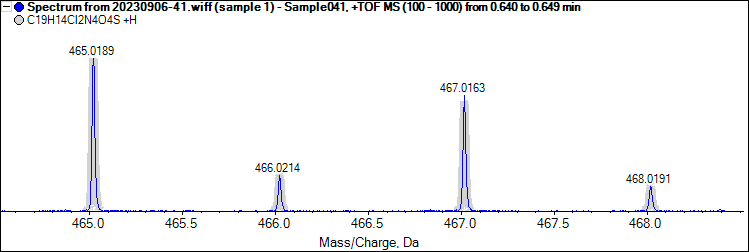


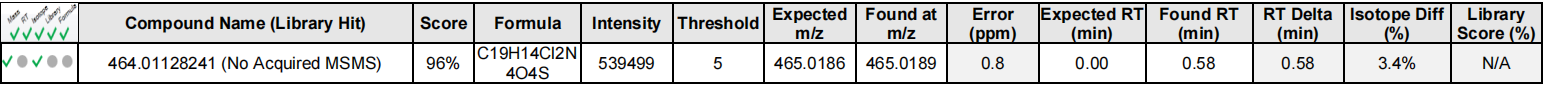


HRMS of 8m


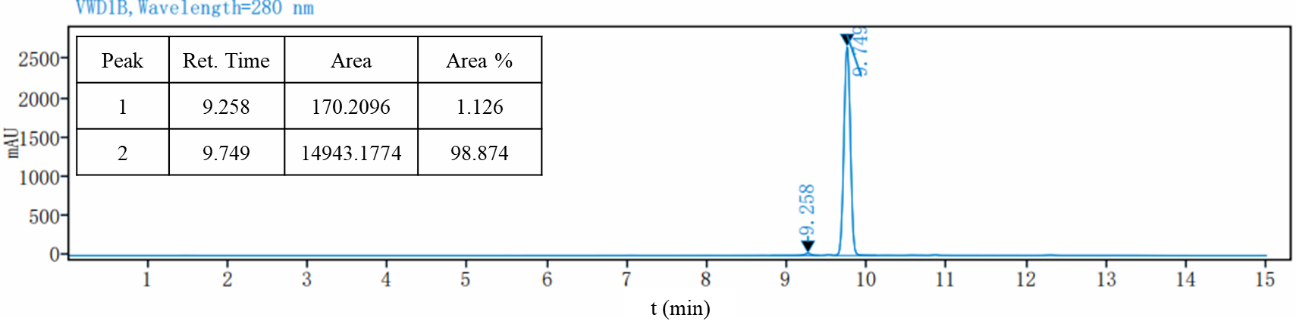


HPLC of 8m


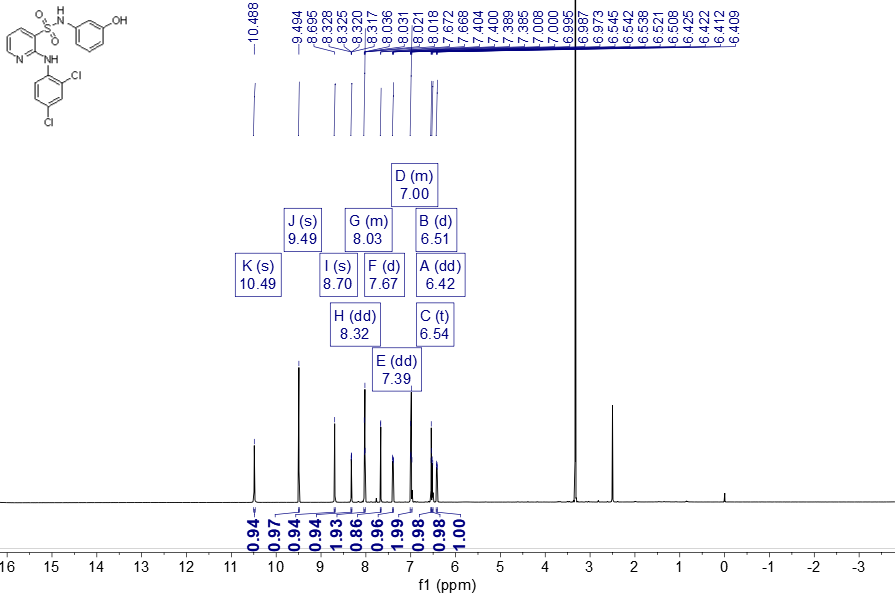


^1^H NMR of 8n


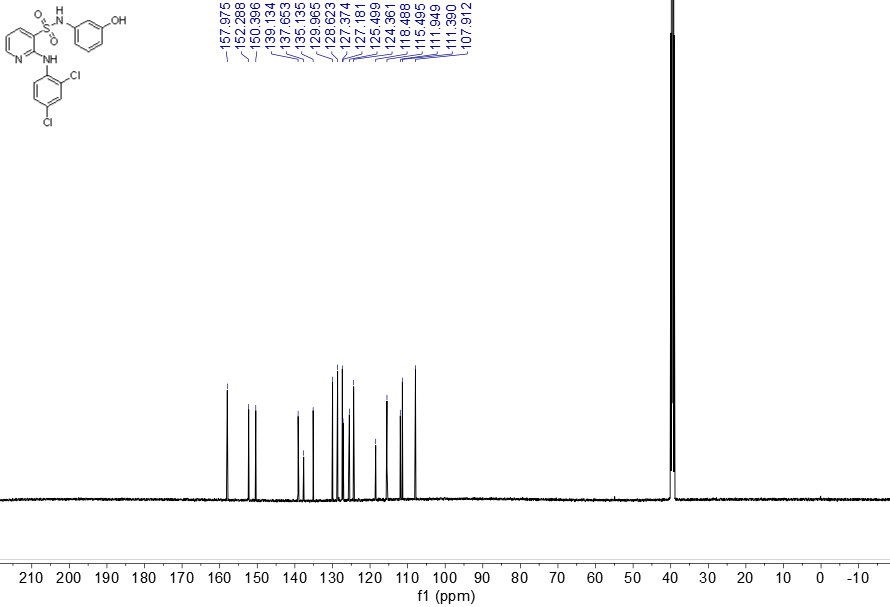


^13^C NMR of 8n


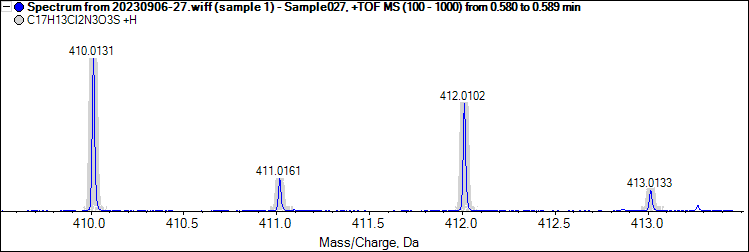


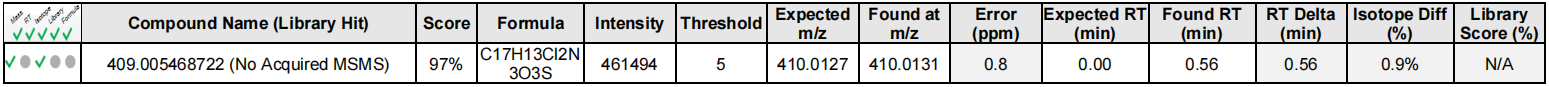


HRMS of 8n


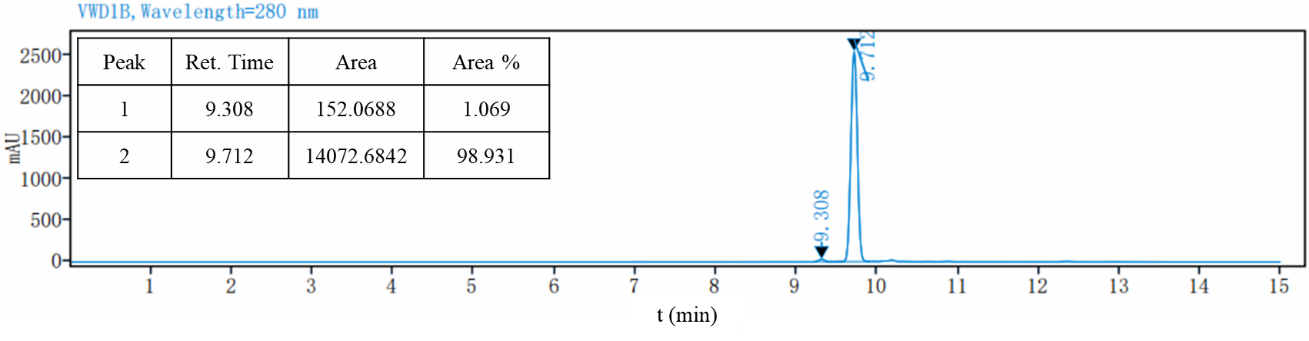


HPLC of 8n


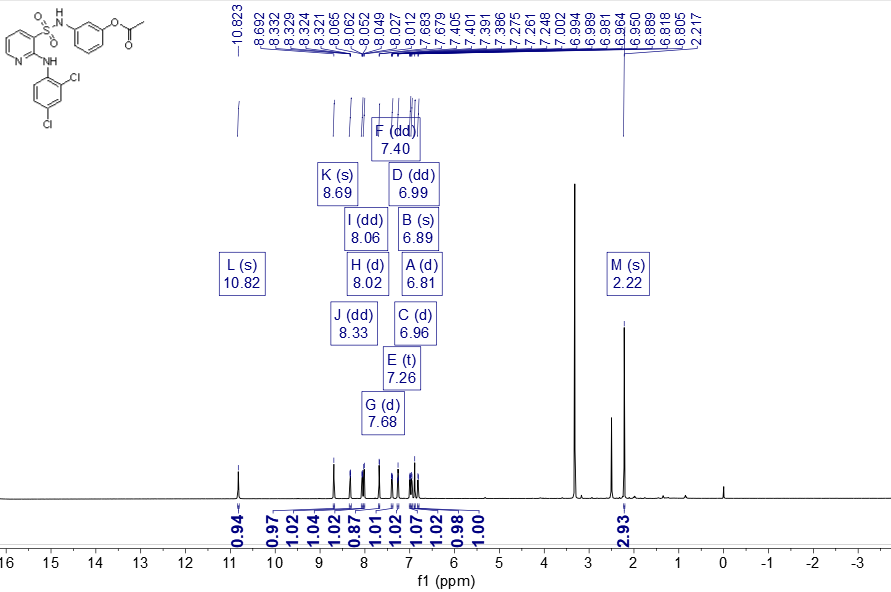


^1^H NMR of 8o


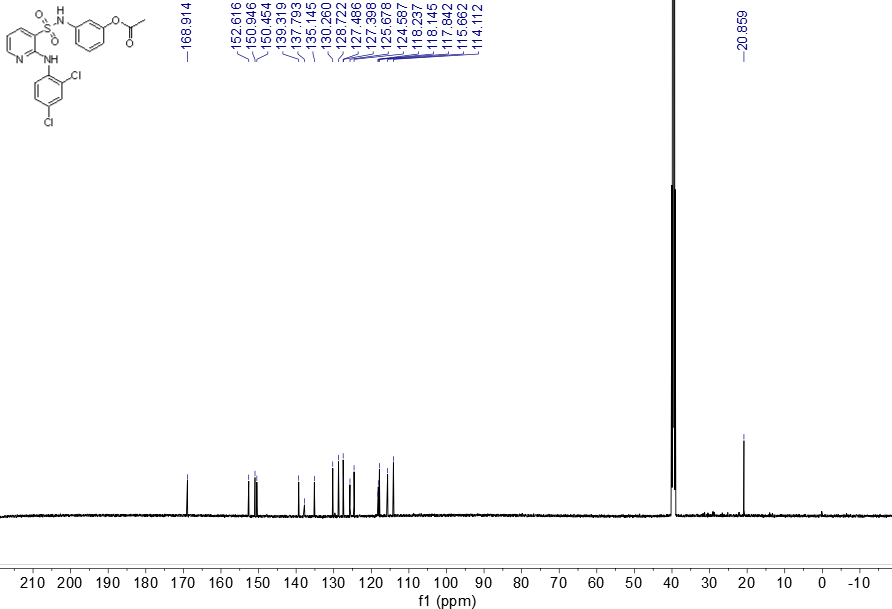


^13^C NMR of 8o


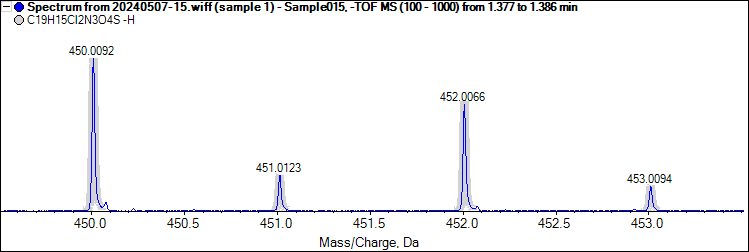


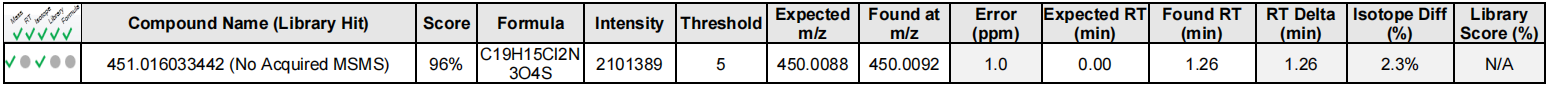


HRMS of 8o


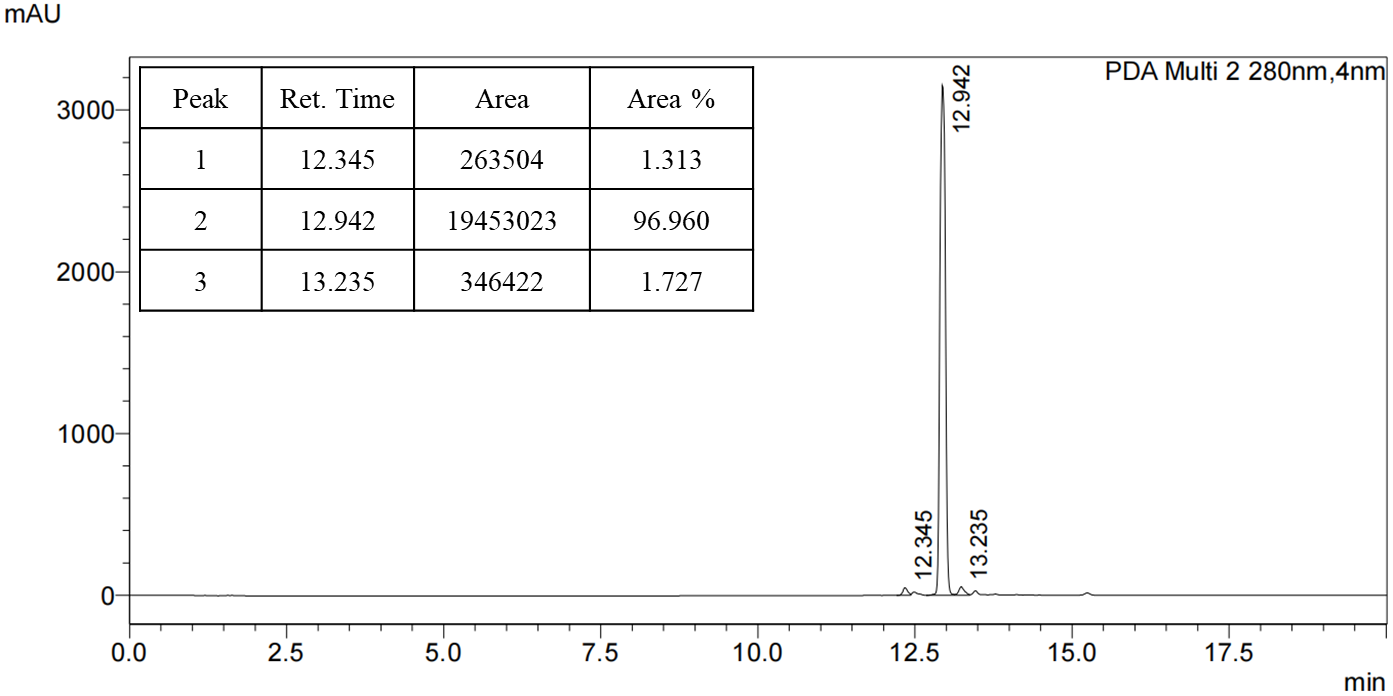


HPLC of 8o


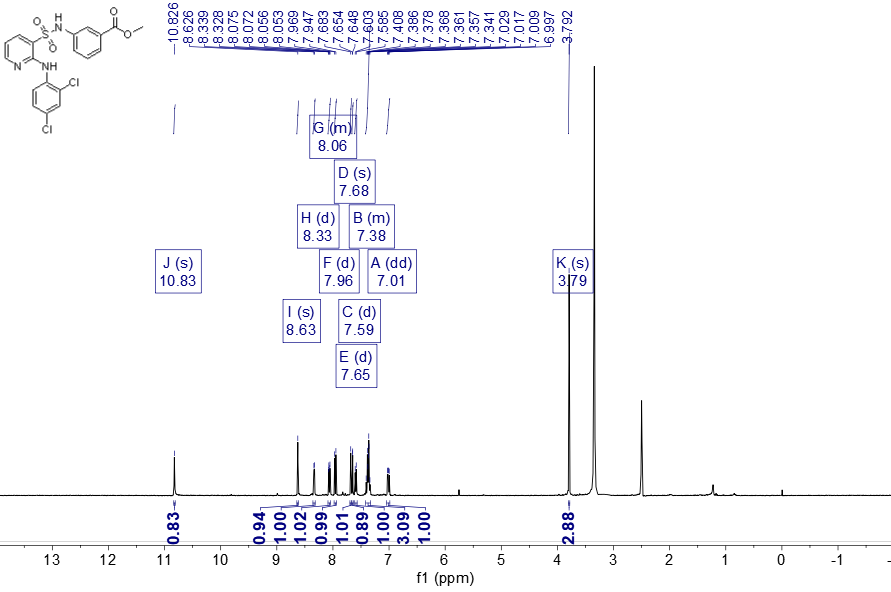


^1^H NMR of 8p


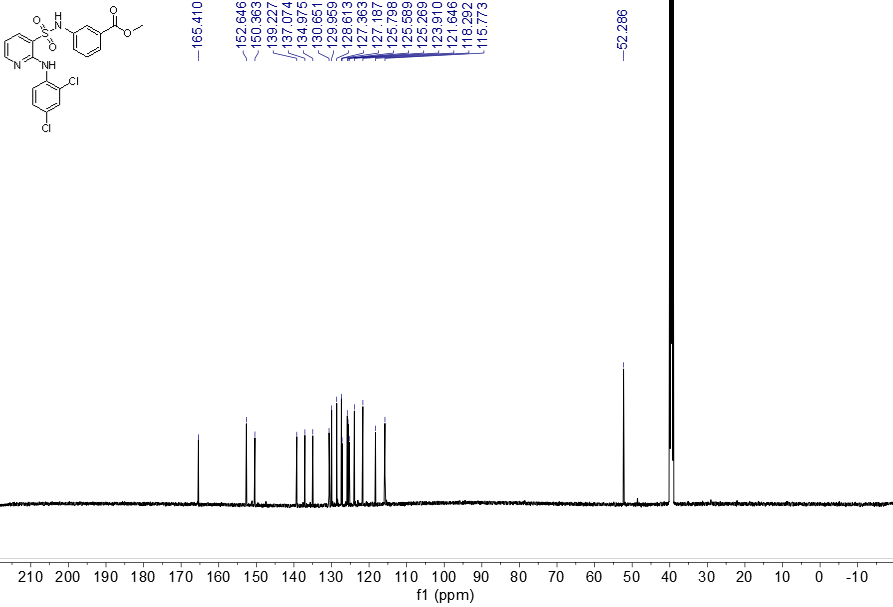


^13^C NMR of 8p


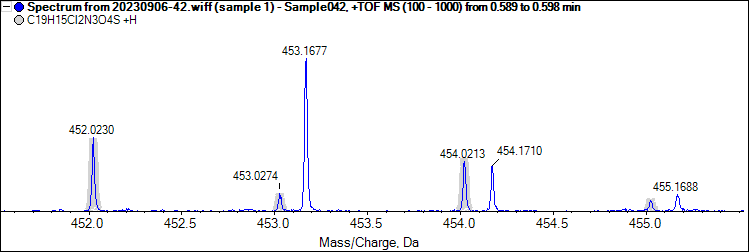


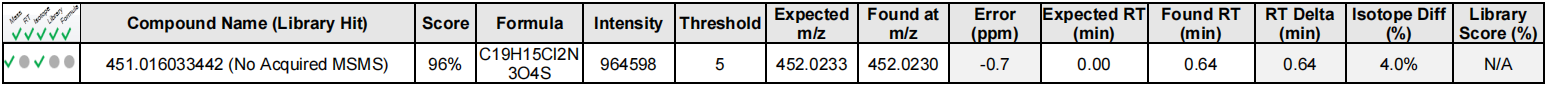


HRMS of 8p


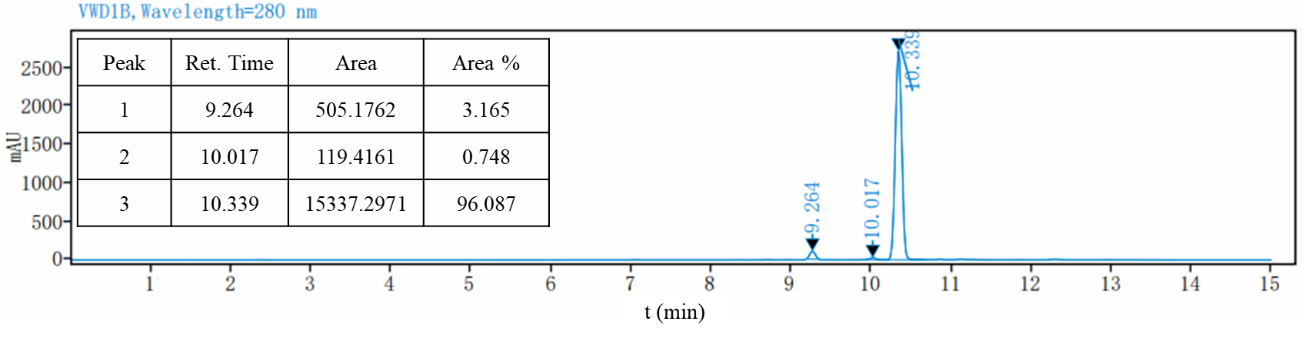


HPLC of 8p


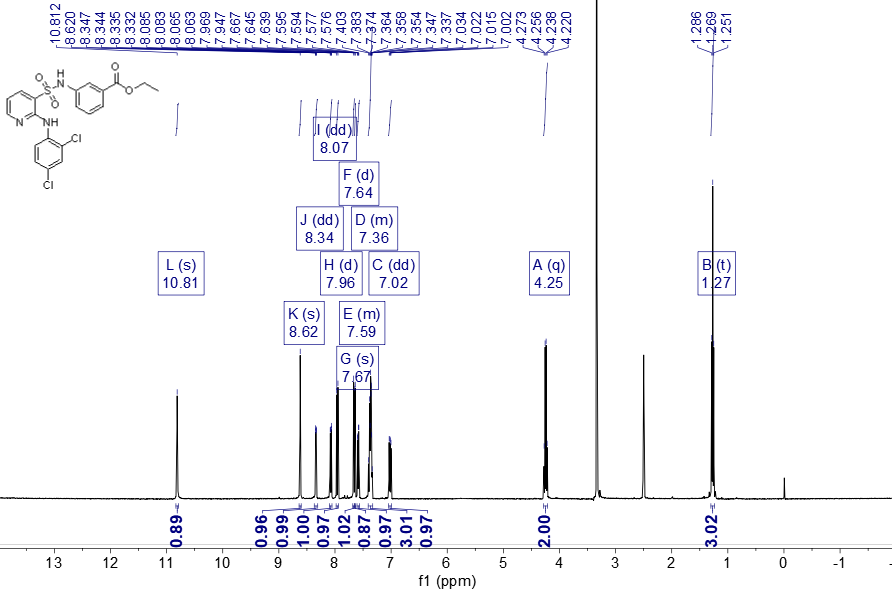


^1^H NMR of 8q


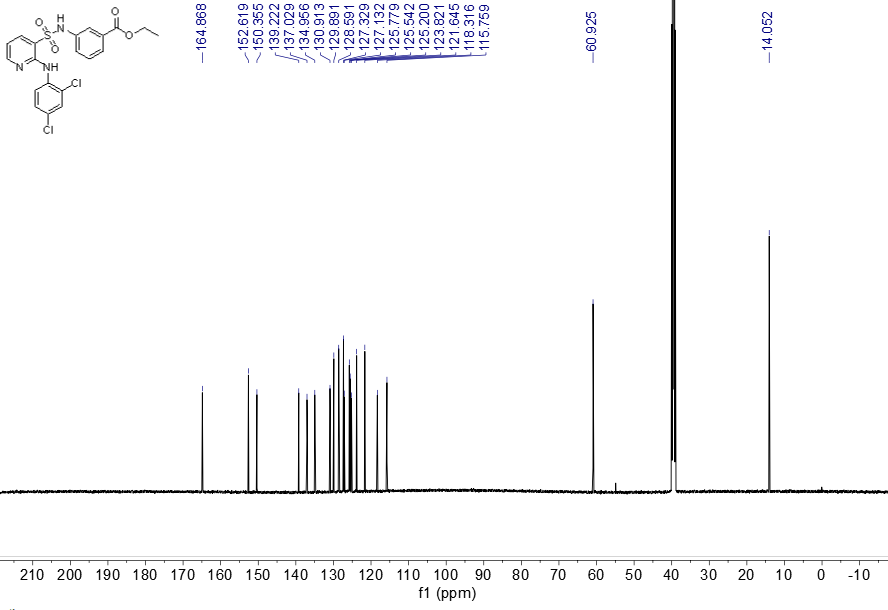


^13^C NMR of 8q


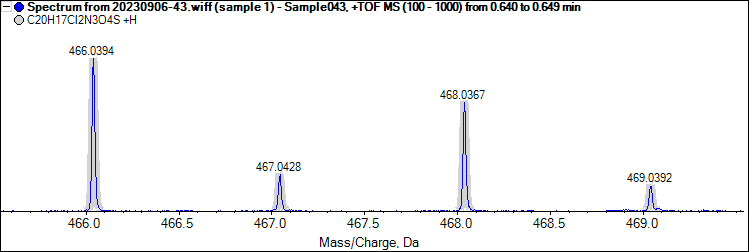


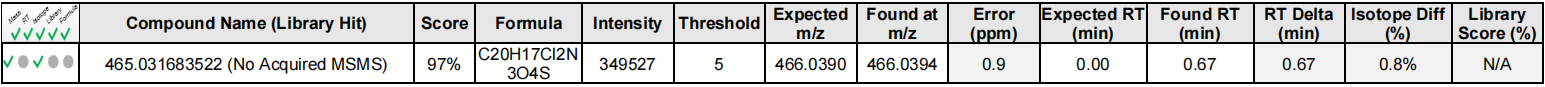


HRMS of 8q


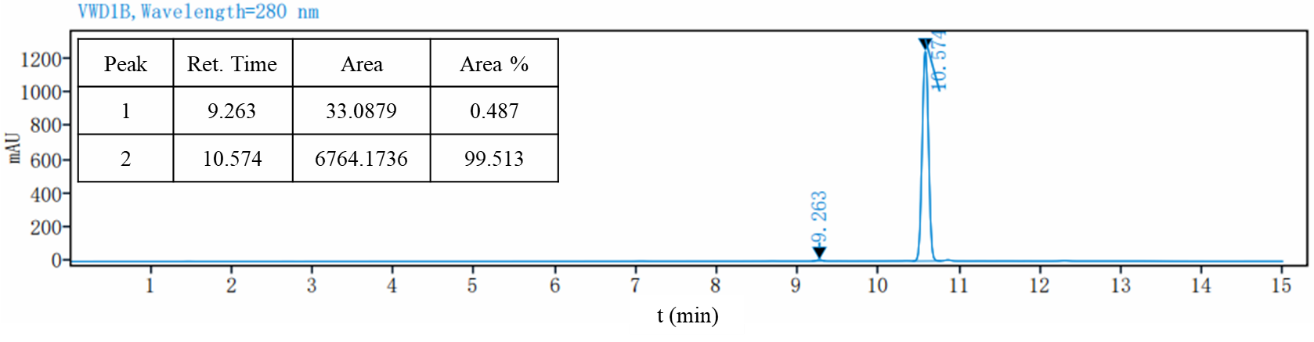


HPLC of 8q

^1^H NMR of 8r

^13^C NMR of 8r

HRMS of 8r

HPLC of 8r

^1^H NMR of 8s

^13^C NMR of 8s

HRMS of 8s

HPLC of 8s

^1^H NMR of 8t

^13^C NMR of 8t

HRMS of 8t

HPLC of 8t

^1^H NMR of 8u

^13^C NMR of 8u

HRMS of 8u

HPLC of 8u

^1^H NMR of 8v

^13^C NMR of 8v

HRMS of 8v

HPLC of 8v
